# Retinal sensitivity changes in early/intermediate AMD: a systematic review and meta-analysis of visual field testing under mesopic and scotopic lighting

**DOI:** 10.1038/s41433-024-03033-0

**Published:** 2024-03-18

**Authors:** Matt Trinh, Michael Kalloniatis, Sieu K. Khuu, Lisa Nivison-Smith

**Affiliations:** 1 School of Optometry and Vision Science, University of New South Wales, Sydney, NSW Australia; 2 School of Medicine (Optometry), Deakin University, Geelong, VIC Australia

**Keywords:** Physical examination, Macular degeneration, Retinal diseases

## Abstract

Visual fields under mesopic and scotopic lighting are increasingly being used for macular functional assessment. This review evaluates its statistical significance and clinical relevance, and the optimal testing protocol for early/intermediate age-related macular degeneration (AMD). PubMed and Embase were searched from inception to 14/05/2022. All quality assessments were performed according to GRADE guidelines. The primary outcome was global mean sensitivity (MS), further meta-analysed by: AMD classification scheme, device, test pattern, mesopic/scotopic lighting, stimuli size/chromaticity, pupil dilation, testing radius (area), background luminance, adaptation time, AMD severity, reticular pseudodrusen presence, and follow-up visit. From 1489 studies screened, 42 observational study results contributed to the primary meta-analysis. Supported by moderate GRADE certainty of the evidence, global MS was significantly reduced across all devices under mesopic and scotopic lighting with large effect size (−0.9 [−1.04, −0.75] Hedge’s g, *P* < 0.0001). The device (*P* < 0.01) and lighting (*P* < 0.05) used were the only modifiable factors affecting global MS, whereby the mesopic MP-1 and MAIA produced the largest effect sizes and exceeded test-retest variabilities. Global MS was significantly affected by AMD severity (intermediate versus early AMD; −0.58 [−0.88, −0.29] Hedge’s g or −2.55 [3.62, −1.47] MAIA-dB) and at follow-up visit (versus baseline; −0.62 [−0.84, −0.41] Hedge’s g or −1.61[−2.69, −0.54] MAIA-dB). Magnitudes of retinal sensitivity changes in early/intermediate AMD are clinically relevant for the MP-1 and MAIA devices under mesopic lighting within the central 10° radius. Other factors including pupil dilation and dark adaptation did not significantly affect global MS in early/intermediate AMD.

## Abstract (zh)

中明视和暗视照明下的视野越来越多地用于黄斑功能评估。这篇综述评估了其统计学意义和临床相关性, 以及早期/中期年龄相关性黄斑变性 (AMD) 的最佳检测方案。检索PubMed和Embase自建库起至2022年5月14日的文献。所有质量评估均根据GRADE指南进行。主要结局是全局平均敏感性 (MS), 进一步meta分析指标包括: AMD分类方案、设备、测试模式、中位/暗位照明、刺激大小/色度、瞳孔扩张、测试半径 (面积) 、背景亮度、适应时间、AMD严重程度、网状假性玻璃膜疣的存在和随访。我们对筛选的 1489 项中的42 项观察性研究结果进行了meta分析。在中等GRADE质量证据的支持下及在中明视和暗视照明下, 所有设备的全、全视野MS均显著降低 (−0.9 [−1.04, −0.75] Hedge’s g, *P* < 0.0001) 。所使用的设备 (*P* < 0.01) 和照明 (*P* < 0.05) 是影响全视野MS的唯一可改变的变量, 因此中视MP-1和MAIA受影响最大, 并超过了重新测试的可变性。AMD严重程度 (中度 vs 早期AMD;−0.58 [−0.88, −0.29] Hedge’s g或−2.55[3.62, −1.47]MAIA-dB) 和随访 (与基线相比;−0.62[−0.84, −0.41]Hedge’s g或−1.61[−2.69, −0.54]MAIA-dB) 对全视野MS有显著影响。早期/中期AMD的视网膜敏感性变化幅度与MP-1和MAIA装置在中心10°半径内的中明视照明下具有临床相关性。瞳孔扩张和暗适应等其他因素对早期/中期AMD的全视野MS没有显著影响。

## Introduction

Vision-related, functional changes in age-related macular degeneration (AMD) begin from the early stages [1–4] and eventually lead to severe functional and quality-of-life (QoL) impairment [5–8]. Consequently, many studies [9–20] including recent interventional trials [18, 21] have proposed using visual fields for functional testing of AMD due to its sensitive, repeatable, and clinically accessible nature [14]. Visual fields have facilitated greater understanding of the vision-related spatial function and QoL impact of AMD [22]. However, its “…role in clinical practice has yet to be specifically defined” [23], and there is a paucity of protocol standardisation across research groups utilising visual field assessment under mesopic or scotopic lighting for AMD.

It is essential that the results between individual studies be formally meta-analysed for clear interpretation and future development of standardised guidelines. Regarding the use of visual field testing under mesopic/scotopic lighting for early/intermediate AMD, however, it has not yet been determined whether mean sensitivity changes across studies are statistically significant and clinically relevant. Specifically, statistical significance denotes an arbitrary mean sensitivity difference and sample size between AMD and normal eyes, while the clinical relevance requires effect sizes to at least exceed test-retest variability [24]. It is also unclear how the many aspects of visual field testing such as the device [25–27], test pattern [14], stimuli size [28–32] and chromaticity [33, 34], pupil size [35], eccentricity [36–38], and background adaptation time [39, 40] used may influence outcomes.

Thus, this systematic review and meta-analysis aims to consolidate the literature to facilitate the highest level of evidence [41] to address: 1) whether visual field defects under mesopic and scotopic lighting are statistically significant and clinically relevant for use in early/intermediate AMD [42–44], and 2) what the optimal testing protocol is for early/intermediate AMD.

## Methods

This systematic review was registered prospectively via the International Prospective Register of Systematic Reviews (PROSPERO, CRD42022333929) [45] without amendment, and adhered to the reporting guidelines of the Preferred Reporting Items for Systematic Reviews and Meta-Analyses (PRISMA) statement [46].

### Eligibility criteria and literature search

Included studies performed visual fields (automated perimetry) under mesopic (0.005 to 5 cd/m^2^) or scotopic ( < 0.005 cd/m^2^) conditions [47–51] on comparative groups including treatment-naïve early and/or intermediate AMD eyes. Visual field testing was any systematic testing on a device that measured differential light sensitivities across pre-determined spaces in the visual field [52], including but not limited to commercial or modified static perimetry, flicker perimetry, and microperimetry/fundus-controlled perimetry. Early and intermediate AMD were defined according to the Beckman Initiative classification [53], although any study that used a commensurate classification was included [54, 55].

Literature searches were defined a priori [45] and performed via PubMed and Embase (OVID) for all published journal articles in English from inception to 14^th^ May 2022, using the respective search strings: “macular degeneration”[MeSH Terms] AND (“visual field tests”[MeSH Terms] OR “visual fields”[MeSH Terms] OR “contrast sensitivity”[MeSH Terms] OR “flicker fusion”[MeSH Terms]); and (exp age-related macular degeneration/ OR exp retina macula age-related degeneration/) AND (exp visual field/ or exp visual field defect/ OR exp perimeter/ OR exp perimetry/ OR exp contrast sensitivity/ OR exp critical flicker fusion/)). Reference lists of included studies and relevant review studies were searched. Search results were exported into Zotero 6.0.7 (Corporation for Digital Scholarship, VA, USA), duplicates were removed, and unique results exported into Microsoft Excel Version 2107 (Microsoft Corporation, WA, USA).

### Outcomes

The primary outcome was global mean sensitivity (MS), defined as average sensitivity in decibels (dB) across a total retinal area [52], in early/intermediate AMD versus normal eyes. Alternate labels for global MS with identical definitions, e.g., average threshold, were included. The secondary outcome was any real-world patient outcome such as quality of life and/or activities of daily living indices.

### Study selection

Unique search results were screened independently by two authors (MT and LNS) for title/abstract then full text if required. All discrepancies for study selection and assessments were resolved by discussion and consensus.

### Data extraction and quality assessment

Included studies underwent quality assessment by MT and LNS according to an adaptation of the ‘Users’ Guides to the Medical literature’ [44, 56]. This included consideration of study validity, results, and external validity [44, 56]. The data extracted for assessment is seen in Supplementary Table 1.

Risk of bias was assessed unblinded [57] according to the ‘QUADAS-2: A revised Tool for the Quality Assessment of Diagnostic Accuracy Studies’ [58] and ‘Newcastle-Ottawa Scale for Assessing the Quality of Non-randomised Studies in Meta-analyses’ [59]. The overall certainty of the evidence was assessed independently using the GRADE approach for observational studies [60, 61]. Qualitative data synthesis was summarised in tables of relevant study characteristics and outcomes, funding and conflict of interest statements, risk of bias assessment, and GRADE assessment.

### Data synthesis and quantitative assessment

Quantitative data synthesis and analysis, i.e., meta-analyses, were performed using Meta-Essentials (Erasmus Research Institute of Management, Rotterdam, The Netherlands) [62] and Review Manager (RevMan) version 5.4.1 (The Cochrane Collaboration, 2020) where at least three individual results per group or sub-group were available. Studies with identical data (e.g., from one clinical trial across multiple studies) were used once. Primary meta-analysis was followed by further meta-analyses according to between-study sub-groups, meta-regressions, and within-study sub-groups. Uni-variable (linear) meta-regression was performed when ≥10 studies [63] were pooled [64], and when pooling studies for longitudinal follow-up versus baseline meta-analysis. Time to follow-up visit (years) was used as the moderator in lieu of determining correlation coefficients (r) for dependent data. Sensitivity analyses were then performed to assess the robustness of all meta-analyses [63]:

- With repeated populations (tested under varying conditions) versus without repeated populations. Within study sub-groups [65, 66], data was selected from conditions expected to produce more conservative measures. Between study sub-groups, data was used from the larger sample to mitigate under-sampling/under-powering [67].
- With both eyes sampled versus without both eyes sampled.
- With no age-adjusted/controlled versus age-adjusted/controlled groups.
- With no cataract/pseudophakic-adjusted/controlled versus cataract/pseudophakic-adjusted/controlled groups.

Effect sizes were calculated using a random-effects model considering studies’ various contexts of data collection (mostly convenience samples) [68] and reported as standardised mean differences (Hedge’s g [95% CI]), where >0.8 Hedge’s g was considered a large effect size [41]. An outline of meta-analysis statistical interpretations is presented in Supplementary Table 2. Sub-groups’ effect sizes and heterogeneity were compared using Cochran’s Q test [69, 70], with *P* values considered after Bonferroni correction for multiple sub-group comparisons [71]. Publication bias was assessed using a funnel plot with trim and fill method using the linear estimator for missing studies, confirmed via Egger regression (testing significance between effect sizes and standard error) [72, 73] and Begg and Mazumdar rank correlation (testing significance between ranks of effect sizes and ranks of variance) [73, 74]. Statistical significance was defined as *P* < 0.05.

## Results

### Eligible studies

**Fig. 1:**
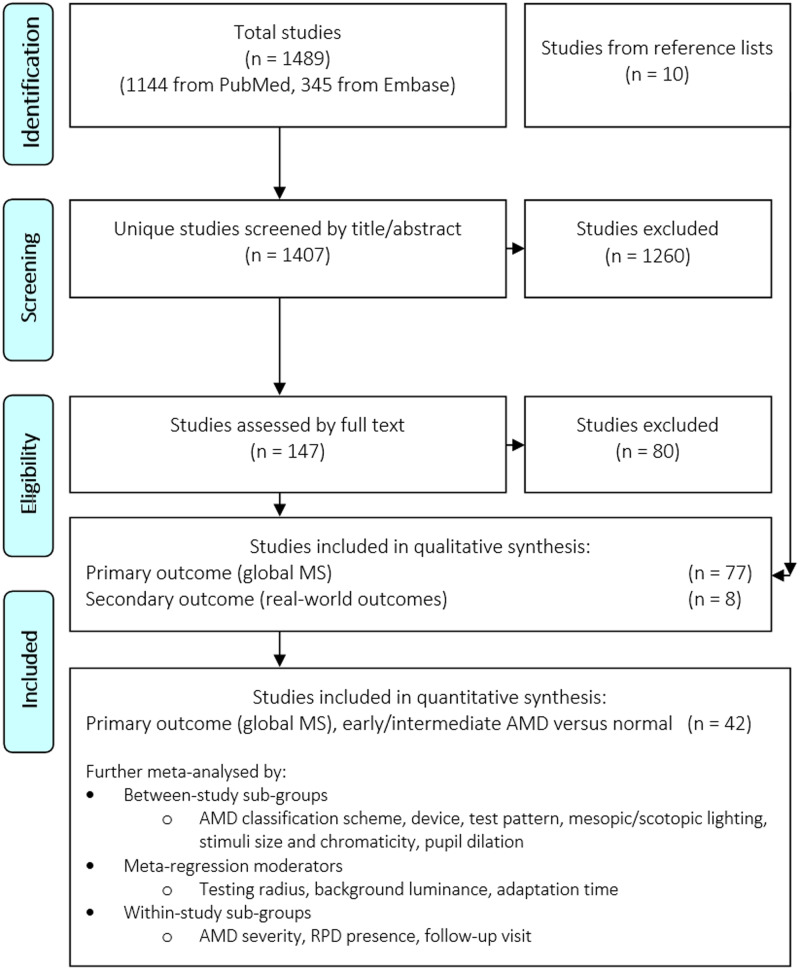
Preferred reporting Items for systematic reviews and meta-analyses (PRISMA) flow diagram for study selection and analyses.

Searches resulted in 1489 studies and 1407 unique studies. Full-text screening was performed for 147 studies, and 36 studies underwent two rounds of discussion before consensus (Fig. 1). A total of 67/147 studies were included from electronic database searches (reasoning for exclusions in Supplementary Table 3). Searches through reference lists of included studies and relevant review studies yielded 10 additional eligible studies [75–85]. Thus, 77 studies were included in this review (Fig. 1).

### Qualitative assessment

A detailed summary of all study characteristics and outcomes is presented in Supplementary Table 4, with reported source(s) of funding (65/77 studies) and potential conflict(s) of interest (67/77 studies) presented *verbatim* in Supplementary Table 5. All study designs were observational. The most commonly used AMD classification scheme was from the Beckman Initiative [53] (39/77). AMD and normal group sample sizes ranged from three to 319 eyes. Some studies used multiple testing conditions on a single population, i.e., from 77 studies there were 101 different visual field protocols. The most used device was the Macular Integrity Assessment (MAIA; 37/101). Background luminance was more commonly set as mesopic (0.801 to 5 cd/m^2^, 74/101) than scotopic lighting (0 or 0.0032 cd/m^2^, 27/101).

Most studies (74/77) had at least one domain with a high risk of bias (Supplementary Table 6). For the domain ‘patient selection’, 64/77 studies had a high risk of bias while for the domain ‘comparability of study groups’, 35/77 had a high risk of bias.

### Quantitative assessment

**Fig. 2:**
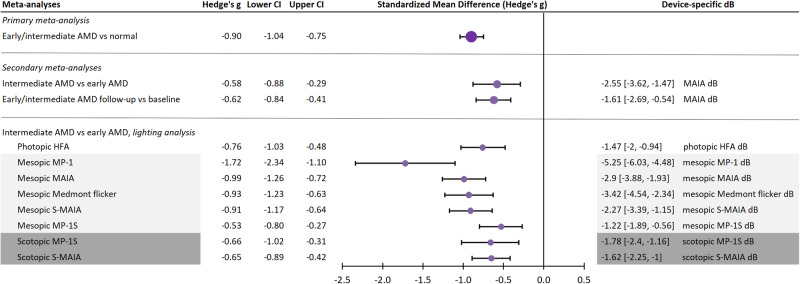
Summary forest plot of meta-analyses. Negative values indicate reduced global MS. Summary data presented as standardised Hedge’s g [CI] and converted to device-specific dB where available. For meta-analysis between lighting conditions, photopic device was depicted in *white*, mesopic devices in *light grey*, and scotopic devices in *dark grey*. Note devices can have more than one background luminance setting. The full forest plots for each meta-analysis are presented in Supplementary Figs. 1–5. Vs versus, i.e., meta-analysis comparison.

A summary forest plot of meta-analyses is presented in Fig. 2, with full forest plots available in Supplementary Figs. 1–5.

### Primary meta-analysis – early/intermediate AMD versus Normal

From 77 eligible studies, 42 provided adequate data for the calculation of global MS between early/intermediate AMD versus normal eyes. Most studies excluded from primary meta-analysis were due to not having a comparative normal group (Supplementary Table 4). Global MS in early/intermediate AMD eyes (*n* = 2587) was significantly reduced compared to normal eyes (*n* = 1348) with a large effect size of −0.9 [−1.04, −0.75] Hedge’s g (*P* < 0.0001), which may have represented substantial heterogeneity (Q(41) 133.04, *P* < 0.0001, Tau^2^ 0.14, I^2^ 69%; Fig. 2 and Supplementary Fig. 1).

### Potential confounding

Further meta-analyses by within-study sub-groups were performed to identify potential confounding (categorical) factors, including AMD classification scheme, device, test pattern, mesopic/scotopic lighting, stimuli size and chromaticity, and pupil dilation. Only the device used significantly affected outcomes for early/intermediate AMD versus normal (*P* < 0.01, Supplementary Table 7). All subsequent analyses were then adjusted for the device used, and included conversion from Hedge’s g to device-specific dB for clinical context [32, 86].

Meta-regression was also performed to identify potential confounding (continuous) factors, including testing radius (area), background luminance, and adaptation time. None of these factors significantly affected outcomes for early/intermediate AMD versus normal (*P* = 0.24 to 0.84, Supplementary Table 7).

### Dose-response relationships

Meta-analyses by between-study sub-groups were then performed to assess dose-response relationships. This included comparisons of global MS against: AMD severity, reticular pseudodrusen (RPD) presence, and at longitudinal follow-up versus baseline.

For AMD severity, a comparison of intermediate AMD versus early AMD (Fig. 2 and Supplementary Fig. 2) demonstrated significantly reduced global MS with a medium effect size of −0.58 [−0.88, −0.29] Hedge’s g. Converted to device-specific dB, this was equivalent to −2.55 [−3.62, −1.47] MAIA-dB, *P* < 0.001 (Q(5) 10.43, *P* = 0.06, Tau^2^ 0.07, I^2^ 52.88%; *n* = 465 intermediate AMD versus 247 early AMD). Only one device sub-group could be formed using the MAIA.

For RPD presence versus absence, a meta-analysis could not be formed due to an insufficient number of results per device sub-group. The five individual results using the Medmont DACP, MAIA, and MP-1 devices showed generally reduced global MS in the presence of RPD (−0.58 [−1.1, −0.07] to −1.55 [−2.43, −0.68] Hedge’s g; Supplementary Fig. 3).

For longitudinal follow-up versus baseline (Fig. 2 and Supplementary Fig. 4), global MS was significant reduced with a medium effect size of −0.62 [−0.84, −0.41] Hedge’s g or −1.61 [−2.69, −0.54] MAIA-dB, *P* < 0.0001 (Q(3) 4.5, *P* = 0.21, Tau^2^ 0.02, I^2^ 33%; *n* = 260 follow-up versus 426 baseline) though only one device sub-group could be formed using the MAIA. Additional adjustment for follow-up time via meta-regression showed the rate of change to be −0.09 [−0.28, 0.1] Hedge’s g or −0.23 [−0.9, 0.13] MAIA-dB per year, without statistical significance (*P* = 0.12).

### Post hoc lighting analysis (photopic versus mesopic versus scotopic)

To explore the modulating effect of lighting against current clinical visual field devices which operate under photopic lighting, external meta-analysis data from photopic visual field testing was included [87] with adjustments for the device used. There were significant sub-group differences between photopic (*n* = 234 AMD and 221 normal) versus mesopic (*n* = 1777 AMD and 922 normal) versus scotopic (*n* = 353 AMD and 341 normal) results (*P* < 0.05, Fig. 2 and Supplementary Fig. 5). The greatest effect sizes (with varying heterogeneities) were observed from the mesopic MP-1 (−1.72 [−2.34, −1.1] Hedge’s g; −5.25 [−6.03, −4.48] MP-1 dB) and MAIA devices ((−0.99 [−1.26, −0.72] Hedge’s g; −2.9 [−3.88, −1.93] MAIA dB).

### Sensitivity analysis

Sensitivity analysis revealed robust meta-analyses results, whereby inclusion versus exclusion of meta-data with repeated populations (tested under varying conditions), both eyes (rather than single eyes) sampled, no age-adjusted/controlled (versus age-adjusted/controlled) groups, and no cataract/pseudophakic-adjusted/controlled (versus cataract/pseudophakic-adjusted/controlled) groups, did not alter any effect sizes (*P* = 0.38 to 1).

### Publication bias

The funnel plot of the primary outcome demonstrated slight asymmetry to the left, implying that effect sizes were borderline biased towards being over-estimated (Egger regression *P* = 0.17, Begg and Mazumdar rank correlation *P* = 0.03; Supplementary Fig. 6). Four theoretical individual results were imputed to the right from trim and fill analysis, resulting in a medium adjusted effect size of −0.57 [−0.75, −0.4] Hedge’s g, rather than a large observed effect size of −0.9 [−1.04, −0.75] Hedge’s g for primary meta-analysis.

### GRADE assessment and summary of findings

Studies included in primary meta-analysis demonstrated an overall moderate level of certainty of evidence, that global MS was significantly reduced across all devices under mesopic and scotopic lighting with large effect size (Supplementary Table 8) [60, 61].

### Secondary outcome – real-world patient outcomes

Eight studies (one with identical data) [88, 89] reported a linkage between the mesopic global MS in early/intermediate AMD and any real-world patient outcome (Supplementary Table 9), the latter of which was derived from three unique questionnaires [90–92] and two series of computerised visual tasks [93, 94]. Generally, correlations ranged from non-significant to moderate, and results were unclear whether microperimetry reflected these outcomes better, worse, or similar to other diagnostic modalities.

## Discussion

This systematic review and meta-analysis found that with moderate GRADE certainty of the evidence, global retinal sensitivity changes under mesopic and scotopic lighting are statistically significant in early/intermediate AMD. Specifically, the magnitudes of changes are clinically relevant for the mesopic MP-1 and MAIA devices within the central 10° radius. The device and adapting light levels used were the only modifiable factors affecting outcomes, with other commonly considered factors such as pupil dilation and dark adaptation time insignificantly affecting global MS. Further research is needed to understand how mesopic and scotopic testing may link to real-world patient outcomes.

### Visual fields under mesopic lighting is statistically significant and clinically relevant for AMD assessment

Our results highlighted that most visual field devices under mesopic lighting were statistically valid for the assessment of early/intermediate AMD [42, 95]. Specifically, eyes with intermediate AMD demonstrated reduced global MS that was statistically significant and large effect sized (>0.8 Hedge’s g) [96–98] for the MP-1, MAIA, Medmont flicker, and S-MAIA devices under mesopic lighting. This was strengthened by dose-dependent relationships, however, a significant yearly rate of change could not be established due to a paucity of studies with longer (than mostly 12month) follow-up times. More longitudinal studies are required to extrapolate the optimal frequency with which visual fields under mesopic lighting may need to be performed in patients with AMD.

It is well established that effect sizes are a statistical concept and regardless of the large magnitudes we observed, findings should be further interpreted in a clinical context [99]. For visual fields, minimal clinical significance is commonly defined as sensitivities exceeding visual field device/protocol test-retest variability [100]. From this review, the mean sensitivity losses detected by the MP-1 (−1.72 Hedge’s g or −5.25 dB) and MAIA (−0.99 Hedge’s g or −2.9 dB) exceeded intra-session and inter-session test-retest variabilities of corresponding populations of 1.1 to 1.56 dB (Supplementary Table 10) [101, 102]. Whilst other devices such as the Medmont flicker and S-MAIA also demonstrated large effect sizes, direct interpretation of their clinical relevance was limited by a lack of relevant test-retest variability data in normal and early/intermediate AMD populations.

### Standardisation of visual field protocol for early/intermediate AMD requires consideration of the device and lighting used

The validation of a ‘new diagnostic’ test requires methodological standardisation [103, 104] to ensure consistent quality of output and efficient use of resources (especially time) [105–107]. This review assessed if the selected device [25–27], test pattern [14], stimuli size [28–32] and chromaticity [33, 34], pupil size [35], light levels [108, 109], eccentricity [36–38], and background adaptation time [39, 40] may affect global MS in the early/intermediate AMD population, to determine the optimal testing protocol. At the summary level, we found that almost all the above modifiable factors were insignificant, as validated in other studies [14, 110, 111]. This is particularly relevant for research groups seeking to validate functional endpoints for AMD (e.g., the ALSTAR2 [20] and MACUSTAR groups) [112], whereby the time saved from not performing protracted dark adaptation for mesopic testing, in particular, could be redirected elsewhere. Consistent application of testing protocol at the individual patient level (within populations) is likely still important [14, 25–30, 32–40, 108, 109].

The only modifiable factors that significantly altered effect sizes were the device and lighting used between studies. These effects ranged from very large in the MP-1 to medium in the MP-1S, despite very similar testing properties (see Pfau et al. [14] for a detailed summary of inter-device differences). This has been corroborated within study populations showing inter-device differences in measured outcomes [25–27]. This may reflect effects from other parameters such as test pattern design [113], eye/image tracking frequency and software, ambient screen lighting, calibration and precision, etc., or simply be due to the under-sampling of meta-analysis sub-groups [67]. Nevertheless, caution is recommended when comparing results between devices in practice. Continued development of visual field technology and processes including time-saving threshold estimation strategies [114], test grid density [115], and frontloading tests [116, 117] will also need to be considered for further optimisation of visual field testing in AMD.

### Understanding the need to test under mesopic lighting in AMD

We confirmed that visual field defects were generally greater under mesopic lighting than photopic lighting, supporting the concept that photoreceptor diseases such as AMD are light-adaptation dependent. That is, functional defects can be more greatly appreciated under lower (mesopic or scotopic versus photopic) light levels, previously modelled as the threshold-versus-intensity curve (Supplementary Fig. 7) [50, 108, 118, 119]. Greater threshold changes under mesopic versus photopic lighting may also have related to changes in spatial summation, which alters with differing background luminances [120, 121].

Interestingly, rod susceptibility in AMD [122–124] implied that differences in retinal sensitivity should have also been greater when probing rod (scotopic) versus cone (mesopic) function. However, there were no differences in global MS when comparing scotopic versus mesopic lighting [125, 126], nor when comparing cyan versus red stimuli [127–129], even when repeating analyses using within-study sub-groups to eliminate between-study differences in the testing protocol (*data not shown*). Effect sizes were also, surprisingly, generally greater under mesopic than scotopic lighting. These findings were likely influenced by the visual field testing patterns in this review which did not capture areas of peak rod density beyond the macula [123, 124]. Whilst we could not draw definitive conclusions regarding whether mesopic testing was superior to scotopic testing due to potential under-powering of our sub-group and *post hoc* analyses [67], the evidence supports the need for testing under lower (than photopic, standard automated perimetry [10 cd/m^2^]) light levels, which can be accessed through most microperimeters (1.27 cd/m^2^ and below) [14], the Octopus perimeter (1.27 cd/m^2^) [35], the Medmont perimeter (3.2 cd/m^2^) [35], and virtual reality-based perimetry (1 cd/m^2^) [130].

### Limitations and future research

The main limitation of this review lies in the argument that there are several other less onerous measures of low-light function such as low-luminance visual acuity and deficit which may produce similar sensitivity/specificity regarding AMD [131]. However, these measures are yet to be systematically reviewed and are spatially indiscriminate. Visual fields can map functional changes in concordance with spatial patterns of AMD structural changes [132–134], providing spatial structure-function context as to which neuronal/synaptic, vascular, and/or physiological mechanisms may be impacted. As aforementioned, visual fields have not necessarily been propositioned to replace other diagnostic tools (such as colour fundus photography) for early/intermediate AMD, but rather as a supplementary tool to enhance diagnostic confidence and provide prognostic information in consideration of patients’ visual function and QoL [42, 95].

Additionally, there was a dearth of data regarding downstream effects of performing visual fields on patient-relevant outcomes [42, 44, 135]. The data suggested that current measures of patient-relevant outcomes in the early stages of AMD may be insensitive to the magnitude and/or scope of changes, though this does not preclude patient relevance, particularly as the disease progresses in severity. Research that explores the advantage of performing a spatially delineated functional test in association with more practical scenarios, e.g., whether visual fields can predict orientation and mobility [136] or driving capabilities [137–140], could be more beneficial in future.

## Conclusions

This systematic review highlighted with moderate certainty of the evidence, that global retinal sensitivity changes under mesopic and scotopic lighting are statistically significant in early/intermediate AMD. Specifically, the magnitudes of changes are clinically relevant for the mesopic MP-1 and MAIA devices within the central 10° radius. Other factors such as pupil dilation and dark adaptation did not significantly affect global MS and may be unnecessary to consider in future testing protocol of early/intermediate AMD eyes. Future research should explore how testing may link to real-world patient outcomes.

### Supplementary information

Supplementary material

PRISMA checklist

EYE reporting checklist

## Data Availability

Specific meta-analyses data is available upon reasonable request to the authors. Supplementary Material is available at Eye’s website.
