# Supplementary material for: Retinal sensitivity changes in early/intermediate AMD: a systematic review and meta-analysis of visual field testing under mesopic and scotopic lighting
Source: Eye (Lond). 2024 Mar 18;38(10):1827–35. doi: 10.1038/s41433-024-03033-0 (PMC11229509; doi:10.1038/s41433-024-03033-0)
Supplement: Supplementary file 1 — Supplementary material [file 41433_2024_3033_MOESM1_ESM.docx]

Retinal Sensitivity Changes in Early/Intermediate AMD: A Systematic Review and Meta-analysis of Visual Field Testing under Mesopic and Scotopic Lighting

Matt Trinh (PhD),^1^ Michael Kalloniatis (PhD),^1,2^ Sieu K. Khuu (PhD),^1^ and Lisa Nivison-Smith (PhD)^1^

*^1^School of Optometry and Vision Science, University of New South Wales, Sydney, NSW, Australia; ^2^School of Medicine (Optometry), Deakin University, Geelong, VIC, Australia.*

**Correspondence:** Dr Lisa Nivison-Smith; [l.nivison-smith@unsw.edu.au](mailto:l.nivison-smith@unsw.edu.au)

# Supplementary Tables

**Supplementary Table 1. Data extracted for assessment.**

| **Data extracted** | **Conditions** |
| --- | --- |
| Authors, publication date, and study design. | Single (rather than both) eye data per participant using simple randomisation was extracted if available.  Missing data were extracted from graphs using WebPlotDigitizer version 4.5 (California, USA)(1) or calculated from piece-wise data(2) assuming normality for sample sizes > 30 based on Central Limit Theorem.(3)  If missing data could not be extracted or calculated from the study, a request for the missing data was sent to the study’s authors via email. Authors were given one month (with a request for time extension possible) to respond. |
| AMD classification scheme, sample sizes, whether single or both eyes were sampled per participant, and whether age was controlled/adjusted between groups. |  |
| Visual field testing conditions, i.e., selected device, protocol, threshold strategy, stimuli size and chromaticity, background luminance, pupils dilated or undilated, whether practice exam was included, background adaptation time, stimulus chromaticity, and whether within-study sub-group analysis was available. |  |
| Primary outcome and secondary outcome availability. |  |
| Funding and conflict of interest statements. |  |

**Supplementary Table 2. Interpretation of meta-analyses statistics.**

| **Statistic** | **Definition** | **Interpretation** |
| --- | --- | --- |
| Hedge’s g(4) | Sample mean difference divided by SD_pooled_ (dB) with correction for small sample sizes (unlike Cohen’s d). | ≥ 0.2 = small  ≥ 0.5 = medium  ≥ 0.8 = large(5–7) |
| Heterogeneity(8–10) | Q = total variance  Tau^2^ = Q-transformed  I^2^ = percentage of variation that is indicative of true heterogeneity versus sampling error | Q = interpreted against degrees of freedom (number of studies – 1)  Tau^2^ = interpreted as variance (SD^2^) between studies  I^2^ = 0-40% ‘may not be important’, 30-60% ‘may represent moderate heterogeneity’, 50-90% ‘may represent substantial heterogeneity’, and 75-100% ‘may represent considerable heterogeneity’(10,11) |
| Correlations(12) | Linear relationship between two variables | \|r\| < 0.1 = negligible  < 0.4 = weak  < 0.7 = moderate  < 0.9 = strong  ≥ 0.9 = very strong(12) |

**Supplementary Table 3. Reasoning for exclusion.**

Primary reasoning for exclusion after full text screening.

| **Study** | **Primary reasoning** |
| --- | --- |
| **Acton et al. 2012**(13) | Not tested under mesopic or scotopic conditions |
| **Acton et al. 2013**(14) | Review |
| **Acton et al.**(15) | AMD and normal group tested under different conditions |
| **Anderson et al. 2011**(16) | Early/intermediate AMD not clearly separated from late AMD |
| **Atchison et al. 1990**(17) | Not tested under mesopic or scotopic conditions |
| **Barboni et al. 2018**(18) | Early/intermediate AMD not clearly separated from late AMD |
| **Birch et al. 2002**(19) | Not tested under mesopic or scotopic conditions |
| **Cassels et al. 2018**(20) | Review |
| **Chen et al. 1992**(21) | Early/intermediate AMD not clearly separated from late AMD |
| **Cheng et al. 1993**(22) | Not tested under mesopic or scotopic conditions |
| **Choi et al 2019**(23) | Not tested under mesopic or scotopic conditions |
| **Denniss et al. 2017**(24) | Early/intermediate AMD not clearly separated from late AMD |
| **Denniss et al. 2018**(25) | Early/intermediate AMD not clearly separated from late AMD |
| **Eisner et al. 1992**(26) | No automated perimetry |
| **Feher et al. 2005**(27) | Not tested under mesopic or scotopic conditions |
| **Feigl et al. 2004**(28) | Not tested under mesopic or scotopic conditions |
| **Feigl et al. 2005**(29) | Not tested under mesopic or scotopic conditions |
| **Forshaw et al. 2021**(30) | Not tested under mesopic or scotopic conditions |
| **Forte et al. 2014**(31) | Not tested under mesopic or scotopic conditions |
| **Fraser et al. 2016**(32) | Macula bleached |
| **Frennesson et al. 1995**(33) | Not tested under mesopic or scotopic conditions |
| **Gunawan et al. 2022**(34) | Macula bleached |
| **Guymer et al. 1997**(35) | Early/intermediate AMD not clearly separated from late AMD |
| **Hartmann et al. 2011**(36) | Not tested under mesopic or scotopic conditions |
| **Hassan et al. 2002**(37) | Late AMD only |
| **Hazel et al. 2000**(38) | Late AMD only |
| **Herse et al. 2005**(39) | Early/intermediate AMD not clearly separated from late AMD |
| **Kaiser et al. 1994**(40) | Early/intermediate AMD not clearly separated from late AMD |
| **Karadeniz Ugurlu et al. 2017**(41) | Not tested under mesopic or scotopic conditions |
| **Kim et al. 2015**(42) | Early/intermediate AMD not clearly separated from late AMD |
| **Kitano et al. 2021**(43) | Early/intermediate AMD not clearly separated from late (geographic atrophy) AMD |
| **Laishram et al. 2017**(44) | Early/intermediate AMD not clearly separated from late AMD |
| **Landa et al. 2011**(45) | Early/intermediate AMD not clearly separated from late AMD |
| **Lee et al. 2015**(46) | Not tested under mesopic or scotopic conditions |
| **Limoli et al. 2014**(47) | Early/intermediate AMD not clearly separated from late AMD |
| **Liu et al. 2015**(48) | Early/intermediate AMD not clearly separated from other macular disease |
| **Liu et al. 2017**(49) | Early/intermediate AMD not clearly separated from late AMD |
| **Markowitz et al. 2020**(50) | Late AMD only |
| **Mayer et al. 1992**(51) | Early/intermediate AMD not clearly separated from late AMD |
| **Mazzoli et al. 2019**(52) | Early/intermediate AMD not clearly separated from late AMD |
| **McGuinness et al. 2020**(53) | Macula bleached |
| **Midena et al. 1994**(54) | Not tested under mesopic or scotopic conditions |
| **Midena et al. 1997**(55) | Not tested under mesopic or scotopic conditions |
| **Midena et al. 2017**(56) | Review |
| **Narayanan et al. 2020**(57) | No automated perimetry |
| **Nebbioso et al. 2014**(58) | Pre-early AMD |
| **Neelam et al. 2009**(59) | Review |
| **Neely et al. 2017**(60) | Not tested under mesopic or scotopic conditions |
| **Nguyen et al. 2018**(61) | Macula bleached |
| **Owsley et al. 2016**(62) | Not tested under mesopic or scotopic conditions |
| **Owsley et al. 2016**(63) | Not tested under mesopic or scotopic conditions |
| **Pfau et al. 2021**(64) | Review |
| **Phipps et al. 2003**(65) | No automated perimetry |
| **Piccardi et al. 2009**(66) | Not tested under mesopic or scotopic conditions |
| **Querques et al.2012**(67) | Early/intermediate AMD not clearly separated from late AMD |
| **Querques et al. 2014**(68) | Not tested under mesopic or scotopic conditions |
| **Rai et al. 2021**(69) | No microperimetry and late AMD only |
| **Rovner et al. 2006**(70) | No automated perimetry |
| **Sabeti et al. 2021**(71) | Late AMD only |
| **Sandberg et al. 1998**(72) | Unspecified macular diagnoses |
| **Scorolli et al. 2003**(73) | Not tested under mesopic or scotopic conditions |
| **Shinojima et al. 2020**(74) | Early/intermediate AMD not clearly separated from pre-early AMD or other macular disease |
| **Steinberg et al. 2015**(75) | Early/intermediate AMD not clearly separated from late AMD |
| **Steinberg et al. 2017**(76) | Early/intermediate AMD not clearly separated from late AMD |
| **Sunness et al. 1988**(77) | No automated perimetry |
| **Tan et al. 2019**(78) | Macula bleached |
| **Tolentino et al. 1994**(79) | Not tested under mesopic or scotopic conditions |
| **Tran et al. 2010**(80) | Late AMD only |
| **Tran et al. 2012**(81) | Late AMD only |
| **Tran et al. 2015**(82) | Early/intermediate AMD not clearly separated from late AMD |
| **Vessey et al. 2018**(83) | Early/intermediate AMD not treatment-naïve |
| **Wang et al. 2012**(84) | Early/intermediate AMD not clearly separated from late AMD |
| **Weigert et al. 2011**(85) | Early/intermediate AMD not clearly separated from late AMD |
| **White et al. 2021**(86) | Early/intermediate AMD not clearly separated from late AMD |
| **White et al. 2022**(87) | Early/intermediate AMD not clearly separated from late AMD |
| **Wong et al. 2017**(88) | Review |
| **Wood et al. 2011**(89) | Early/intermediate AMD not clearly separated from late AMD |
| **Wood et al. 2018**(90) | Not tested under mesopic or scotopic conditions |
| **Yanagisawa et al. 2018**(91) | Late AMD |
| **Yang et al. 2021**(92) | Review |
| **Yow et al. 2017**(93) | Early/intermediate AMD not clearly separated from late AMD |

**Supplementary Table 4. Study characteristics and outcomes.**

Study design(94) – **^†^** denotes study that was re-classified based on data that were relevant to this review.(95–107)

Participants (cases and controls) – **^‡^** denotes studies that controlled or adjusted for age (or time for longitudinal data) between study groups; description of study eye selection provided; **^‖^** denotes studies that controlled or adjusted for cataract/phakic status between study groups.

Testing conditions – **^◊^** denotes default testing conditions if not otherwise explicitly mentioned; § denotes additional testing conditions if mentioned. ‘Autoflicker’ refers to 18Hz at 1° and 3°, 16Hz at 6°, and 12Hz at 10°.

Primary and secondary outcome available? – yes, yes (calculated [using piece-wise data]), or no. Within-study sub-group analyses also described if available. Studies without a comparative group, e.g., cross-sectional studies, were not searched for primary outcome.

Specific meta-analyses data is available upon reasonable request to the authors.

Abbreviations: AREDS, Age-Related Eye Disease Study; ARMD/AMD, age-related macular degeneration; DACP, dark-adapted chromatic perimeter; DPED, drusenoid pigment epithelial detachment; early/intermediate AMD, early and/or intermediate age-related macular degeneration; FAF, fundus autofluorescence; GA, geographic atrophy; GI…V, Goldmann size 1…5; ICGS, International Classification and Grading System for ARM and AMD; IS/OS, inner segment/outer segment layers; LEAD, Laser Intervention in Early Stages of Age-related Macular Degeneration; MAIA, Macular Integrity Assessment; MD, mean deviation; MP, Microperimeter or microperimetry; MS, mean sensitivity; OTI, Ophthalmic Technologies Incorporated; PSD, pattern standard deviation; RPD, reticular pseudodrusen; RPE, retinal pigment epithelium; S, scotopic; SDD, sub-retinal drusenoid deposits; Td, Trolands; VA, visual acuity.

| **Study** | **Study design** | **Early/intermediate AMD classification** | **N eyes [/participants]; Cases** | **N eyes [/participants]; Controls** | **Testing conditions** | **Primary outcome (global MS) available?** | | **Secondary outcome (linkage to quality of life or activities of daily living indices) available?** |
| --- | --- | --- | --- | --- | --- | --- | --- | --- |
|  |  |  |  |  | **Device, protocol**  **Threshold strategy, stimulus size, background luminance** | **Cases** | **Controls** |  |
| **Acton et al. 2012**(15)  **[controls from Acton et al. 2011]**(108) | Case-control | ICGS:(109) early + intermediate | 21 [/21]; dominant eye used | 50 [/50]**^‡‖^**; dominant eye used | MP-1, 68 stimuli within 10° radius  Full threshold, GIII, 1.27cd/m^2^  § Pupils dilated in cases; pupils undilated in controls  § Background adaptation 15 minutes  § Practice exam included | Yes (calculated) | Yes | No |
| **Adams et al. 2018**(110) | Cross-sectional | Beckman Initiative:(111) intermediate | 79 [/38]; both eyes eligible | - | iPad; PsyPad App, 12 stimuli within 2° radius  Full threshold, GIII, 1.27cd/m^2^ | - | - | No |
|  |  |  |  |  | MAIA, 37 stimuli within 6° radius  Full threshold, GIII, 1.27cd/m^2^  § Practice exam included  § Analysis available within 2.3° radius only | - | - | No |
| **Broadhead et al. 2017**(112) | Case-control | AREDS 4 step:(113) early + intermediate | 72 [/51];  both eyes eligible | 36 [/18]**^‡^**;  both eyes eligible | MAIA, 37 stimuli within 5° radius  Full threshold, GIII, 1.27cd/m^2^  § Pupils dilated | Yes | Yes | No |
| **Broadhead et al. 2020**(114) | Cross-sectional | AREDS 4 step:(113) early + intermediate | 128 [/64];  both eyes eligible | - | MAIA, 37 stimuli within 5° radius  Full threshold, GIII, 1.27cd/m^2^  § Pupils dilated | - | - | Yes |
| **Chandramohan et al. 2016**(115) | Case-control | Beckman Initiative:(111) early + intermediate | 20 [/20]; better VA eye used, otherwise randomised by birth month | 10 [/10]**^‡‖^**; better VA eye used, otherwise randomised by birth month | MAIA, 37 stimuli within 5° radius  Full threshold^◊^, GIII^◊^, 1.27cd/m^2◊^  § Pupils dilated  § Practice exam included  § Sub-groups: early (n = 8 [/8]) and intermediate (n = 12 [/12]) AMD | No; within-study sub-group analysis possible | No | No |
| **Chen et al. 2004**(116) | Case-control | ‘dry-form ARMD’ | 24 [/24]; unspecified eye used | 16 [/16]; unspecified eye used | Octopus, 81 stimuli within 25° radius  Full threshold, GV, 0cd/m^2^  § Pupils dilated  § Background adaptation 40 minutes  § Wratten blue filter used (W47b) | Yes | Yes | No |
| **Clemens et al.**  **2015**(117) | Cross-sectional | ‘DPED with AMD’ | 13 [/10]; both eyes eligible | - | MAIA, 37 stimuli within 5° radius  Full threshold^◊^, GIII^◊^, 1.27cd/m^2◊^  § Pupils dilated | - | - | No |
| **Cocce et al. 2018**(118) | Case-control | AREDS 4 step:(113) early + intermediate | 79 [/79]; better VA eye used, otherwise random eye used | 20 [/20]**^‡‖^**; better VA eye used, otherwise random eye used | MAIA, 37 stimuli within 5° radius  Full threshold, GIII, 1.27cd/m^2^  § Pupils dilated  § Sub-groups: early (n = 32 [/32]) and intermediate (n = 47 [/47]) AMD | Yes (calculated); within-study sub-group analysis possible | Yes | No |
| **Corvi et al. 2019**(119) | Cross-sectional^†^ | ‘early AMD’ | 15 [/15]; unspecified study eye used | - | MAIA, 6 stimuli within 5° eccentricity and 10 stimuli within 10° eccentricity  Full threshold, GIII, 1.27cd/m^2^  § Practice exam included | -; within-study sub-group analysis possible | - | No |
|  |  |  |  |  | MP-1, 6 stimuli within 5° eccentricity and 10 stimuli within 10° eccentricity  Full threshold, GV, 0.0032cd/m^2^  § Background adaptation 35 minutes | -; within-study sub-group analysis possible | - | No |
| **Dinc et al. 2008**(120) | Case-control | ‘intermediate AMD’ | 30 [/30]; random eye used | 30 [/30]**^‡^**; random eye used | MP-1, 76 stimuli within 10° radius  Full threshold, GIII, 1.27cd/m^2^  § Pupils dilated  § Background adaptation 15 minutes  § Practice exam included | Yes | Yes | No |
| **Dow et al. 2016**(103) | Cross-sectional^†^ | AREDS 4 step:(113) early + intermediate | Unspecified [/unspecified]; both eyes eligible from placebo group | - | MAIA, 61 stimuli within 10° radius  Full threshold, GIII, 1.27cd/m^2^  § Practice exam included | - | - | No |
| **Echols et al. 2020**(121) | Case-control | AREDS 9 step:(122) early + intermediate | 47 [/47]; unspecified study eye used | 28 [/28]**^‡^**; unspecified study eye used | MP-1S, 17 stimuli within 12° radius  Full threshold^◊^, GIII, 1.27cd/m^2◊^  § Pupils dilated  § Sub-groups: early (n = 19 [/19]) and intermediate (n = 28 [/28]) AMD  § Analysis available within 12° radius (along horizontal and vertical meridians) only | Yes (calculated); within-study sub-group analysis possible | Yes | No |
|  |  |  |  |  | MP-1S, 17 stimuli within 12° radius  Full threshold^◊^, GIII, 0.0032cd/m^2◊^  § Pupils dilated  § Sub-groups: early (n = 19 [/19]) and intermediate (n = 28 [/28]) AMD  § Analysis available within 12° radius (along horizontal and vertical meridians) only | Yes (calculated); within-study sub-group analysis possible | Yes | No |
| **Fragiotta et al. 2017**(123) | Cross-sectional | Beckman Initiative:(111) intermediate | 22 [/22]; unspecified study eye used | - | MP-1, 68 stimuli within 10° radius^◊^  Full threshold, GIII, 1.27cd/m^2^  § Pupils dilated  § Practice exam included | - | - | No |
| **Fragiotta et al.**  **2022**(124) | Cohort | Beckman Initiative:(111) intermediate | 18 [/18] at final follow-up | 18 [/18] at baseline; unspecified study eye used | MAIA, 33 stimuli within 10° radius  Full threshold, GIII, 1.27cd/m^2^  § Pupils dilated  § Practice exam included  § Longitudinal follow-up over 12 months | No; within-study sub-group analysis possible | No; within-study sub-group analysis possible | No |
| **Gin et al. 2011**(125) | Case-control | ICGS:(109) early | 15 [/15]; better VA eye used, otherwise left eye used | 14 [/14]**^‡‖^**; better VA eye used, otherwise left eye used | Flicker perimetry/Medmont within 10° radius  ZEST fast-Bayesian with autoflicker^◊^, GIII, 3.2cd/m^2^  § Pupils dilated | Yes (calculated) | Yes (calculated) | No |
| **Goh et al. 2022**(126)  **[part of the LEAD study]**(127,128) | Cohort^†^ | Beckman Initiative:(111) intermediate | Unspecified [/134] at final follow-up | 280 [/140]**^‡‖^** at baseline; both eyes eligible from sham group | MAIA, 37 stimuli within 6° radius  Full threshold^◊^, GIII^◊^, 1.27cd/m^2◊^  § Pupils dilated  § Practice exam included  § Longitudinal follow-up over 36 months | No; within-study sub-group analysis possible | No; within-study sub-group analysis possible | No |
| **Grewal et al. 2021**(129) | Case-control | ICGS:(109) intermediate | 28 [/28]; unspecified eye used | 11 [/11]**^‖^**; unspecified eye used | Medmont DACP, 17 stimuli within 12° radius  Full threshold, GV, 0cd/m^2◊^  § Pupils dilated  § Background adaptation 40 minutes  § Stimuli were cyan 505nm and red 625nm  § Sub-groups: AMD with RPD (n = 11 [/11]) and AMD without RPD (n = 17 [/17]) | No | No | No |
| **Guymer et al. 2014**(104) | Case-control^†^ | Beckman Initiative:(111) intermediate | 46 [/23]; both eyes eligible at baseline | 46 [/23]**^‡‖^**; both eyes eligible at baseline | Flicker perimetry/Medmont within 10° radius  ZEST fast-Bayesian with autoflicker, GIII, 3.2cd/m^2^  § Pupils dilated  § Practice exam included  § Analysis available within 6° radius only  § Analysis available for ‘worst point defects’ only | No | No | No |
| **Guymer et al. 2021**(130) | Case-control | Beckman Initiative:(111) intermediate | 25 [/25]; better VA eye used, otherwise right eye used | 23 [/23]**^‡‖^**; better VA eye used, otherwise right eye used | MAIA, 37 stimuli within 6° radius  Full threshold^◊^, GIII^◊^, 1.27cd/m^2◊^  § Pupils dilated  § Background adaptation 30 minutes  § Sub-groups: AMD with RPD (n = 12 [/12]) and AMD without RPD (n = 13 [/13]) | Yes; within-study sub-group analysis possible | Yes | No |
| **Haimovici et al. 2002**(131) | Case-control | ICGS:(109) early + intermediate | 31 [/31]; random eye used | 11 [/11]**^‡^**_;_ random eye used | Humphrey Field Analyser, 76 stimuli within 30° radius  Full threshold, GV, 0cd/m^2◊^  § Pupils dilated  § Background adaptation 45 minutes  § Stimuli were blue 450nm (via filter)  § Analysis available for 3°, 3° and 9°, 9° co-ordinates only | Yes (calculated) | Yes (calculated) | No |
| **Higgins et al. 2020**(132) | Case-control | Beckman Initiative:(111) early + intermediate | 32 [/16]; both eyes eligible | 22 [/11]**^‡‖^**; both eyes eligible | MAIA, 37 stimuli within 5° radius  Full threshold, GIII, 1.27cd/m^2^  § Pupils dilated  § Analysis available for better [VA] and worse [VA] eyes | Yes (calculated) | Yes (calculated) | Yes |
| **Hsu et al. 2019**(133)  **[follow-up study from Cocce et al. 2018]**(118) | Cohort case-control | AREDS 4 step:(113) early + intermediate | 60 [/60]; better VA eye used, otherwise random eye used | 18 [/18]**^‡‖^**; better VA eye used, otherwise random eye used | MAIA, 37 stimuli within 5° radius  Full threshold, GIII, 1.27cd/m^2^  § Pupils dilated  § Longitudinal follow-up over 12 months  § Sub-groups: early (n = 25 [/25]) and intermediate (n = 35 [/35]) AMD eyes | Yes (calculated); within-study sub-group analyses possible | Yes; within-study sub-group analyses possible | No |
|  |  |  | 60 [/60] at final follow-up | 60 [/60]**^‡‖^** at baseline; better VA eye used, otherwise random eye used |  |  |  |  |
| **Huang et al. 2015**(134) | Cohort^†^ | AREDS 9 step:(122)­ early + intermediate | Unspecified [/unspecified] at final follow-up | Unspecified [/28]**^‡‖^** at baseline; unspecified study eye used from placebo group | MP-1, 41 stimuli within 5° radius  Full threshold, GIII^◊^, 1.27cd/m^2^  § Pupils dilated  § Background adaptation 10 minutes  § Practice exam included  § Longitudinal follow-up over 24 months | No; within-study sub-group analysis possible | No; within-study sub-group analysis possible | No |
| **Iwama et al. 2010**(135) | Cross-sectional | Modified ICGS:(109) ‘confluent soft drusen’ | 22 [/21]; both eyes eligible | - | MP-1, 57 stimuli within 5° radius  Full threshold, GIII, 1.27cd/m^2^  § Analysis available within 2° radius only | - | - | No |
| **Kar et al. 2020**(136) | Case-control | AREDS 9 step:(122) early + intermediate | Unspecified [/unspecified];  better VA eye used | Unspecified [/unspecified]**^‡‖^**;  better VA eye used | MP-1S, 17 stimuli within 12° radius  Full threshold, GIII, 1.27cd/m^2◊^  § Pupils dilated  § Sub-groups: early (n = unspecified [/unspecified]) and intermediate (n = unspecified [/unspecified]) AMD  § Analysis available within 3° radius (along horizontal and vertical meridians) only | - | - | No |
|  |  |  |  |  | MP-1S, 17 stimuli within 12° radius  Full threshold, GIII, 0.0032cd/m^2◊^  § Pupils dilated  § Sub-groups: early (n = unspecified [/unspecified]) and intermediate (n = unspecified [/unspecified]) AMD  § Analysis available within 3° radius (along horizontal and vertical meridians) only | - | - | No |
| **Leal et al. 2022**(137) | Cross-sectional | AREDS 4 step:(113) early + intermediate | 28 [/unspecified]; both eyes eligible | - | MAIA, 49 stimuli within 5° radius  Full threshold, GIII, 1.27cd/m^2^  § Pupils dilated  § Background adaptation 5 minutes  § Practice exam included  § Sub-groups: early (n = 11 [/unspecified]) and intermediate (n = 17 [/unspecified]) AMD  § Sub-groups: AMD with RPD (n = 6 [/unspecified]) and AMD without RPD (n = 22 [/unspecified]) | -; within-study sub-group analysis possible | - | No |
| **Luu et al. 2012**(138) | Cohort case-control | ICGS:(109) early | 39 [/39]; unspecified study eye used | 24 [/24]**^‡‖^**; unspecified study eye used | Flicker perimetry/Medmont within 10° radius  ZEST fast-Bayesian with autoflicker, GIII, 3.2cd/m^2^  § Practice exam included  § Pupils dilated  § Analysis within the 6° radius only  § Longitudinal follow-up over two years | Yes (calculated); within-study sub-group analysis possible | Yes; within-study sub-group analysis possible | No |
|  |  |  | 39 [/39] at final follow-up; unspecified study eye used | 39 [/39]**^‡‖^** at baseline; unspecified study eye used |  |  |  |  |
| **Luu et al. 2013**(139) | Cohort case-control | ICGS:(109) early | 266 [/266]; better VA eye used | 24 [/24]**^‡‖^**; better VA eye used | Static perimetry/Medmont 48 stimuli within 10° radius  ZEST fast-Bayesian, GIII, 3.2cd/m^2^  § Practice exam included  § Sub-groups: nine sub-groups based on features (n = 266 [/266]). See study for further details  § Longitudinal follow-up over one year | Yes (calculated); within-study sub-group analyses possible | Yes (calculated); within-study sub-group analyses possible | No |
|  |  |  | 129 [/129] at final follow-up | 266 [/266]**^‡‖^** at baseline; better VA eye used | Flicker perimetry/Medmont 48 stimuli within 10° radius  Luminance-pedestal with autoflicker, GIII, 3.2cd/m^2^  § Practice exam included  § Sub-groups: nine sub-groups based on features (n = 266 [/266]). See study for further details  § Longitudinal follow-up over one year |  |  | No |
| **Maynard et al.**  **2016**(140) | Case-control | AREDS 4 step:(113) early + intermediate | 18 [/18] mesopic; preferred eye used | 15 [/15]**^‡‖^**; preferred eye used | MP-1, 40 stimuli within 5° radius  Full threshold, GIII, 1.27cd/m^2^  § Pupils dilated | No | No | No |
|  |  |  | 15 [/15] low mesopic; preferred eye used |  | MP-1, 40 stimuli within 5° radius^◊^  Full threshold^◊^, GIII^◊^, 0.801cd/m^2^  § Pupils dilated  § 0.2 log unit neutral density filter used | No | No | No |
| **McGuinness et al. 2019**(141)  **[part of the LEAD study]**(127,128) | Cross-sectional^†^ | Beckman Initiative:(111) intermediate | 288 [/288]; better MP sensitivity eye used from pre-treatment group | - | MAIA, 37 stimuli within 5° radius  Full threshold^◊^, GIII^◊^, 1.27cd/m^2◊^  § Pupils dilated  § Practice exam included | - | - | Yes |
| **McGuinness et al. 2020**(142)  **[part of the LEAD study]**(127,128) | Cross-sectional^†^ | Beckman Initiative:(111) intermediate | 282 [/141]; both eyes eligible from sham group | - | MAIA, 37 stimuli within 5° radius  Full threshold^◊^, GIII^◊^, 1.27cd/m^2◊^  § Pupils dilated  § Practice exam included  § Analysis available for better [MP sensitivity] and worse [MP sensitivity] eyes | - | - | Yes |
| **Messenio et al.**  **2022**(143) | Case-control | ‘large drusen’ | 47 [/44]; both eyes eligible | 65 [/33]**^‡^**; both eyes eligible | MAIA, 6 stimuli within 6° eccentricity (superior macula 1.2° and 6° points only)  Full threshold, GIII, 1.27cd/m^2^ | No | No | No |
| **Midena et al. 2007**(144) | Cross-sectional | ‘early AMD’ | 13 [/13]; random eye used | - | MP-1, 61 stimuli within 5° radius  Full threshold, GIII, 1.27cd/m^2^  § Pupils dilated  § Background adaptation 15 minutes | - | - | No |
| **Montesano et al.**  **2020**(145) | Case-control | Beckman Initiative:(111) early + intermediate | 43 [/43]; worse VA eye used, otherwise right eye used | 56 [/56]**^‡‖^**; worse VA eye used, otherwise right eye used | S-MAIA, 44 stimuli within 10° radius  Full threshold, GIII, 1.27cd/m^2◊^  § Pupils dilated  § Practice exam included | Yes (calculated) | Yes (calculated) | No |
|  |  |  |  |  | S-MAIA, 44 stimuli within 10° radius  Full threshold, GIII, 0cd/m^2◊^  § Pupils dilated  § Practice exam included  § Background adaptation 30 minutes  § Stimuli were cyan 505nm and red 627nm | Yes (calculated) | Yes (calculated) | No |
| **Nassisi et al. 2021**(146) | Cross-sectional | Beckman Initiative:(111) early + intermediate | 30 [/30]; right eye used | - | S-MAIA, 52 stimuli within 9° radius  Full threshold, GIII, 0cd/m^2◊^  § Pupils dilated  § Practice exam included  § Background adaptation 30 minutes | - | - | No |
| **Nittala et al. 2019**(147) | Case-control | Beckman Initiative:(111) early + intermediate | 42 [/29]; both eyes eligible | 96 [/48]**^‡^**; both eyes eligible | MP-1S, 32 stimuli within 10° radius  Full threshold, GV, 1.27cd/m^2^  § Pupils dilated | Yes | Yes | No |
|  |  |  |  |  | MP-1S, 32 stimuli within 10° radius  Full threshold, GV, 0.0032cd/m^2◊^  § Pupils dilated  § Background adaptation 30 minutes  § Short wavelength filter (50% cut-off 502nm) and 1.0 or 2.0 log unit neutral density filter used |  |  |  |
| **Ogino et al. 2014**(148) | Case-control | ‘AMD with large drusen or DPED’ | 35 [/35]; unspecified study eye used | 20 [/20]**^‡‖^**; unspecified study eye used | MP-1, 55 stimuli within 10° radius  Full threshold, GIII, 1.27cd/m^2^  § Pupils dilated  § Analysis available within 8° radius only | Yes (calculated) | Yes (calculated) | No |
| **Ooto et al. 2015**(149) | Cross-sectional | AREDS 4 step:(113) early + intermediate | 51 [/39]; both eyes eligible | - | MP-1, 29 stimuli within 10° radius  Full threshold, GII, 1.27cd/m^2^  § Pupils dilated  § Sub-groups: AMD with RPD (n = 20 [/unspecified]) and AMD without RPD (n = 31 [/unspecified]) | -; within-study sub-group analysis possible | - | No |
| **Owsley et al. 2000**(150) | Case-control | Modified ICGS(109) and Wisconsin:(151) early | 80 [/80]; unspecified eye used | 12 [/12]**^‡^**; unspecified eye used | Humphrey Field Analyser, 51 stimuli within 19° radius  Full threshold, GV, 0cd/m^2◊^  § Pupils dilated  § Background adaptation 40 minutes  § Stimuli were cyan 500nm | Yes | Yes | No |
| **Parisi et al. 2007**(152) | Case-control | Wisconsin:(151) early | 30 [/30]; random eye used | 15 [/15]**^‡‖^**; random eye used | MP-1, 32 stimuli within 2.5° radius  Full threshold, GI, 1.27cd/m^2^  § Pupils dilated | Yes (calculated) | Yes (calculated) | No |
| **Pfau et al. 2018**(153)  **[controls from Pfau et al. 2017]**(154) | Case-control | Beckman Initiative:(111) intermediate, ‘soft drusen’ | 11 [/11]; better VA eye used | 20 [/16]**^‡^**; both eyes eligible | S-MAIA, 49 stimuli within 14° radius  Full threshold, GIII, 1.27cd/m^2◊^  § Pupils dilated  § Practice exam included | No; within-study sub-group analysis possible | Yes | No |
|  |  |  |  |  | S-MAIA, 49 stimuli within 14° radius  Full threshold, GIII, 0cd/m^2◊^  § Pupils dilated  § Background adaptation 30 minutes  § Stimuli were cyan 505nm and red 627nm | No; within-study sub-group analysis possible | Yes | No |
| **Phipps et al. 1999**(155) | Case-control | ‘high-risk drusen’ | 4 [/4]; unspecified study eye used | 4 [/4]**^‡^**; unspecified study eye used | Static perimetry/Medmont within 10° radius  Full threshold, GIII, 3.2cd/m^2^ | No | No | No |
|  |  |  |  |  | Flicker perimetry/Medmont within 10° radius  Autoflicker, GIII (approximately, 0.5°), 3.2cd/m^2^ | No | No | No |
| **Phipps et al. 2004**(156) | Case-control | Modified ICGS:(109) early AMD | 25 [/25]; better VA eye used, otherwise random eye used | 34 [/34]**^‡‖^**; better VA eye used, otherwise random eye used | Static perimetry/Medmont within 10° radius  ZEST fast-Bayesian, GIII, 3.2cd/m^2^  § Pupils dilated  § Practice exam included | No | No | No |
|  |  |  |  |  | Flicker perimetry/Medmont within 10° radius  ZEST fast-Bayesian with autoflicker, GIII, 3.2cd/m^2^  § Pupils dilated  § Practice exam included | No | No | No |
| **Pondorfer et al. 2019**(157) **[part of Pondorfer et al. 2020**(158) **study]** | Cross-sectional | Beckman Initiative:(111) early + intermediate | 52 [/52]; more advanced AMD eye used, otherwise right eye used | - | S-MAIA, 33 stimuli within 7° radius  Full threshold^◊^, GIII^◊^, 1.27cd/m^2◊^  § Pupils dilated  § Sub-groups: early (n = 10 [/10]) and intermediate (n = 42 [/42]) AMD | -; within-study sub-group analysis possible | - | Yes |
|  |  |  |  |  | S-MAIA, 33 stimuli within 7° radius  Full threshold^◊^, GIII^◊^, 0cd/m^2◊^  § Pupils dilated  § Background adaptation 30 minutes  § Stimuli were red 627nm  § Sub-groups: early (n = 10 [/10]) and intermediate (n = 42 [/42]) AMD | -; within-study sub-group analysis possible | - |  |
| **Pondorfer et al. 2020 [a]**(158) | Case-control | Beckman Initiative:(111) early + intermediate | 78 [/78]; unspecified study eye used | 22 [/22]**^‡‖^**; unspecified study eye used | S-MAIA, 33 stimuli within 7° radius  Full threshold^◊^, GIII^◊^, 1.27cd/m^2◊^  § Pupils dilated  § Sub-groups: early (n = 16 [/16]) and intermediate (n = 62 [/62]) AMD | Yes (calculated); within-study sub-group analysis possible | Yes | No |
|  |  |  |  |  | S-MAIA, 33 stimuli within 7° radius  Full threshold^◊^, GIII^◊^, 0cd/m^2◊^  § Pupils dilated  § Background adaptation 30 minutes  § Stimuli were red 627nm  § Sub-groups: early (n = 16 [/16]) and intermediate (n = 62 [/62]) AMD | Yes (calculated); within-study sub-group analysis possible | Yes |  |
| **Pondorfer et al. 2020 [b]**(159) **[part of Pondorfer et al. 2020**(158) **study]** | Case-control | Beckman Initiative:(111) intermediate | 38 [/38]; better VA eye used, otherwise right eye used | 24 [/24]**^‡‖^**; better VA eye used, otherwise right eye used | S-MAIA, 33 stimuli within 7° radius  Full threshold^◊^, GIII^◊^, 1.27cd/m^2◊^  § Pupils dilated | Yes | Yes | No |
|  |  |  |  |  | S-MAIA, 33 stimuli within 7° radius  Full threshold^◊^, GIII^◊^, 0cd/m^2◊^  § Pupils dilated  § Background adaptation 30 minutes  § Stimuli were red 627nm | Yes | Yes |  |
| **Prea et al. 2021**(105) | Cross-sectional^†^ | Beckman Initiative**^◊^**:(111) intermediate | 118 [/59]; both eyes eligible | - | iPad; Melbourne Rapid Fields App, 33 stimuli within 9.5° radius  Full threshold, stimuli size scaled with eccentricity, 5cd/m^2^ | - | - | No |
|  |  |  |  |  | MAIA, 37 stimuli within 6° radius^◊^  Full threshold, GIII, 1.27cd/m^2◊^ |  |  |  |
| **Querques et al. 2021**(106) | Cross-sectional^†^ | Beckman Initiative:(111) early + intermediate (with RPD) | 20 [/20] at baseline; worse VA eye used, otherwise right eye used | - | MP-1S, unspecified number of stimuli within unspecified custom area  Full threshold, GV, 0.0032cd/m^2^  § Pupils dilated  § Background adaptation 30 minutes  § Practice exam included | - | - | No |
| **Roh et al. 2019**(160)  **[participants from one other unspecified study]** | Case-control | AREDS 4 step:(113) early + intermediate | 87 [/unspecified]; both eyes eligible | 46 [/unspecified]**^‡^**; both eyes eligible | MAIA, 37 stimuli within 5° radius  Full threshold, GIII, 1.27cd/m^2^  § Pupils dilated  § Practice exam included  § Sub-groups: early (n = 16 [/unspecified]) and intermediate (n = 71 [/unspecified]) AMD | Yes (calculated); within-study sub-group analysis possible | Yes | No |
| **Sassmannshausen et al. 2018**(161) | Cross-sectional | Beckman Initiative:(111) intermediate | 35 [/32]; both eyes eligible | - | MP-1S, 56 stimuli within 10° radius  Full threshold, GIII, 1.27cd/m^2^  § Pupils dilated | - | - | No |
|  |  |  |  |  | MP-1S, 56 stimuli within 10° radius  Full threshold, GV, 0.0032cd/m^2^  § Pupils dilated  § Background adaptation 30 minutes  § 2.0 log unit neutral density filter used | - | - | No |
| **Sassmannshausen et al. 2020**(162) **[part of Sassmannshausen et al. 2021**(163) **study]** | Cohort^†^ | Beckman Initiative:(111) intermediate | 11 [/7] at final follow-up; both eyes eligible | 30 [/25]**^‡‖^** at baseline; both eyes eligible | MP-1S, 56 stimuli within 10° radius  Full threshold, GIII, 1.27cd/m^2^  § Pupils dilated  § Longitudinal follow-up over 36 months | No; within-study sub-group analysis possible | No; within-study sub-group analysis possible | No |
|  |  |  |  |  | MP-1S, 56 stimuli within 10° radius  Full threshold, GV, 0.0032cd/m^2^  § Pupils dilated  § Background adaptation 20 minutes  § 0, 1.0, or 2.0 log unit neutral density filter used  § Longitudinal follow-up over 36 months | No; within-study sub-group analysis possible | No; within-study sub-group analysis possible |  |
| **Sassmannshausen et al. 2021**(163) | Cohort case-control | Beckman Initiative:(111) intermediate | 59 [/54]; both eyes eligible | 27 [/27]**^‡‖^**; both eyes eligible | MP-1S, 56 stimuli within 10° radius  Full threshold, GIII, 1.27cd/m^2^  § Pupils dilated  § Longitudinal follow-up over 36 months | No; within-study sub-group analysis possible | No; within-study sub-group analysis possible | No |
|  |  |  | 14 [/unspecified] at final follow-up | 59 [/54]**^‡‖^** at baseline; both eyes eligible | MP-1S, 56 stimuli within 10° radius  Full threshold, GV, 0.0032cd/m^2^  § Pupils dilated  § Background adaptation 30 minutes  § 0, 1.0, or 2.0 log unit neutral density filter used  § Longitudinal follow-up over 36 months |  |  |  |
| **Steinberg et al. 2016**(164) | Cross-sectional^†^ | Beckman Initiative:(111) early + intermediate | 20 [/18]; both eyes eligible | - | MP-1S, 56 stimuli within 10° radius^◊^  Full threshold, GIII, 1.27cd/m^2^  § Pupils dilated | - | - | No |
|  |  |  |  |  | MP-1S, 56 stimuli within 10° radius^◊^  Full threshold, GV, 0.0032cd/m^2^  § Pupils dilated  § Background adaptation 30 minutes  § 1.0 log unit neutral density filter used | - | - |  |
| **Steinmetz et al. 1993**(165) | Case-control | ‘Age-related maculopathy’ | 12 [/12]; better VA eye used | Unspecified [/unspecified]**^‡‖^** | Humphrey field analyser, 76 stimuli within 30° radius  Full threshold, GV, 0cd/m^2^  § Pupils dilated  § Background adaptation 45 minutes  § Stimuli were blue 450nm | No | No | No |
| **Tan et al. 2018**(166) | Case-control | Beckman Initiative:(111) intermediate | 37 [/37]; better VA eye used, otherwise right eye used | 29 [/29]**^‡‖^**; better VA eye used, otherwise right eye used | Medmont DACP, 28 stimuli within 24° radius  Full threshold, GV, 0cd/m^2◊^  § Pupils dilated  § Background adaptation 30 minutes  § Stimuli were cyan 505nm and red 625nm  § Sub-groups: AMD with RPD (n = 17 [/17]) and AMD without RPD (n = 20 [/20]) | No; within-study sub-group analysis possible | No | No |
| **Taylor et al. 2019**(107) | Cross-sectional^†^ | Beckman Initiative:(111) early + intermediate | Unspecified [/7]; both eyes eligible | - (early and no AMD group not separated) | MAIA, 37 stimuli within 5° radius  Full threshold, GIII, 1.27cd/m^2^  § Pupils dilated | - | - | Yes |
| **Tepelus et al. 2017**(167) | Cross-sectional^†^ | ‘intermediate non-neovascular AMD’ | 37 [/25]; both eyes eligible | - | MP-3, 33 stimuli within 10° radius  Full threshold, GIII, 1.27 cd/m^2◊^  § Pupils dilated  § Analysis available within 8° radius only | - | - | No |
| **Thompson et al. 2018**(168)  **[part of Cocce et al. 2018 study]**(118) | Case-control | AREDS 4 step:(113) early + intermediate | 80 [/80]; better VA eye used, otherwise random eye used | 21 [/21]**^‡‖^**; better VA eye used, otherwise random eye used | MAIA, 37 stimuli within 5° radius  Full threshold, GIII, 1.27cd/m^2^  § Pupils dilated  § Sub-groups: early (n = 33 [/33]) and intermediate (n = 47 [/47]) AMD | No; within-study sub-group analysis possible | No | Yes |
| **Uddin et al. 2020**(169) | Case-control | Beckman Initiative**^◊^**:(111) intermediate | 9 [/9]; unspecified eye used | 3 [/3]; unspecified eye used | Medmont DACP, 14 stimuli within 18° radius  Full threshold, GV, 0cd/m^2◊^  § Pupils dilated  § Background adaptation 30 minutes  § Stimuli were cyan 505nm and red 625nm  § Sub-groups: AMD with RPD (n = 5 [/5]) and AMD without RPD (n = 4 [/4]) | No | No | No |
| **Vujosevic et al. 2011**(170) | Case-control | AREDS 4 step:(113) early + intermediate | 319 [/200]; both eyes eligible | Unspecified [/200]**^‡^**; both eyes eligible | MAIA, 61 stimuli within 10° radius  Full threshold, GIII, 1.27cd/m^2^  § Sub-groups: early (n = 164 [/unspecified]) and intermediate (n = 155 [/unspecified]) AMD | Yes (calculated) | Yes | No |
| **Vujosevic et al. 2017**(171)  **[follow-up study from Vujosevic et al. 2011]**(170) | Cohort | AREDS 4 step:(113) early + intermediate | 29 [/16]**^‡^**; both eyes eligible | - | MAIA, 61 stimuli within 10° radius  Full threshold, GIII, 1.27cd/m^2^  § Background adaptation 10 minutes  § Longitudinal follow-up over 72 months  § Sub-groups: early (n = 17 [/9]) and intermediate (n = 12 [/7]) AMD | No; within-study sub-group analyses possible | No; within-study sub-group analyses possible | No |
| **Weingessel et al. 2009**(172) | Case-control | AREDS 4 step:(113) intermediate | 5 [/5]; random eye used | 15 [/15]**^‡^**; random eye used | MP-1, 41 stimuli within 5° radius  Full threshold, GIII, 1.27cd/m^2^  § Background adaptation 5 minutes | Yes | Yes | No |
| **Welker et al.**  **2018**(173) | Case-control | Beckman Initiative:(111) intermediate | 23 [/23]; right eye used | 24 [/24]**^‡‖^**; right eye used | S-MAIA, 33 stimuli within 7° radius  Full threshold, GIII,^◊^ 1.27cd/m^2^  § Pupils dilated | Yes | Yes | No |
|  |  |  |  |  | S-MAIA, 33 stimuli within 7° radius  Full threshold, GIII,^◊^ 0cd/m^2^  § Pupils dilated  § Background adaptation 30 minutes  § Stimuli were red 627nm | Yes | Yes |  |
| **Wightman et al 2019**(174) **[part of the LEAD study]**(127,128) | Cohort | Beckman Initiative:(111) intermediate (‘non-progressors’) | 14 [/14]**^‡^**; unspecified eye used | - | MAIA, 37 stimuli within 6° radius  Full threshold, GIII, 1.27cd/m^2^  § Pupils dilated  § Practice exam included  § Longitudinal follow-up over 12 months | No; within-study sub-group analysis possible | No; within-study sub-group analysis possible | No |
| **Wu et al. 2013**(175)  **[participants from another unspecified study]** | Case-control | Beckman Initiative:(111) early + intermediate | 30 [/30]; better VA eye used | 14 [/14]**^‡‖^**; better VA eye used | MAIA, 37 stimuli within 6° radius  Full threshold, GIII, 1.27cd/m^2^  § Pupils dilated  § Practice exam included | No | No | No |
| **Wu et al. 2014 [a]**(176) | Cross-sectional | ‘intermediate AMD’ | 24 [/22]; both eyes eligible | - | MAIA, 37 stimuli within 6° radius  Full threshold, GIII, 1.27cd/m^2^  § Pupils dilated  § Practice exam included | - | - | No |
| **Wu et al. 2014 [b]**(177) | Case-control | Beckman Initiative:(111) early + intermediate | 179 [/179]; better VA eye used | 26 [/26]**^‡‖^**; better VA eye used | MAIA, 37 stimuli within 6° radius  Full threshold, GIII, 1.27cd/m^2^  § Pupils dilated  § Practice exam included  § Sub-groups: early (n = 16 [/16]) and intermediate (n = 163 [/163]) AMD  § Analysis available within 1° radius only | No; within-study sub-group analysis possible | No | No |
| **Wu et al. 2014 [c]**(178) | Case-control | Beckman Initiative:(111) intermediate | 75 [/75]; random eye used | 25 [/25]**^‡‖^**; random eye used | MAIA, 37 stimuli within 6° radius  Full threshold, GIII, 1.27cd/m^2^  § Pupils dilated  § Practice exam included  § Analysis available within 2.33° radius (along horizontal meridian) only | No | No | No |
| **Wu et al. 2014 [d]**(179) | Case-control | Beckman Initiative:(111) intermediate | 60 [/60]; better VA eye used | 22 [/22]**^‡‖^**; better VA eye used | MAIA, 37 stimuli within 6° radius  Full threshold, GIII, 1.27cd/m^2^  § Pupils dilated  § Practice exam included | Yes | Yes | No |
| **Wu et al. 2015 [a]**(180) | Cross-sectional | Beckman Initiative:(111) intermediate | 60 [/30]; both eyes eligible | - | iPad; PsyPad App, 5 stimuli within 1° radius  Full threshold, GIII, 1.27cd/m^2^  § Pupils dilated  § Practice exam included | - | - | No |
|  |  |  |  |  | MAIA, 37 stimuli within 6° radius  Full threshold, GIII, 1.27cd/m^2^  § Pupils dilated  § Practice exam included | - | - |  |
| **Wu et al. 2015 [b]**(181) | Cohort case-control | Beckman Initiative:(111) intermediate | 49 [/49]; better VA eye used | 10 [/10]**^‡‖^**; better VA eye used | MAIA, 37 stimuli within 6° radius  Full threshold, GIII, 1.27cd/m^2^  § Pupils dilated  § Practice exam included  § Longitudinal follow-up over 12 months | Yes (calculated); within-study sub-group analysis possible | No; within-study sub-group analysis possible | No |
|  |  |  | 49 [/49] at final follow-up | 49 [/49]**^‡^** at baseline; better VA eye used |  |  |  |  |
| **Wu et al. 2015 [c]**(182) | Cross-sectional | Beckman Initiative:(111) intermediate | 120 [/60]; both eyes eligible | - | MAIA, 37 stimuli within 6° radius  Full threshold, GIII, 1.27cd/m^2^  § Pupils dilated  § Practice exam included  § Sub-groups: AMD with RPD (n = 39 [/unspecified]) and AMD without RPD (n = 81 /unspecified]) eyes | -; within-study sub-group analysis possible | - | No |
| **Wu et al. 2016 [a]**(183)  **[follow-up study from Wu et al. 2015]**(181) | Cohort | Beckman Initiative:(111) intermediate | 39 [/39] at final follow-up | 41 [/41]**^‡‖^** at baseline; better VA eye used | MAIA, 37 stimuli within 6° radius  Full threshold, GIII, 1.27cd/m^2^  § Pupils dilated  § Practice exam included  § Longitudinal follow-up over 12 months | No; within-study sub-group analysis possible | No; within-study sub-group analysis possible | No |
| **Wu et al. 2016 [b]**(184) | Cross-sectional | Beckman Initiative:(111) intermediate | 100 [/100]; better VA eye used | - | MAIA, 37 stimuli within 6° radius  Full threshold, GIII, 1.27cd/m^2^  § Pupils dilated  § Practice exam included  § Analysis available for better [VA] and worse [VA] eyes  § Analysis available within 1° radius only | - | - | Yes |
| **Wu et al. 2019**(185)  **[part of the LEAD study]**(127,128) | Cohort | Beckman Initiative:(111) intermediate | 268 [/unspecified] at final follow-up | 286 [/143]**^‡‖^** at baseline; both eyes eligible from sham group | MAIA, 37 stimuli within 6° radius^◊^  Full threshold, GIII^◊^, 1.27cd/m^2◊^  § Pupils dilated  § Practice exam included  § Longitudinal follow-up over 36 months | No; within-study sub-group analysis possible | No; within-study sub-group analysis possible | No |
| **Wu et al. 2020**(186)  **[part of the LEAD study]**(127,128) | Cohort | Wisconsin ARM grading system:(151) intermediate | Unspecified [/134] at final follow-up | 279 [/140]**^‡‖^** at baseline; both eyes eligible from sham group | MAIA, 37 stimuli within 6° radius^◊^  Full threshold, GIII^◊^, 1.27cd/m^2◊^  § Pupils dilated  § Practice exam included  § Longitudinal follow-up over 36 months | No; within-study sub-group analysis possible | No; within-study sub-group analysis possible | No |
| **Wu et al. 2021**(187)  **[part of the LEAD study]**(127,128) | Cohort | Beckman Initiative:(111) intermediate | Unspecified [/128] at final follow-up | Unspecified [/142]**^‡‖^** at baseline; both eyes eligible from sham group | MAIA, 37 stimuli within 6° radius  Full threshold^◊^, GIII^◊^, 1.27cd/m^2◊^  § Pupils dilated^◊^  § Practice exam included^◊^  § Longitudinal follow-up over 36 months | No; within-study sub-group analysis possible | No; within-study sub-group analysis possible | No |
| **Zhang et al. 2022**(188) | Case-control | AREDS 9 step:(122) early + intermediate | 12 [/12]; unspecified eye used | 6 [/6]**^‡^** mesopic; unspecified eye used | MP-1S, 68 stimuli within 10.4° radius  Full threshold, GIII, 1.27cd/m^2◊^  § Pupils dilated  § Practice exam included  § Background adaptation 15 minutes  § Sub-groups: AMD with RPD (n = 12 [/12]) and AMD without RPD (n = 12 [/12]) | No | No | No |
|  |  |  |  | 9 [/9]**^‡^** scotopic; unspecified eye used | MP-1S, 68 stimuli within 10.4° radius  Full threshold, GIII, 0.0032cd/m^2◊^  § Pupils dilated  § Background adaptation 40 minutes  § Sub-groups: AMD with RPD (n = 12 [/12]) and AMD without RPD (n = 12 [/12]) | No | No |  |

**Supplementary Table 5. Funding and conflict of interest statements (*verbatim*).**

| **Study** | **Funding** | **Conflict of interest** |
| --- | --- | --- |
| **Adams et al. 2018**(110) | *Supported by The National Health and Medical Research Council), Principal Research Fellowship (GNT1103013) and Early Career Fellowship (#1104985, ZW). The Centre for Eye Research Australia (CERA) receives Operational Infrastructure Support from the Victorian Government.* | *Disclosure: M. Adams, None; C.Y.D. Ho, None; E. Baglin, None; P. Sharanan, None; Z. Wu, None, D. J. Lawson, None, C.D. Luu, None, A. Turpin, None, A.M. McKendrick, None, R.H. Guymer, Advisory Board member for Novartis, Bayer, Roche/Genentech (C)* |
| **Broadhead et al. 2017**(112) | *No author has any conflict of interests to declare, and there was no funding associated with this study. One previous researcher associated with this study, who had no patient contact, was previously required to withdraw his involvement from this study due to a conflict of interests.* | *No author has any conflict of interests to declare, and there was no funding associated with this study. One previous researcher associated with this study, who had no patient contact, was previously required to withdraw his involvement from this study due to a conflict of interests.* |
| **Broadhead et al. 2020**(114) | *No authors have any conflicts of interest to declare, and there was no funding associated with this study. One previous researcher associated with this study who had no participant contact, was previously required to withdraw his involvement from the study due to a conflict of interest.* | *No authors have any conflicts of interest to declare, and there was no funding associated with this study. One previous researcher associated with this study who had no participant contact, was previously required to withdraw his involvement from the study due to a conflict of interest.* |
| **Chandramohan et al. 2016**(115) | *Eleonora Lad was supported by the NEI Clinical Scientist Development award NIH/NEI 5K12 EY016333-08.* | - |
| **Chen et al. 2004**(116) | - | - |
| **Clemens et al. 2015**(117) | - | - |
| **Cocce et al. 2018**(118) | *THIS WORK WAS SUPPORTED BY THE NATIONAL INSTITUTES OF HEALTH/NATIONAL EYE INSTITUTE, Bethesda, MD (K23EY026988), Research to Prevent Blindness, New York, NY, and, through Duke University, and industry support from Hoffmann- La Roche, Basel, Switzerland.* | *Ulrich F.O. Luhmann is an employee of F. Hoffmann-La Roche Ltd; Cynthia A. Toth receives financial support from Genetech and holds a patent with Alcon Laboratories; Scott W. Cousins has financial relationships with B&L (consulting and grant for a trial), Stealth BioTherapeutics (consulting and grant for trial), Alimera Sciences (consulting), PanOptica (consulting and equity), TheraKine (consulting/ directorship), and Eyedesis (founder, equity and consulting); Eleonora M. Lad receives financial support from Hoffman La Roche. The following authors have no financial disclosures: Kimberly J. Cocce, Sandra S. Stinnett, Lejla Vajzovic, Anupama Horne, and Stefanie G. Schuman. The authors attest that they meet the current ICMJE criteria for authorship.* |
| **Corvi et al. 2019**(119) | - | - |
| **Dinc et al. 2008**(120) | *None of the authors has a proprietary interest and they received no financial support.* | *None of the authors has a proprietary interest and they received no financial support.* |
| **Dow et al. 2016**(103) | *This study was funded by TA-Sciences, New York.* | *CB Harley is the lead investigator of a use patent on TA-65, was a paid scientific consultant to TA-Sciences, and owns stock in two companies related to this publication (Geron Corporation, and Telomere Diagnostics Inc). The authors report no other conflicts of interest in this work.* |
| **Echols et al. 2020**(121) | *Supported by the National Institutes of Health, Bethesda, Maryland (grant nos.: R01AG04212, R01EY029595, R01EY027948, R01EY024378, and P30EY03039); the Center for Clinical and Translational Science (University of Alabama at Birmingham, grant no.: TL1TR001418); EyeSight Foundation of Alabama, Birmingham AL; Dorsett Davis Discovery Fund, Birmingham AL; Alfreda J. Schueler Trust, Chicago IL; Research to Prevent Blindness Inc., New York, New York; and the Macula Foundation, New York, NY.* | *The author(s) have made the following disclosure(s): L.C.: Financial support – Heidelberg Engineering, Roche K.R.S.: Equity owner – MacRegen C.O.: Inventor – AdaptDx C.A.C.: Consultant – Genentech/Hoffman LaRoche; Financial support – Heidelberg Engineering, Genentech/Hoffman LaRoche; Equity owner – MacRegen* |
| **Fragiotta et al. 2017**(123) | - | *The authors declare that they have no competing interests.* |
| **Fragiotta et al. 2022**(124) | *Supported by the Italian Ministry of Health and Fondazione Roma. SF received the support of Projects to Start Research- Type 2 provided by Sapienza University (2021; protocol number AR22117A81528669).* | *S. Fragiotta, None; E. Costanzo, None; P. Viggiano, None; D. De Geronimo, None; G. Scuderi, None; M. Varano, Allergan (F), Novartis (F), Bayer (F), Sifi (F); M.Parravano, Allergan (F), Novartis (F), Bayer (F), Zeiss (F), Omikron (F), Alfaintes (F), Sifi (F).*  *The funders had no role in the study design, data collection and analysis, decision to publish, or preparation of the manuscript.* |
| **Gin et al. 2011**(125) | *Supported by a National Health and Medical Research Practitioner Fellowship (RHG), the Macular Vision Loss Support Society of Australia, Operational Infrastructure Support from the Victorian Government (CERA), and NHMRC Centre for Clinical Research Excellence Award 529923 (CERA).* | *Disclosure: T.J. Gin, None; C.D. Luu, None; R.H. Guymer, None* |
| **Goh et al. 2022**(189) | *Supported by the National Health and Medical Research Council of Australia (project grant no.: APP1027624 [R.H.G.] and fellowship grant*  *no.: GNT1103013 [R.H.G.]) and grants from the Macular Disease Foundation Australia (Z.W. and R.H.G.) and the BrightFocus Foundation (grant no.: M2019073 [Z.W.]). Centre for Eye Research Australia receives operational infrastructure support from the Victorian Government. The sponsors or funding organizations had no role in the design or conduct of this research.* | *X.H.: Grant – H&L Hecht Trust and Centre for Eye Research Australia Innovation Fund. M.J.: Grant – H&L Hecht Trust and Centre for Eye Research Australia Innovation Fund. P.v.W.: Grant – H&L Hecht Trust and Centre for Eye Research Australia Innovation Fund; Personal Fees – Bayer Australia. R.H.G.: Personal Fees e Roche/Genentech, Bayer, Novartis, and Apellis. The other authors have no proprietary or commercial interest in any materials discussed in this article.* |
| **Grewal et al. 2021**(129) | *The research was funded by Fight for Sight (Ref 1905b) and supported by the NIHR Biomedical Research Centre at Moorfields Eye Hospital NHS Foundation Trust and UCL Institute of Ophthalmology and the NIHR Moorfields Clinical Research Facility. The views expressed are those of the author(s) and not necessarily those of the NHS, the NIHR or the Department of Health.* | *The authors declare no competing interests.* |
| **Guymer et al. 2014**(104) | *This work was supported by a grant to the Centre for Eye Research Australia, from the Victorian State Government under the Victoria’s Science Agenda Investment Fund and from Ellex R&D Pty Ltd, Adelaide. RHG receives a National Health and Medical Research Council (NHMRC) practitioner fellowship (#529905). CERA is supported by a NHMRC Centre for Clinical Research Excellence #529923 award and receives operational infrastructure from the Victorian Government. The funding organizations had no role in the design or conduct of this research.* | *Ellex provided the laser and contributed some funds to CERA to support the pilot study. Ellex’s participation related to protocol development and advice regarding the evaluation of the visual function results. AV provides consultancy to Medmont Pty Ltd and receives research support from the company.* |
| **Guymer et al. 2021**(130) | *Supported byRyan Initiative for MacularResearch, Australia Awards Scholarship (RST), National Health and Medical Research Council (NHMRC) Fellowship (GNT1103013, RHG). The Centre for Eye Research Australia (CERA) receives Operational Infrastructure Support from the Victorian Government.* | *R.H. Guymer, None; R.S. Tan, None; C.D. Luu, None* |
| **Haimovici et al. 2002**(131) | - | - |
| **Higgins et al. 2020**(132) | *This study was funded as part of an unrestricted investigator-initiated research grant from Roche Products Ltd, UK, (https://www.roche.co.uk/) awarded to DPC. The funders had no role in study design, data collection and analysis, decision to publish, or preparation of the manuscript.* | *DPC is a consultant for Centervue and reports unrestricted grants from Roche UK, Santen UK, and Allergan UK and speaker fees from THEA, Bayer, Santen, and Allergan which are outside the submitted work. This study was funded as part of an unrestricted investigator-initiated research grant from Roche Products Ltd, UK, (https://www.roche.co.uk/) awarded to DPC. The funders had no role in study design, data collection and analysis, decision to publish, or preparation of the manuscript. This does not alter our adherence to PLOS ONE policies on sharing data and materials. The remaining authors report no competing interests.* |
| **Hsu et al 2019**(133) | *Supported by the National Eye Institute, National Institutes of Health, Bethesda, Maryland (grant no.: K23EY026988); Research to Prevent Blindness, Inc, New York, New York; the Knights Templar Eye Foundation (L.V.); the Second Sight Foundation (L.V.); Duke University, Durham, North Carolina (Private Diagnostic Clinic Enable Award to L.V.); F. Hoffmann − La Roche Ltd., Basel, Switzerland (through Duke University).* | *The author(s) have made the following disclosure(s): U.F.O.L.: Employee − F. Hoffmann − La Roche Ltd. (Basel, Switzerland). Lejla Vajzovic: Financial support − Janssen Pharmaceutical, Roche, DORC, Alcon, Genentech. C.A.T.: Consultant and Financial support − Emmes; Royalties − Alcon. S.W.C.: Consultant − Bausch & Lomb, Stealth BioTherapeutics, Alimera Sciences, PanOptica, TheraKine, Eyedesis; Financial support − B&L, Stealth BioTherapeutics; Equity owner − PanOptica, Eyedesis; Board member − TheraKine; Founder − Eyedesis. E.M.L.: Consultant e Stealth BioTherapeutics; Financial support − F. Hoffman − La Roche (Basel, Switzerland), Apellis, Novartis.* |
| **Huang et al. 2015**(134) | *Supported by the Chinese National Natural Science Foundation (grant 81273063).* | *None.* |
| **Iwama et al. 2010**(135) | - | - |
| **Kar et al. 2020**(136) | Supported by National Institutes of Health Grants (R01AG04212, R01EY029595, and R01EY027948); EyeSight Foundation of Alabama; Dorsett Davis Discovery Fund; Alfreda J. Schueler Trust; Research to Prevent Blindness, Inc.; and Heidelberg Engineering. | Disclosure: D. Kar, None; M.E. Clark, None; T.A. Swain, None; G. McGwin Jr., None; J.N. Crosson, None; C. Owsley, (P); K.R. Sloan, MacRegen (I); C.A. Curcio, Heidelberg Engineering (F), Genentech/Hoffman LaRoche (F), MacRegen (I) |
| **Leal et al. 2022**(137) | *The author(s) received no financial support for the research, authorship and/or publication of this article.* | *The author(s) declared the following potential conflicts of interest with respect to the research, authorship and/or publication of this article: Morales Marco: Personal Financial Interest (CenterVue) and consultant.* |
| **Luu et al. 2012**(138) | *This research was supported by grant 350224 RHG/AJV from the National Health and Medical Research Council, grant ARC-LP0211474 from the Australian Research Council Linkage Project, practitioner fellowship 529905 from the National Health and Medical Research Council (Dr Guymer), a Royal Victorian Eye and Ear Hospital Wagstaff Fellowship (Dr Robman), the Royal Victorian Eye and Ear Hospital Research Committee, and the Macular Vision Loss Support Society of Australia. The Centre for Eye Research Australia receives operational infrastructure support from the Victorian government and is supported by Excellence Award 529923 from the National Health and Medical Research Council Centre for Clinical Research.* | *Dr Vingrys is a consultant to Medmont Pty Ltd and receives research support from the company.* |
| **Luu et al. 2013**(139) | *Supported by the National Health and Medical Research Council (NH and MRC) Project Grant 350224, Australian Research Council Linkage Project (ARC-LP0211474), Macular Degeneration Foundation Research Grant, NH and MRC practitioner fellowship (RHG, #529905). CERA receives Operational Infrastructure Support from the Victorian Government and is supported by a NHMRC Centre for Clinical Research Excellence Award (#529923).* | *Disclosure: C.D. Luu, None; P.N. Dimitrov, None; Z. Wu, None; L.N. Ayton, None; G. Makeyeva, None; K.-Z. Aung, None; M. Varsamidis, None; L. Robman, None; A.J. Vingrys, Medmont International Pty Ltd. (F, C); R.H. Guymer, None* |
| **Maynard et al. 2016**(140) | *This project was supported by Australian Research Council Discovery Projects ARC-DP140100333 and an IHBI Vision and Eye Program Grant.* | *The sponsor or funding organization had no role in the design or conduct of this research.* |
| **McGuinness et al. 2019**(141) | *The Laser intervention in Early stages of Agerelated macular Degeneration (LEAD) Study was supported by grants from the National Health & Medical Research Council (NHMRC) of Australia Project grant (1027624, RG and CL), the BUPA Health Foundation (Australia), and Ellex Medical Lasers, Ltd. (Australia) who also provided the investigational devices. RG is funded by a NHMRC Principal Research Fellowship 1103013. ZW received funding from NHMRC (Fellowship Grant 1104985). CERA received operational infrastructure support from the Victorian government and the NHMRC (Centre for Research Excellence grant 529923). FC received funding from NHMRC (Early Career Fellowship 1054712 and Career Development Fellowship 1142962).* | *Disclosure: M.B. McGuinness, None; R.P. Finger, None; Z. Wu, None; C.D. Luu, None; F.K. Chen, None; J.J. Arnold, None; U. Chakravarthy, None; W.J. Heriot, None; J. Runciman, Ellex Medical Lasers Ltd. (C); R.H. Guymer, None* |
| **McGuinness et al. 2020**(142) | *The author(s) have made the following disclosure(s): R.P.F.: Financial support − Novartis, Bayer, Roche/Genentech, Allergan, Alimera, Böhringer-Ingelheim, Ellex, Ophthea, Inositec, Santhera, CentreVue J.J.A.: Consultant − Bayer, Alcon; Board membership – Allergan, Alcon, Bayer, Novartis; Lecturer − Allergan, Bayer R.H.G.: Board membership − Novartis, Roche*  *The Laser Intervention in Early Stages of Age Related Macular Degeneration (LEAD) Study was funded by the National Health and Medical Research Council of Australia (project grant no.: 1027624 [R.H.G. and C.D.L.); the BUPA Health Foundation, Australia; and Ellex Medical Lasers, Ltd., who also provided funds and the investigational devices. Supported by the National Health and Medical Research Council (principal research fellowship no.: 1103013 [R.H.G.]; fellowship grant no.: 1104985 [Z.W.]; early career fellowship no.: 1054712 [F.K.C.]; and career development fellowship no.: 1142962 [F.K.C.]); and the Victorian government and the National Health and Medical Research Council (Centre for Research Excellence grant 529923 [Centre for Eye Research Australia]).* | *The funding organizations had no role in study design, data collection and analysis, decision to publish, or preparation of the manuscript.* |
| **Messenio et al. 2022**(143) | - | - |
| **Midena et al. 2007**(144) | - | *None.* |
| **Montesano et al. 2020**(145) | *The NISA study was funded by the College of Optometrists, Macular Society, RNIB, Diabetes UK, and the Thomas Pocklington Trust. DPC and GM received funding from the Innovative Medicines Initiative 2 Joint Undertaking under grant 116076 (Macustar). This joint undertaking receives support from the European Union’s Horizon 2020 research and innovation program and European Federation of Pharmaceutical Industries and Associations (EFPIA).* | *G. Montesano, CenterVue, SpA (C); G. Ometto, (N); B.E. Higgins, (N); C. Iester, (N); K. Balaskas, (N); A. Tufail, (N); U. Chakravarthy, (N); R.E. Hogg, (N); D.P. Crabb, CenterVue, SpA (C)* |
| **Nassisi et al. 2021**(146) | *Dr Sadda: Allergan (consultant, financial support), Carl Zeiss Meditec (financial support), CenterVue (consultant), Genentech (consultant, financial support), Heidelberg Engineering (consultant), Iconic (consultant), NightstarX (consultant), Novartis (consultant), Optos (consultant, financial support), Thrombogenics (consultant), Topcon (consultant).* | *DRS NASSISI, TEPELUS, AND Corradetti declare no conflict of interests.* |
| **Nittala et al. 2019**(147) | *Supported by R01 EY023164 (DS), R01 EY 09076 (DGB), the Department of Ophthalmology at the Perelman School of Medicine, University of Pennsylvania, Philadelphia, Pennsylvania (DS), the Foundation Fighting Blindness (DGB), and the F.M. Kirby Foundation (DS) and Research to Prevent Blindness (DS).* | *Dr. Sadda has received personal fees from Allergan, Iconic Therapeutics, Novartis, ThromboGenics, Genentech, Heidelberg Engineering, Topcon, CenterVue, and NightstaRx. The remaining authors report no relevant financial disclosures.* |
| **Ogino et al. 2014**(148) | - | *K. Ogino, None; A. Tsujikawa, None; K. Yamashiro, None; S. Ooto, None; A. Oishi, None; I. Nakata, None; M. Miyake, None; A. Takahashi, None; A. A. Ellabban, None; N. Yoshimura, None.* |
| **Ooto et al. 2015**(149) | *Supported by the LuEsther T. Mertz Retinal Research Foundation.* | *S. Ooto, M. Suzuki, and T. Sato received grant from Alcon, Japan. R. F. Spaide is a consultant and received royalties from Topcon. S. Vongkulsiri has no conflicting interests to disclose.* |
| **Owsley et al. 2000**(150) | *Supported by National Institutes of Health (Grants R01 AG04212, R01-EY05627, T32-EY07033); The Foundation Fighting Blindness, Inc.; the Alabama Eye Institute; Research to Prevent Blindness, Inc.; F. M. Kirby Foundation; the Macular Disease Foundation; and the Mackall Trust.* | *N.* |
| **Parisi et al. 2007**(152) | - | *The authors have no proprietary interest in the development or marketing of the instruments used.* |
| **Pfau et al. 2018**(153) | *This study was supported by the BONFOR GEROK Program of the Faculty of Medicine, University of Bonn, Grant No O-137.0022 to MP and Grant No O-137.0020 to ML, and by the German Research Foundation (DFG), Grant No 658/4-1 and 658/4-2 to MF, and Grant No LI2846/1-1 to ML.*  *CenterVue SpA, Padova, Italy has provided research material (S-MAIA) for the conduct of this study.* | *CenterVue had no role in the design or conduct of the experiments.* |
| **Phipps et al. 1999**(155) | - | - |
| **Phipps et al. 2004**(156) | *Supported in part by ARC-Linkage Grant LP0211474 (AJV), a University of Melbourne Research Grant Scheme (AJV, RHG), and the Royal Victorian Institute for the Blind.* | *Disclosure: J.A. Phipps, None; T.M. Dang, Medmont Pty Ltd (R); A.J. Vingrys, Medmont Pty Ltd (F, C); R.H. Guymer, Medmont Pty Ltd (F)* |
| **Pondorfer et al. 2019**(157) | *CenterVue SpA, Padua, Italy has provided research material (S-MAIA) for the conduct of this study.* | *Center-Vue had no role in the design or conduct of the experiments. S. G. Pondorfer: Heidelberg Engineering (F), Optos (F), Carl Zeiss MedicTec (F), CenterVue (F); J. H. Terheyden: Heidelberg Engineering (F), Optos (F), Carl Zeiss MedicTec (F), CenterVue (F); M. Heinemann: Heidelberg Engineering (F), Optos (F), Carl Zeiss MedicTec (F), CenterVue (F); M. W. M. Wintergerst: Heidelberg Engineering (F), Optos (F), Carl Zeiss MedicTec (F), CenterVue (F), Heine Optotechnik (C, F), DigiSight Technologies (F), D-Eye (F) F.G. Holz: Heidelberg Engineering (F, C, R), Optos (F), Carl Zeiss MedicTec (F, C), CenterVue (F), Allergan (F, R), Alcon/Novartis (F, R), Genentech/Roche (F, R), Bayer (F, R), Acucela (F, R), Boehringer Ingelheim (F, R); R.P. Finger: Heidelberg Engineering (F), Optos (F), Carl Zeiss MedicTec (F),, CenterVue (F), Bayer (C), Novartis (C), Santen (C), Opthea (C), Novelion (C), Retina Implant (C), Oxford Innovation (C), Novartis (F).* |
| **Pondorfer et al. 2020 [a]**(158) | *This research was supported by the German Scholars Organization/ Else Kröhner Fresenius Stiftung (GSO/EKFS 16) and BONFOR GEROK Program, Faculty of Medicine, University of Bonn (grant no. O-137.0028, MW).*  *We are grateful for the technical support of Carlo Pellizzari from CenterVue SpA (Padua, Italy), which provided research material (S-MAIA) necessary to conduct this study.* | *S.G. Pondorfer, Heidelberg Engineering (F), Optos (F), Carl Zeiss Medictec (F), CenterVue (F); M.W.M.Wintergerst, Heidelberg Engineering (F), Optos (F), Carl Zeiss Medictec (F), CenterVue (F), Heine Optotechnik (C, F), DigiSight Technologies (F), D-Eye (F); S. Gorgi Zadeh, None; T. Schultz, None; M. Heinemann, Heidelberg Engineering (F), Optos (F), Carl Zeiss Medictec (F), CenterVue (F); F.G. Holz, Heidelberg Engineering (F, C, R), Optos (F), Carl Zeiss Medictec (F, C), CenterVue (F), Allergan (F, R), Alcon/Novartis (F, R), Genentech/Roche (F, R), Bayer (F, R), Acucela (F, R), Boehringer Ingelheim (F, R); R.P. Finger, Heidelberg Engineering (F), Optos (F), Carl Zeiss Medictec (F), CenterVue (F), Bayer (C), Novartis (F, C), Santen (C), Opthea (C), Novelion (C), Retina Implant (C), Oxford Innovation (C)*  *CenterVue had no role in the design or conduct of the experiments.* |
| **Pondorfer et al. 2020 [b]**(159) | *R.P.F.: Else Kro¨hner Fresenius Stiftung/ German Scholars Organization (GSO/EKFS 16) The funders had no role in study design, data collection and analysis, decision to publish, or preparation of the manuscript.* | *NO authors have competing interests* |
| **Prea et al. 2021**(105) | *This study was supported by the National Health & Medical Research Council of Australia (NH&MRC) fellowship grant no. GNT 1103013 (R.H.G.) and the HealthTech Innovation Challenge Prize (A.J.V. and G.K., 2017). The Centre for Eye Research Australia (CERA) received operational infrastructure support from the Victorian Government. The Web-based Research Electronic Data Capture (REDCap) application and open- source platform OpenClinica allowed secure electronic data capture.* | *No sponsor or funding organization had any role in the design or conduct of this research. Financial Disclosures: A.J.V. and G.Y.X.K. are founding directors of Glance Optical Pty. Ltd., the manufacturer of the MRF software. R.H.G. reports personal fees from Bayer, Novartis, Roche Genentech and Apellis. S.M.P, E.B., and P.S. have no financial disclosures.* |
| **Querques et al. 2021**(106) | *Topcon Medical Laser System, Inc. (California, USA) supported the study with a grant.* | *Giuseppe Querques is a consultant for: Alimera Sciences (Alpharetta, Georgia, USA), Allergan Inc (Irvine, California, USA), Bayer Shering-Pharma (Berlin, Germany), Heidelberg (Germany), Novartis (Basel, Switzerland), Sandoz (Berlin, Germany), Zeiss (Dublin, USA). Riccardo Sacconi, Francesco Gelormini, Enrico Borrelli, Francesco Prascina, Ilaria Zucchiatti, Lea Querques: none. Francesco Bandello is a consultant for: Alcon (Fort Worth,Texas,USA), Alimera Sciences (Alpharetta, Georgia, USA), Allergan Inc (Irvine, California,USA), Farmila-Thea (Clermont-Ferrand, France), Bayer Shering-Pharma (Berlin, Germany), Bausch And Lomb (Rochester, New York, USA), Genentech (San Francisco, California, USA), Hoffmann-La-Roche (Basel, Switzerland), Novagali Pharma (Évry, France), Novartis (Basel, Switzerland), Sanofi-Aventis (Paris, France), Thrombogenics (Heverlee,Belgium), Zeiss (Dublin, USA).* |
| **Roh et al. 2019**(160) | *This study was financially supported by the Miller Retina Research Fund (Mass. Eye and Ear), the Champalimaud Vision Award (JWM), the unrestricted departmental grant from Research to Prevent Blindness, New York, and the Portuguese Foundation for Science and Technology/Harvard Medical School Portugal Program (HMSP-ICJ/ 006/2013). None of the aforementioned funding organisations had any role in the design or conduct of this research. DHP is financially supported by the Basic Science Research Program of the National Research Foundation of Korea (NRF), funded by the Ministry of Education (NRF 2017R1D1A1B03027966) and the Korea Health Technology R&D Project of the Korea Health Industry Development Institute (KHIDI), funded by the Ministry of Health & Welfare, Republic of Korea (HI16C1501).* | *None declared.* |
| **Sassmannshausen et al. 2018**(161) | *Supported by Gertrud-Kusen Foundation (JSS), University of Bonn: Bonfor Grant No. O-137.0021 (JSS), University of Bonn: Bonfor Grant No. O-137.0022 (JSS), and the German Research Foundation (DFG) Grants 658/4-1 and 658/4-2 (MF).* | *M. Saßmannshausen, Carl Zeiss MediTec (F), Heidelberg Engineering (F), Optos (F); J.S. Steinberg, Carl Zeiss MediTec (F), Heidelberg Engineering (F), Optos (F); R. Fimmers, None; M. Pfau, Carl Zeiss MediTec (F), Heidelberg Engineering (F), Optos (F); S. Thiele, Carl Zeiss MediTec (F), Heidelberg*  *Engineering (F), Optos (F); M. Fleckenstein, Alcon/Novartis (F, C), Bayer (R), Carl Zeiss MediTec (F), Genentech/Roche (F, C, R), Heidelberg Engineering (F, R), Optos (F); F.G. Holz, Acucela (F, C), Alcon (C), Allergan (F, C), Bayer (F), Bayer Healthcare (C), Bioeg (F), Boehringer Ingelheim (C), Carl Zeiss MediTec (F), Genentech (C), Genentech/Roche (F), Heidelberg Engineering (F, C, R), NightstarX (F), Novartis (F, C), Optos (F, C); S. Schmitz- Valckenberg, Alcon/Novartis (F, C), Allergan (F, R), Bayer (F, R), Bioeg/Formycon (F), Carl Zeiss MediTec (F, R), Centervue (F), Genentech/Roche (F, C, R), Heidelberg Engineering (F, R), Optos (F)*  *The sponsors or funding organizations had no role in the design or conduct of this research.* |
| **Sassmannshausen et al. 2020**(162) | *Supported by research grants of the Rudolf and Anna Katharina Eichenauer-Foundation, the German Research Foundation (Deutsche Forschungsgemeinschaft PF 950/1-1) and in part by an Unrestricted Grant from Research to Prevent Blindness, New York, NY, to the Department of Ophthalmology & Visual Sciences, University of Utah.* | *M. Sassmannshausen, Heidelberg Engineering (F); Carl Zeiss Meditec (F); Optos (F); CenterVue (F); M. Pfau, Heidelberg Engineering (F); Carl Zeiss Meditec (C, F); Optos (F); CenterVue (F); S. Thiele, Heidelberg Engineering (F, R); Carl Zeiss Meditec (F); Optos (F); CenterVue (F); Bayer (R); Novartis (R); R. Fimmers, None; J. Steinberg, None; M. Fleckenstein, Heidelberg Engineering (F); Novartis (C, F); Roche/Genentech (C); US20140303013A1 (P); F. Holz, Acucela (C, F, R); Allergan (F, R); Appelis (C, R); Bayer (C, F, R); Boehringer- Ingelheim (C); Bioeq/Formycon (F, C); CenterVue (F); Ellex (R); Roche/Genentech (C, F, R); Geuder (C); Grayburg Vision (C, R); Heidelberg Engineering (C, F, R); Kanghong (C, F); LinBioscience (C, R); NightStarX (F); Novartis (C, F, R); Optos (F); Pixium Vision (C, F, R); Oxurion (C, R); Stealth BioTherapeutics (C, R); Carl Zeiss Meditec (F, R); S. Schmitz-Valckenberg, Acucela (F); Alcon/Novartis (C, F, R); Allergan (C, F, R); Bayer (F, R); Bioeq/Formycon (F, C); Carl Zeiss MedicTec (F, R); CenterVue (F); Galimedix (C); Genentech/Roche (F, R); Heidelberg Engineering (F); Katairo (F); Optos (F)* |
| **Sassmannshausen et al. 2021**(163) | *Supported by the Rudolf and Anna Katharina Eichenauer-Foundation, Germany; the German Research Foundation (Deutsche Forschungsgemeinschaft grant no.: PF 950/1-1 [M.P.]); the BONFOR Gerok Funding, Faculty of Medicine, University of Bonn, Bonn, Germany (grant nos.: 0-137.0026 [S.T.], FL 658/4-1, FL 658/4-2, and O-237.0381 [M.F.]); and Research to Prevent Blindness, Inc., New York, New York (unrestricted grant to the Department of Ophthalmology and Visual Sciences, University of Utah).* | *M.S.: Nonfinancial support: Heidelberg Engineering, Carl-Zeiss Meditec, Optos, Centervue M.P.: Financial support - Carl Zeiss Meditec; Nonfinancial support: Heidelberg Engineering, Carl Zeiss Meditec, Optos, Centervue S.T.: Financial support - Heidelberg Engineering, Novartis, Bayer; Nonfinancial support - Carl Zeiss Meditec, Optos, Centervue M.F.: Financial support - Bayer, Alcon/Bayer, Genentech/Roche; Nonfinancial support: Heidelberg Engineering, Carl-Zeiss Meditec, Optos, Censtervue; Patent pending: US20140303013A1 F.G.H.: Consultant - Heidelberg Engineering, Bayer, Zeiss, Acuela, Genentech/ Roche, Allergan, Boehringer Ingelheim, Alcon/Novartis, LIN Bioscience, Pixium; Financial support - Heidelberg Engineering, Centervue, Nightstar, Optos, Bayer, Carl Zeiss Meditec, Allergan, Roche/Genentech, Pixium; Lecturer - Roche/Genentech, Zeiss, Bayer, Alcon/Novartis S.-V.: Financial support - Alcon/Novartis, Allergan, Bayer, Bioeq/Formycon, Carl Zeiss Meditec, Gallimedix, Roche, Oxurion, Acucela, Centervue, Heidelberg Engineering, Katairo, Roche; Nonfinancial support: Carl-Zeiss Meditec, Centervue, Heidelberg Engineering, Optos*  *The sponsors or funding organizations had no role in the design or conduct of this research.* |
| **Steinberg et al. 2016**(164) | *GERTRUD KUSEN FOUNDATION, HAMBURG, GERMANY AND ALEXANDER VON HUMBOLDT FOUNDATION, Bonn, Germany.* | *The funding organizations had no role in the design or conduct of this research. Financial disclosures: The Department of Ophthalmology, University of Bonn has received nonfinancial support for supply of technical equipment by several imaging device manufactures, including Heidelberg Engineering GmbH, Optos Ltd, and Carl Zeiss MediTec AG. Retinal imaging in the study was performed by imaging devices manufactured by Heidelberg Engineering. Furthermore, the Department of Ophthalmology, University of Bonn has received research funding from several pharmaceutical companies, including grants from Alcon, Allergan, Bayer, Formycon, Genentech, Novartis, and Roche. Monika Fleckenstein reports personal fees from Alimera, Bayer, Heidelberg Engineering, and Novartis, and has a patent (US20140303013 A1) pending. Akio Oishi has received research grants from Alcon, Japan; the Japan Society for the Promotion of Science, the Ministry of Health Labour and Welfare, Japan; the Takeda Science Foundation, Japan; the Mishima Saiichi kinen Gankakenkyu Kokusaikouryu Kikin, Japan; and the Japan Retinitis Pigmentosa Society; and has received personal fees from Santen and Novartis. Frank G. Holz reports personal fees from Acucela, Alcon, Allergan, Bayer, Boehringer Ingelheim, Genentech, Heidelberg Engineering, Novartis, Optos, and Roche. Steffen Schmitz Valckenberg has received personal fees and honoraria from Alcon/Novartis, Alimera, Heidelberg Engineering, and Optos for consulting and lectures. The following authors have no financial disclosures: Julia S. Steinberg, Marlene Saßmannshausen, and Rolf Fimmers.* |
| **Steinmetz et al. 1993**(165) | *This workwas supported bygrants from The Wolfson Foundation, the Wellcome Trust, and the Medical Research Council of the UK.* | - |
| **Tan et al. 2018**(166) | *Supported by grants from Ryan Initiative for Macular Research, Macular Disease Foundation Australia, Australia Awards Scholarship (RT; Canberra, Australian Capital Territory, Australia), National Health and Medical Research Council (NHMRC) Fellowship (#1103013, RHG; Canberra, Australian Capital Territory, Australia). The Centre for Eye Research Australia (CERA; East Melbourne, Victoria, Australia) receives Operational Infrastructure Support from the Victorian Government.* | *Disclosure: R. Tan, None; R.H. Guymer, None; C.D. Luu, None* |
| **Taylor et al. 2019**(107) | *This study was funded as part of an unrestricted investigator-initiated*  *research grant from Roche Products Ltd, UK, awarded to DPC.* | *Disclaimer The sponsor or funding organization had no role in the design or conduct of this research. Competing interests None declared.* |
| **Tepelus et al. 2017**(167) | *He [Dr. Sadda] receives research support from and serves as a consultant for Allergan, Carl Zeiss Meditec, Genentech, and Optos and has served as a consultant for Alcon, Novartis, and Roche. The remaining authors report no relevant financial disclosures.* | *Dr. Sadda is a co-inventor of Doheny intellectual property related to optical coherence tomography that has been licensed by Topcon Medical Systems and is a member of the scientific advisory board for Heidelberg Engineering.* |
| **Thompson et al. 2018**(168) | *Supported by grants from the National Eye Institute (NEI) Clinical Scientist Development award National Institutes of Health/NEI 5K12 EY016333-08 (EL; Bethesda, MD, USA). The parent clinical study was funded by Hoffmann- La Roche Ltd. (Basel, Switzerland) through a research agreement with Duke University (Durham, NC, USA).* | *A.C. Thompson, None; U.F.O. Luhmann, Roche (E); S.S. Stinnett, None; L. Vajzovic, Alcon (F), Janssen (R), Knights Templar Eye Foundation (R), PDCs ENABLE (R), Roche (F), Second Sight (F), F. Hoffmann-La Roche Ltd (E); A. Horne, None; C.A. Toth, Alcon (R), P; S.W. Cousins, Alimera (C), Bausch&Lomb (C, F), PanOptica (C), Stealth (C, F), Therakine (C); E.M. Lad, Hoffman (F), Roche (F), Apellis (F), Janssen Research (C), Genentech (C)* |
| **Uddin et al. 2020**(169) | *Supported by the National Eye Institute Intramural Research Program, National Institutes of Health (NIH), Bethesda, Maryland; and the NIH Medical Research Scholars Program, a public-private partnership supported jointly by the NIH and generous contributions to the Foundation for the NIH from the Doris Duke Charitable Foundation, the American Association for Dental Research, the Colgate-* *Palmolive Company, Genentech, Elsevier, and other private donors.* | *Disclosure: D. Uddin, None; B.G. Jeffrey, None; O. Flynn, None; W. Wong, None; H. Wiley, None; T. Keenan, None; E. Chew, None; C. Cukras, None* |
| **Vujosevic et al. 2011**(170) | - | - |
| **Vujosevic et al. 2017**(171) | *No funding was received for this research.* | *All authors certify that they have no affiliations with or involvement in any organization or entity with any financial interest (such as honoraria; educational grants; participation in speakers’ bureaus; membership, employment, consultancies, stock ownership, or other equity interest; and expert testimony or patent-licensing arrangements), or non-financial interest (such as personal or professional relationships, affiliations, knowledge or beliefs) in the subject matter or materials discussed in this manuscript.* |
| **Weingessel et al. 2009**(172) | *No funding or support was provided* | *None of the authors have any commercial interest in any material or method mentioned* |
| **Welker et al. 2018**(173) | *Supported by the German Scholars Organization/Else Kr¨ohner Fresenius Stiftung (GSO/EKFS 16).* *CenterVue SpA, Padua, Italy, has provided research material (S-MAIA) for this study.* | *S.G. Welker, Heidelberg Engineering (F), Optos (F), Carl Zeiss MedicTec (F), CenterVue (F); M. Pfau, Heidelberg Engineering (F), Optos (F), Carl Zeiss MedicTec (F), CenterVue (F); M. Heinemann, Heidelberg Engineering (F), Optos (F), Carl Zeiss MedicTec (F), CenterVue (F); S. Schmitz Valckenberg, Heidelberg Engineering (F, R), Optos (F), Carl Zeiss MedicTec (F), CenterVue (F), Allergan (F, R), Alcon/Novartis (F, R), Bioeq/ Fermycon (F), Genentech/Roche (F, R), Bayer (F, R); F.G. Holz, Heidelberg Engineering (F, C, R), Optos (F), Carl Zeiss MedicTec (F, C), CenterVue (F), Allergan (F, R), Alcon/Novartis (F, R), Genentech/Roche (F, R), Bayer (F, R), Acucela (F, R), Boehringer Ingelheim (F, R); R.P. Finger, Heidelberg Engineering (F), Optos (F), Carl Zeiss MedicTec (F), CenterVue (F), Bayer (C), Novartis (C), Santen (C), Opthea (C), Novelion (C), Retina Implant (C), Oxford Innovation (C), Novartis (F)*  *CenterVue had no role in the design or conduct of the experiments.* |
| **Wightman et al 2019**(174) | *Supported by National Health and Medical Research Council (NHMRC) Fellowship (#1103013, RHG) and NHMRC Project Grant (1084081). The Centre for Eye Research Australia (CERA) receives operational infrastructure support from the Victorian State Government.* | *Disclosure: A.J. Wightman, None; C.J. Abbott, None; M.B. McGuinness, None; E. Caruso, None; R.H. Guymer, None; C.D. Luu, None* |
| **Wu et al. 2013**(175) | *Supported by National Health and Medical Research Council (NH&MRC) Project Grant 1027624, NH&MRC Practitioner Fellowship 529905 (RHG), a Macular Disease Foundation Research grant, the Bupa Health Foundation (Australia), a William Angliss (Victoria) Charitable Fund Research grant, and Murray Redvers and Rodney Alastair Brownless Perpetual Charitable Trust funding*  *administered by the Trust Company. The Centre for Eye Research*  *Australia receives operational infrastructure support from the*  *Victorian Government and is supported by NH&MRC Centre for*  *Clinical Research Excellence Award 529923.* | *Z. Wu, None; L.N. Ayton, None; R.H. Guymer, None; C.D. Luu, None* |
| **Wu et al. 2014 [a]**(176) | *Supported by the National Health and Medical Research Council (NH&MRC) Project Grant (1027624); NH&MRC Practitioner Fellowship (RHG, #529905); Macular Disease Foundation Australia (MDFA) Research Grant; Bupa Health Foundation (Australia); The Menzies Foundation; a University of Melbourne Early Career Research grant (LNA, #1350114); and the Macular Vision Loss Support Society of Australia, Inc. CERA receives Operational Infrastructure Support from the Victorian Government and is supported by a NH&MRC Centre for Clinical Research Excellence award (#529923).* | *Disclosure: Z. Wu, None; L.N. Ayton, None; C.D. Luu, None; R.H. Guymer, None* |
| **Wu et al. 2014 [b]**(177) | *Supported by the National Health and Medical Research Council (Canberra, ACT, Australia) (Project Grant no.: 1027624; practitioner fellowship no.: 529905 [RHG]); the Macular Disease Foundation (Sydney, NSW, Australia); the Bupa Health Foundation (Melbourne, VIC, Australia); William Angliss Charitable Fund (Melbourne, VIC, Australia); and the MR & RA Brownless Perpetual Charitable Trust (Melbourne, VIC, Australia). The Centre for Eye Research Australia receives Operational Infrastructure Support from the Victorian Government and is supported by the National Health and Medical Research Council (Centre for Clinical Research Excellence Award no.: 529923).* | *The author(s) have no proprietary or commercial interest in any materials discussed in this article.* |
| **Wu et al. 2014 [c]**(178) | *Supported by the National Health and Medical Research Council (NH&MRC) Project Grant (1027624), NH&MRC practitioner fellowship (RHG, #529905), Macular Disease Foundation Australia Research Grant, Bupa Health Foundation (Australia), William Angliss (Victoria) Charitable Fund Research Grant, BrightFocus Foundation, The Perpetual Trust Foundation and MR & RA Brownless Perpetual Charitable Trust funding administered by the Trust Company. The Centre for Eye Research Australia receives Operational Infrastructure Support from the Victorian Government. This study was supported by the NH&MRC Centre for Clinical Research Excellence #529923 e Translational Clinical Research in Major Eye Diseases.* | *The author(s) have no proprietary or commercial interest in any materials discussed in this article.* |
| **Wu et al. 2014 [d]**(179) | *Supported by National Health and Medical Research Council (NHMRC) project grant 1027624 and NHMRC practitioner fellowship grant 529905 (RHG) and by a Macular Disease Foundation research grant, the Bupa Health Foundation (Australia), and the Macular Vision Loss Support Society of Australia Inc. Centre for Eye Research Australia receives operational infrastructure support from the Victorian government and is supported by NHMRC Centre for Clinical Research Excellence award 529923.* | *Z. Wu, None; L.N. Ayton, None; R.H. Guymer, None; C.D. Luu, None*  *The authors alone are responsible for the content and writing of the paper.* |
| **Wu et al. 2015 [a]**(180) | *Supported by the Australian Research Council (AMM, FT0990930 and AT, FT0991326), the National Health and Medical Research Council (NH&MRC) Project Grant (1027624), Macular Disease Foundation Australia (MDFA) Research Grant, Bupa Health Foundation (Australia) and The Macular Vision Loss Support Society of Australia, Inc. CERA receives Operational Infrastructure Support from the Victorian Government and is supported by a NHMRC Centre for Clinical Research Excellence Award (#529923).* | *Disclosure: Z. Wu, None; R.H. Guymer, None; C.J. Jung, None; J.K. Goh, None; L.N. Ayton, None; C.D. Luu, None; D.J. Lawson, None; A. Turpin, None; A.M. McKendrick, None* |
| **Wu et al. 2015 [b]**(181) | *This research was supported by project grant 1027624 fromthe National Health and Medical Research Council (NHMRC), a research grant from the Macular Disease Foundation Australia (MDFA), Bupa Health Foundation (Australia), BrightFocus Foundation, and The Menzies Foundation. The Centre for Eye Research Australia receives operational infrastructure support from the Victorian government and is supported by excellence award 529923 from the National Health and Medical Research Council Center for Clinical Research.* | *All authors have completed and submitted the ICMJE Form for Disclosure of Potential Conflicts of Interest and none were reported.*  *The funders had no role in the design and conduct of the study; collection, management, analysis, and interpretation of the data; preparation, review, or approval of the manuscript; and decision to submit the manuscript for publication.* |
| **Wu et al. 2015 [c]**(182) | *Supported by the National Health and Medical Research Council (NHMRC) Project Grant (#1027624; Canberra, ACT, Australia), Macular Disease Foundation Australia (MDFA) Research Grant (Sydney, NSW, Australia), Bupa Health Foundation (Sydney, NSW, Australia), BrightFocus Foundation (Clarksburg, MD, USA), a University of Melbourne Early Career Research Grant (LNA, #1350114; Parkville, VIC, Australia) and the Menzies Foundation (East Melbourne, VIC, Australia). CERA receives Operational Infrastructure Support from the Victorian Government and is supported by a NHMRC Centre for Clinical Research Excellence Award (#529923).* | *Z. Wu, None; L.N. Ayton, None; G. Makeyeva, None; R.H. Guymer, None; C.D. Luu, None* |
| **Wu et al. 2016 [a]**(183) | *Supported by the National Health and Medical Research Council (NHMRC) Project Grant (1027624), Macular Disease Foundation Australia (MDFA) Research Grant, Bupa Health Foundation (Australia), and BrightFocus Foundation. Centre for Eye Research Australia receives operational infrastructure support from the Victorian government and is supported by an NHMRC Centre for Clinical Research Excellence Award (529923).* | *Z. Wu, None; D. Cunefare, None; E. Chiu, None; C.D. Luu, None; L.N. Ayton, None; C.A. Toth, P; S. Farsiu, P; R.H. Guymer, None* |
| **Wu et al. 2016 [b]**(184) | *This work was in part supported by the German Research Council (DFG FI 1540/5-1, grant to RPF) and by National Health and Medical Research Council (NHMRC) project grants (#590205 and #1008979) and Centre for Clinical Research Excellence grant #529923, a Macular Disease Foundation Australia Research Grant (RHG), the BrightFocus Foundation and the Charles Viertel Charitable Foundation, the Lloyd and Kathleen Ansell Ophthalmology Foundation, the Mankiewicz-Zelkin Fellowship of the University of Melbourne and The Menzies Foundation.* | *None declared.*  *The funders had no role in the design and conduct of the data, preparation, review or approval of the manuscript and decision to submit the manuscript for publication.* |
| **Wu et al. 2019**(185) | *Supported by National Health & Medical Research Council of Australia (project grant APP1027624 [R.H.G. and C.D.L.], and fellowship grant GNT1103013 (R.H.G.), APP1104985 [Z.W.], APP1054712 [F.K.C.], APP1142962 [F.K.C.] and GNT1128343 [S.S.W.]), and BUPA Health Foundation (Australia) (R.H.G. and C.D.L.). The Centre for Eye Research Australia receives operational infrastructure support from the Victorian Government. Ellex R&D Pty Ltd. (Adelaide, Australia) provided partial funding of the central coordinating center and the in-kind provision of Ellex 2RT laser systems, ongoing support of those systems, and the Macular Integrity Assessment microperimeters for the duration of the study. The web-based Research Electronic Data Capture (REDCap) application and open-source platform OpenClinica allowed secure electronic data capture. The study is sponsored by the Centre for Eye Research Australia, an independent medical research institute and a not-for-profit company.* | *R.H.G.: Personal fees – Bayer, Novartis, Roche Genentech, and Apellis outside the submitted work; Research grant – Bayer outside the submitted work. F.K.C.: Personal fees – Bayer, Novartis, Allergan, Heidelberg Engineering, Alcon, and Pfizer outside the submitted work; Research grant – Novartis and Bayer outside the submitted work. U.C.: Personal fees Bayer, Novartis, and Roche outside the submitted work; Research grant – Bayer, Novartis, and Roche outside the submitted work. J.J.A.: Personal fees from Allergan, Bayer, and Novartis. C.A.H.: Personal fees – Novartis, Bayer, and Allergan and a director of Avalanche Australia outside the submitted work. S.S.W.: Personal fees – Allergan and Bayer outside the submitted work. W.J.H.: Personal fees – Alcon, Bayer, and Novartis outside the submitted work. R.P.F.: Personal fees – Novartis, Bayer, Santen, Opthea, Novelion, Retina Implant, Oxford Innovation; Research grant – Novartis outside the submitted work.* |
| **Wu et al. 2020**(186) | *This study was supported by National Health & Medical Research Council of Australia (project grant no.: APP1027624 (RHG and CDL), and fellowship grant no.: GNT1103013 (RHG), APP1104985 (ZW), APP1054712 (FKC) and APP1142962 (FKC)) and BUPA Health Foundation (Australia) (RHG and CDL). CERA receives operational infrastructure support from the Victorian Government. The web-based Research Electronic Data Capture (REDCap) application and open-source platform OpenClinica allowed secure electronic data capture. The study is sponsored by the Centre for Eye Research Australia (CERA), an independent medical research institute and a not-for-profit company.* | *RHG reports personal fees from Bayer, Novartis, Roche Genentech and Apellis outside the submitted work and research grant from Bayer outside the submitted work. FKC reports personal fees from Bayer, Novartis, Allergan, Heidelberg Engineering, Alcon and Pfizer outside the submitted work and aresearch grant from Novartis and Bayer outside the submitted work. UC reports personal fees from Bayer, Novartis, Roche outside the submitted work and aresearch grant from Bayer, Novartis, Roche outside the submitted work. JJA reports personal fees from Allergan, Bayer and Novartis. WJH reports personal fees from Alcon, Bayer and Novartis outside the submitted work. JR reports personal fees from Ellex Medical Lasers Ltd outside the submitted work. ZW, CDL, LABH and EC report nothing to declare.* |
| **Wu et al. 2021**(187) | *Supported by the National Health and Medical Research Council of Australia (project grant no.: APP1027624 [R.H.G. and C.D.L.], and fellowship grant no.: GNT1103013 [R.H.G.], APP1104985 [Z.W.], APP1054712 [F.K.C.], and APP1142962 [F.K.C.]) and BUPA Health Foundation (Australia) (R.H.G. and C.D.L.). The Centre for Eye Research Australia receives operational infrastructure support from the Victorian Government. The web based Research Electronic Data Capture (REDCap) application and open-source platform OpenClinica allowed secure electronic data capture. The study is sponsored by the Centre for Eye Research Australia (CERA), an independent medical research institute and a not-for-profit company.* | *R. H. Guymer reports personal fees from Bayer, Novartis, Roche, Genentech, and Apellis outside the submitted work and research grant from Bayer outside the submitted work. F. K. Chen reports personal fees from Bayer, Novartis, Allergan, Heidelberg Engineering, Alcon, and Pfizer outside the submitted work and a research grant from Novartis and Bayer outside the submitted work. U. Chakravarthy reports personal fees from Bayer, Novartis, and Roche outside the submitted work and a research grant from Bayer, Novartis, and Roche outside the submitted work. J. J. Arnold reports personal fees from Allergan, Bayer, and Novartis. W. J. Heriot reports personal fees from Alcon, Bayer, and Novartis outside the submitted work. J. Runciman reports personal fees from Ellex Medical Lasers Ltd outside the submitted work. The remaining authors have no conflicting interests to disclose.* |
| **Zhang et al. 2022**(188) | *Supported by NIH R01EY024378, R21EY027948, R01EY029595, R01AG04212, P30EY003039, W F Keck Foundation, the Carl Marshall Reeves & Mildred Almen Reeves foundation, Research to Prevent Blindness/Dr. H. James and Carole Free Catalyst Award for Innovative Research Approaches for AMD, Research to Prevent Blindness institutional funds, EyeSight Foundation of Alabama, and the Buck Trust of Alabama. The sponsors or funding organizations had no role in the design or conduct of this research.* | *Y. Zhang, None; T.A. Swain, None; M.E. Clark, None; K.R. Slone, None; W. Warriner, None; C. Owsley, None; S.R. Sadda, Allergan (F,C), Carl Zeiss Meditec (F), Genentech (F, C), Optos (F,C), Topcon (F), Amgen (C), Apellis (C), Iveric (C), Centervue (C), Roche (C), Heidelberg (C), 4DMT (C), Bayer (C), Regeneron (C), Novartis (C), Oxurion (C); D. Sarraf, Amgen (F, C), Bayer (C), Genentech (C, F), Iveric Bio (C), Novartis (C), Optovue (F, C), Heidelberg (F), Regeneron (F)* *and Topcon V (F); C.A. Curcio, Genentech (F), Regeneron (F), MacRegen (I)* |

**Supplementary Table 6. Risk of bias assessment.**

Risk of bias summary figure, adapted from Whiting *et al.*(190) and Wells *et al*.(191) All studies were assessed for risk of bias regarding patient selection and comparability of study groups according to the primary outcome without sub-grouping. Sponsorship bias was excluded due to its controversial status as a risk of bias domain.^168,169^ These established guides were used to frame signalling questions (yes, no, or unclear) and risk of bias (high, low, or unclear) for observational studies according to the primary outcome without sub-grouping. A high risk of bias was defined as a ‘no’ to any signalling question in the respective domain. A low risk of bias was defined as a ‘yes’ to all signalling questions in the respective domain. Unclear risk of bias was defined when there was insufficient study data to determine whether there was high or low risk of bias in the respective domain. Studies without a comparative normal group such as cross-sectional or cohort studies were considered at high risk of bias for the latter domain.

Abbreviations: high risk of bias, low risk of bias.

| **Study** | **Patient selection** | **Comparability of study groups** |
| --- | --- | --- |
| **Adams et al. 2018**(110) |  |  |
| **Broadhead et al. 2017**(112) |  |  |
| **Broadhead et al. 2020**(114) |  |  |
| **Chandramohan et al. 2016**(115) |  |  |
| **Chen et al. 2004**(116) |  |  |
| **Clemens et al. 2015**(117) |  |  |
| **Cocce et al. 2018**(118) |  |  |
| **Corvi et al. 2019**(119) |  |  |
| **Dinc et al. 2008**(120) |  |  |
| **Dow et al. 2016**(103) |  |  |
| **Echols et al. 2020**(121) |  |  |
| **Fragiotta et al. 2017**(123) |  |  |
| **Fragiotta et al. 2022**(124) |  |  |
| **Gin et al. 2011**(125) |  |  |
| **Goh et al. 2022**(189) |  |  |
| **Grewal et al. 2021**(129) |  |  |
| **Guymer et al. 2014**(104) |  |  |
| **Guymer et al. 2021**(130) |  |  |
| **Haimovici et al. 2002**(131) |  |  |
| **Higgins et al. 2020**(132) |  |  |
| **Hsu et al 2019**(133) |  |  |
| **Huang et al. 2015**(134) |  |  |
| **Iwama et al. 2010**(135) |  |  |
| **Kar et al. 2020**(136) |  |  |
| **Leal et al. 2022**(137) |  |  |
| **Luu et al. 2012**(138) |  |  |
| **Luu et al. 2013**(139) |  |  |
| **Maynard et al. 2016**(140) |  |  |
| **McGuinness et al. 2019**(141) |  |  |
| **McGuinness et al. 2020**(142) |  |  |
| **Messenio et al. 2022**(143) |  |  |
| **Midena et al. 2007**(144) |  |  |
| **Montesano et al. 2020**(145) |  |  |
| **Nassisi et al. 2021**(146) |  |  |
| **Nittala et al. 2019**(147) |  |  |
| **Ogino et al. 2014**(148) |  |  |
| **Ooto et al. 2015**(149) |  |  |
| **Owsley et al. 2000**(150) |  |  |
| **Parisi et al. 2007**(152) |  |  |
| **Pfau et al. 2018**(153) |  |  |
| **Phipps et al. 1999**(155) |  |  |
| **Phipps et al. 2004**(156) |  |  |
| **Pondorfer et al. 2019**(157) |  |  |
| **Pondorfer et al. 2020 [a]**(158) |  |  |
| **Pondorfer et al. 2020 [b]**(159) |  |  |
| **Prea et al. 2021**(105) |  |  |
| **Querques et al. 2021**(106) |  |  |
| **Roh et al. 2019**(160) |  |  |
| **Sassmannshausen et al. 2018**(161) |  |  |
| **Sassmannshausen et al. 2020**(162) |  |  |
| **Sassmannshausen et al. 2021**(163) |  |  |
| **Steinberg et al. 2016**(164) |  |  |
| **Steinmetz et al. 1993**(165) |  |  |
| **Tan et al. 2018**(166) |  |  |
| **Taylor et al. 2019**(107) |  |  |
| **Tepelus et al. 2017**(167) |  |  |
| **Thompson et al. 2018**(168) |  |  |
| **Uddin et al. 2020**(169) |  |  |
| **Vujosevic et al. 2011**(170) |  |  |
| **Vujosevic et al. 2017**(171) |  |  |
| **Weingessel et al. 2009**(172) |  |  |
| **Welker et al. 2018**(173) |  |  |
| **Wightman et al 2019**(174) |  |  |
| **Wu et al. 2013**(175) |  |  |
| **Wu et al. 2014 [a]**(176) |  |  |
| **Wu et al. 2014 [b]**(177) |  |  |
| **Wu et al. 2014 [c]**(178) |  |  |
| **Wu et al. 2014 [d]**(179) |  |  |
| **Wu et al. 2015 [a]**(180) |  |  |
| **Wu et al. 2015 [b]**(181) |  |  |
| **Wu et al. 2015 [c]**(182) |  |  |
| **Wu et al. 2016 [a]**(183) |  |  |
| **Wu et al. 2016 [b]**(184) |  |  |
| **Wu et al. 2019**(185) |  |  |
| **Wu et al. 2020**(186) |  |  |
| **Wu et al. 2021**(187) |  |  |
| **Zhang et al. 2022**(188) |  |  |

**Supplementary Table 7. Modulating factors.**

Exploration of between-study sub-groups and moderators, via sub-group meta-analyses and meta-regression respectively. *P* values were considered after Bonferroni correction, e.g., *P* < $\frac{0.05}{6}$ would be considered significant for six sub-group comparisons. Df, degrees of freedom.

| **Early/intermediate AMD versus normal** | | | | |
| --- | --- | --- | --- | --- |
| **Sub-groups** |  | **Q(df)** | **P value** | **I^2^** |
|  | AMD classification | Q(3) 1.05 | 0.79 | 0% |
|  | Device | Q(4) 15.16 | **0.004** | 73.6% |
|  | Test pattern | Q(2) 3.3 | 0.19 | 39.5% |
|  | Mesopic or scotopic | Q(1) 6.72 | 0.01 | 85.1% |
|  | Stimuli size and chromaticity | Q(2) 7.33 | 0.03 | 72.7% |
|  | Pupils dilated or undilated | Q(1) 0.03 | 0.86 | 0% |
| **Moderators** |  | **Regression co−efficient** | **P value** | - |
|  | Testing radius | 0.01 [−0.02, 0.05] | 0.5 | - |
|  | Background luminance | −0.09 [−0.25, 0.04] | 0.26 | - |
|  | Adaptation time | 0.01 [0, 0.01] | 0.24 | - |
| **Intermediate AMD versus normal** | | | | |
| **Sub-groups** |  | **Q(df)** | **P value** | **I^2^** |
|  | AMD classification | Q(2) 0.56 | 0.76 | 0% |
|  | Device | Q(3) 4.71 | 0.19 | 36.3% |
|  | Test pattern | Q(1) 3.06 | 0.08 | 67.3% |
|  | Mesopic or scotopic | Q(1) 2.04 | 0.15 | 50.9% |
|  | Stimuli size and chromaticity | Q(1) 1.1 | 0.29 | 8.9% |
|  | Pupils dilated or undilated | Q(1) 0.26 | 0.61 | 0% |
| **Moderators** |  | **Regression co−efficient** | **P value** | - |
|  | Testing radius | −0.01 [−0.07, 0.06] | 0.84 | - |
|  | Background luminance | −0.21 [−0.61, 0.19] | 0.28 | - |
|  | Adaptation time | 0.01 [−0.01, 0.02] | 0.47 | - |
| **Early AMD versus normal** | | | | |
| **Sub-groups** |  | **Q(df)** | **P value** | **I^2^** |
|  | AMD classification | Q(1) 1.47 | 0.23 | 31.8% |
| **Intermediate AMD versus early AMD** | | | | |
| **Sub-groups** |  | **Q(df)** | **P value** | **I^2^** |
|  | AMD classification | Q(1) 4.22 | 0.04 | 76.3% |
|  | Pupils dilated or undilated | Q(1) 0.02 | 0.88 | 0% |
| **Moderators** |  | **Regression co−efficient** | **P value** | - |
|  | Testing radius | 0.02 [−0.04, 0.08] | 0.46 | - |
|  | Background luminance | 0.12 [−0.12, 0.36] | 0.27 | - |
|  | Adaptation time | 0 [−0.03, 0.02] | 0.71 | - |

Supplementary Table 8. GRADE assessment of studies in meta-analyses.

GRADE assessment was according to criteria for observational studies.(192,193) The overall quality of evidence begins as ‘low’, by default. Factors can reduce or increase the quality of evidence between ‘very low’, ‘low’, ‘moderate’ and ‘high’ certainty of evidence.

|  | **Factors that can reduce the quality of the evidence** | | | | | **Factors that can increase the quality of the evidence** | | |  |
| --- | --- | --- | --- | --- | --- | --- | --- | --- | --- |
|  | **Study limitations** | **Inconsistency of results** | **Indirectness of evidence** | **Imprecision** | **Publication bias** | **Large magnitude of effect** | **Dose-response effect** | **Plausible confounding reduced effect** | **Overall quality of evidence** |
| **Consequence** | 0 | −1 | 0 | 0 | 0 | +1 | +1 | 0 | Moderate |
| **Reasoning** | No significant study limitations beyond observational study designs. | Heterogeneity only partly explained by sub-group meta-analyses. | Population, intervention (diagnostic test), and outcome measure are all clinically relevant. | Very large total sample size and summary effect size confidence intervals do not cross zero. | Potential publication bias, although adjusted effect size did not significantly alter outcomes nor conclusions, particularly as effect sizes varied up to very large (−1.76 Hedge’s g). | Mostly large magnitudes of effect sizes. | Global MS worsened with intermediate versus early AMD. | Confounding unlikely to have reduced magnitude of effect size. | Baseline ‘low’  −1  +1  +1 |

**Supplementary Table 9. Qualitative data for secondary outcome.**

Data used for qualitative assessment of the secondary outcome, i.e., any real-world patient outcome such as quality of life and/or activities of daily living indices, related to the primary outcome. Correlation strength were interpreted according to Schober et al, i.e., |r| < 0.1 = negligible, < 0.4 = weak, < 0.7 = moderate, < 0.9 = strong, and ≥ 0.9 = very strong.(12)

| **Study** | **AMD classification** | **Testing Conditions** | **Results summary** |
| --- | --- | --- | --- |
| **Broadhead et al. 2020**(114) | AREDS 4 step:(113) early + intermediate | MAIA, 37 stimuli within 5° radius  Full threshold, GIII, 1.27cd/m^2^ | **NEI VFQ**   - Global MS significantly correlated to various sub-scales for the categories: general vision, near activities, distance activities, social functioning, and colour vision. Magnitude of these correlations were weak.   - No significant correlations with the categories: composite total score, peripheral vision, driving, role difficulties, dependency, mental health, ocular pain, and general health.   - No other modalities, i.e., flicker perimetry, multifocal electroretinography, or best corrected visual acuity, were significantly correlated with any sub-scales in any category, except multifocal electroretinography latency with general vision. |
| **Higgins et al. 2020**(132) | Beckman Initiative:(111) early + intermediate | MAIA, 37 stimuli within 5° radius  Full threshold, GIII, 1.27cd/m^2^ | **Miscellaneous – computerised visual search and road sign identification tasks**   - Global MS significantly correlated to the computerised: visual search task, and single road signs identification task. Magnitude of these correlations were moderate.   - No significant correlation to the computerised: double road signs identification task. |
| **McGuinness et al. 2019**(141)  **[repeated data in McGuinness et al. 2020]**(142)  **[part of the LEAD study]**(127,128) | Beckman Initiative:(111) intermediate | MAIA, 37 stimuli within 5° radius  Full threshold, GIII, 1.27cd/m^2^ | **IVI**   - Global MS significantly correlated to the composite total score. Magnitude of this correlation was weak. - Other modalities, i.e., best corrected visual acuity and low luminance visual acuity, were also significantly correlated to the composite total score. Magnitude of these correlations were weak.   **NVQ**   - Global MS significantly correlated to the composite total score. Magnitude of this correlation was weak. - Other modalities, i.e., best corrected visual acuity and low luminance visual acuity, were also significantly correlated to the composite total score. Magnitude of these correlations were weak. |
| **Pondorfer et al. 2019**(157) | Beckman Initiative:(111) early + intermediate | S-MAIA, 33 stimuli within 7° radius  Full threshold^◊^, GIII^◊^, 1.27cd/m^2◊^ and 0cd/m^2◊^ | **IVI**   - Global MS not significantly associated with any of the categories: reading, mobility, and emotional well-being. |
| **Taylor et al. 2019**(107) | Beckman Initiative:(111) early + intermediate | MAIA, 37 stimuli within 5° radius  Full threshold, GIII, 1.27cd/m^2^ | **Miscellaneous – perceived anxiety via computerised mobility tasks**   - Global MS not significantly associated with perceived anxiety via computerised mobility tasks. - Other modalities, i.e., best corrected visual acuity, contrast sensitivity, and EQ-5D QoL questionnaire, were also not significantly associated with perceived anxiety via computerised mobility tasks. |
| **Thompson et al. 2018**(168) | AREDS 4 step:(113) early + intermediate | MAIA, 37 stimuli within 5° radius  Full threshold, GIII, 1.27cd/m^2^ | **LLQ**   - Global MS not significantly associated with the composite total score. - Other modalities, i.e., computerised low luminance visual acuity and low luminance deficit, were significantly associated with the composite total score. |
| **Wu et al. 2016 [b]**(184) | Beckman Initiative:(111) intermediate | MAIA, 37 stimuli within 6° radius  Full threshold, GIII, 1.27cd/m^2^ | **NVQ**   - Global MS and central MS not significantly associated with the composite total score. - Other modality, i.e., low luminance deficit, was significantly associated with the composite total score. |

Supplementary Table 10. Comparison of meta-analyses values from this review to test-retest variability per device.

Summary of three relevant studies which report the global MS coefficient of repeatability (CoR) – an absolute measurement index of variability accounting for random and systematic error, equating to a value wherein the absolute difference between repeated measures would lie with 95% probability.(194,195) Effect size (Hedge’s g) was converted to device-specific mean difference (dB), where Hedge’s g = sample mean difference divided by SD_pooled_ with correction for small samples.(4)

| **Study** | **Population** | **Device** | **CoR (intra- or inter-session)** | **Meta-analyses values** |
| --- | --- | --- | --- | --- |
| **Grewal et al. 2021**(129) | Normal eyes | Medmont DACP (scotopic) | 1.62dB (cyan stimuli, inter-session)  3.35dB (cyan stimuli, intra-session)  2.66dB (red stimuli, inter-session)  2.03dB (red stimuli, intra-session) | Insufficient number of studies for meta-analysis |
| **Wong et al. 2017**(196) | Normal eyes | **MP-1 (mesopic)** | 1.1dB (inter-session) | **−1.72 Hedge’s g 🡪 −5.25dB** |
| **Wu et al. 2013**(197) | Normal and early/intermediate  AMD eyes | **MAIA (mesopic)** | 1.1dB (intra-session, normal eyes)  1.08dB (intra-session, early/iAMD eyes)  1.56dB (intra-session, different sub-set of AMD eyes) | **−0.99 Hedge’s g 🡪 −2.9dB** |

# Supplementary Figures


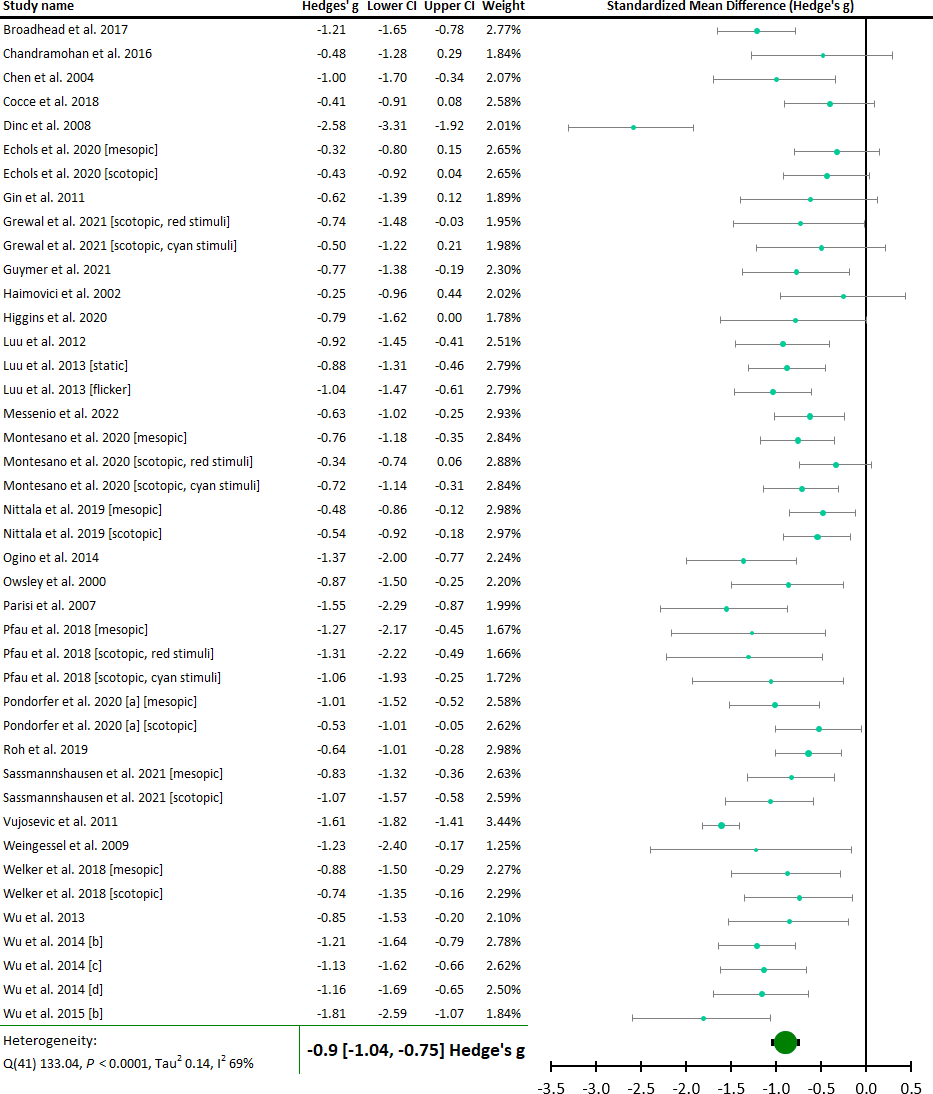


Supplementary Figure 1. Forest plot of global MS for early/intermediate AMD versus normal, sub-grouped by device and ordered by effect size.

Negative values indicate reduced global MS in early/intermediate AMD groups. Summary data presented as standardised Hedge’s g [CI]. Study [label] provided for unique identification. Note the grouping of studies with varying testing protocol was validated through sub-group meta-analyses and *post hoc* sensitivity analysis.


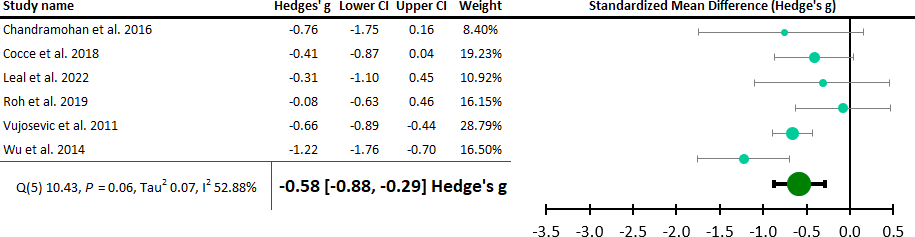


Supplementary Figure 2. Forest plot of global MS for intermediate AMD versus early AMD.

Presentation as in Supplementary Figure 1.


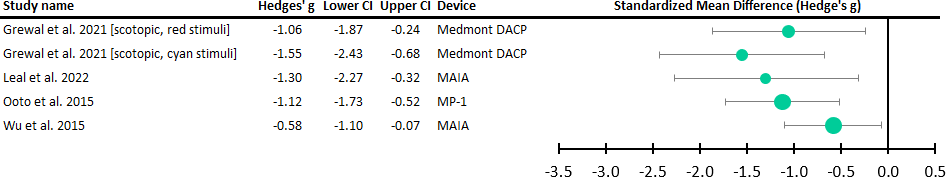


**Supplementary Figure 3. Forest plot of global MS for early/intermediate AMD with RPD versus without RPD, for individual studies.**

Presentation as in Supplementary Figure 1.


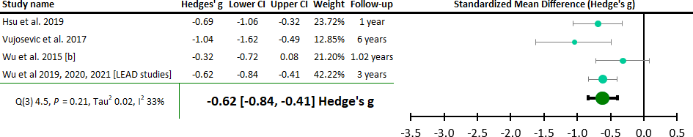


Supplementary Figure 4. Forest plot of global MS for early/intermediate AMD at follow-up versus baseline.

Presentation as in Supplementary Figure 1.


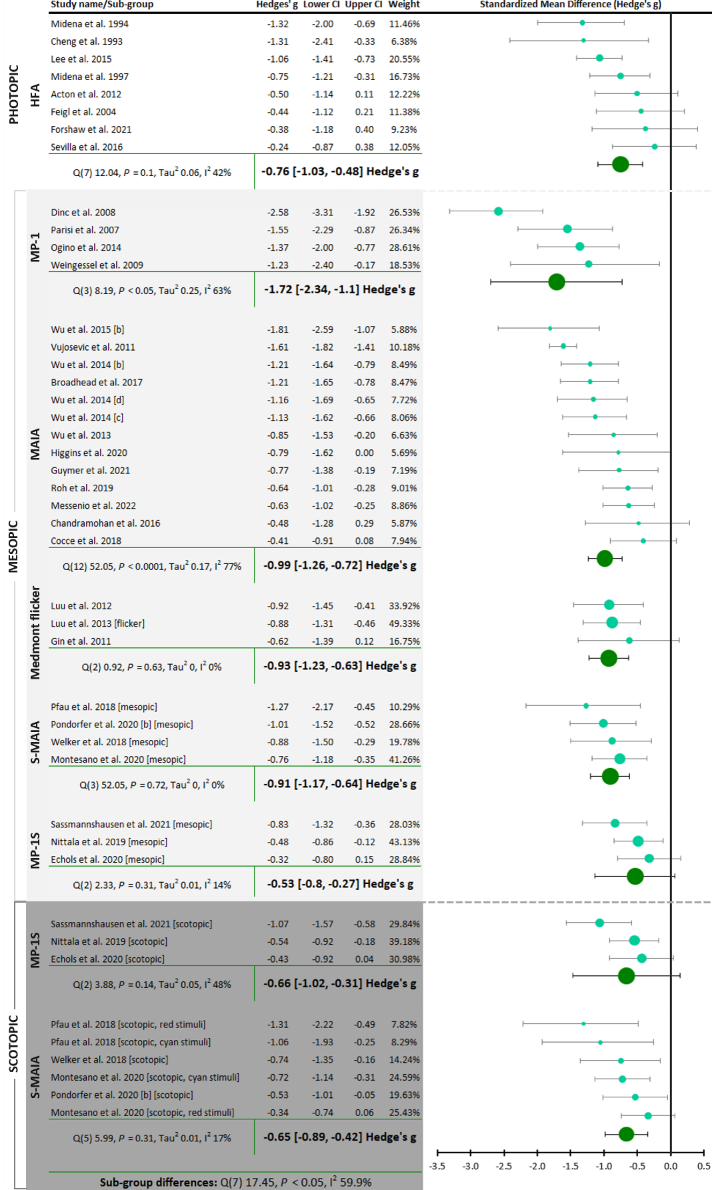


Supplementary Figure 5. Forest plot of global MS for early/intermediate AMD versus normal, sub-grouped and ordered by lighting and device.

Presentation as in Supplementary Figure 1. Photopic device depicted in *white*, mesopic devices in *light grey*, and scotopic devices in *dark grey*. Note devices can have more than one background luminance setting.


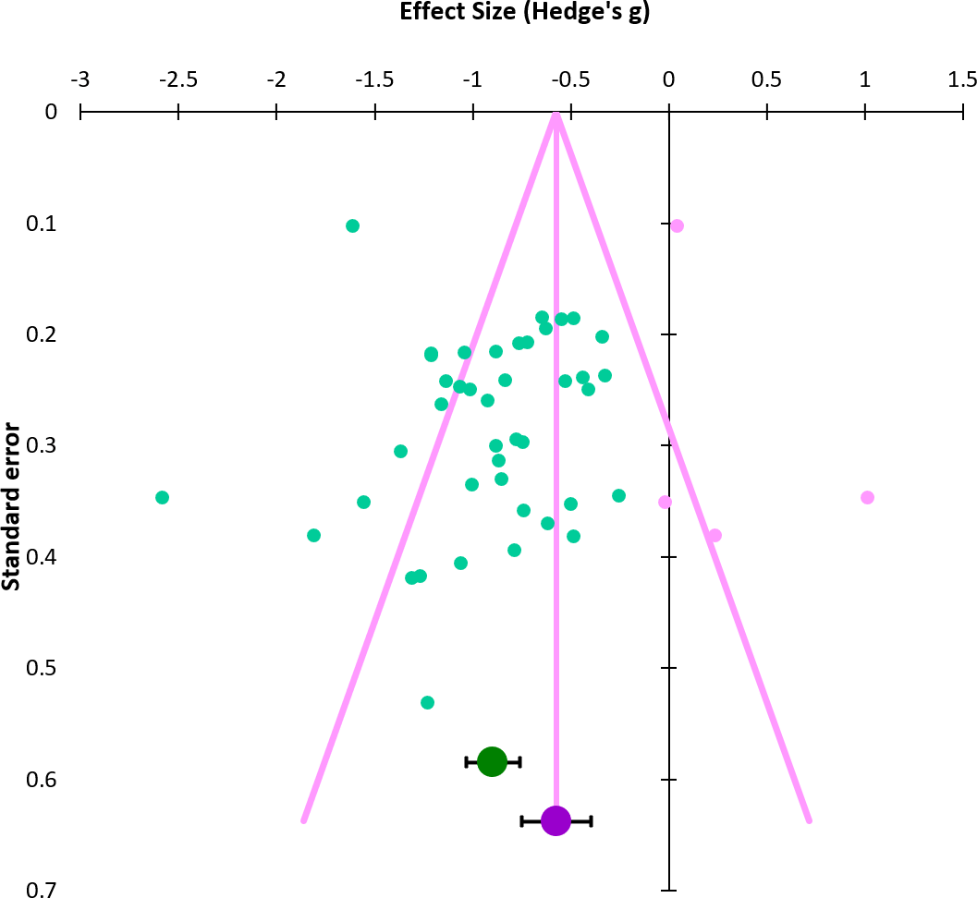


Supplementary Figure 6. Funnel plot of individual results included in primary meta-analysis.

*Aqua*, included results (n = 42); *pink,* imputed results (n = 4); *green*, observed effect size [CI]; *purple*, adjusted effect size [CI].


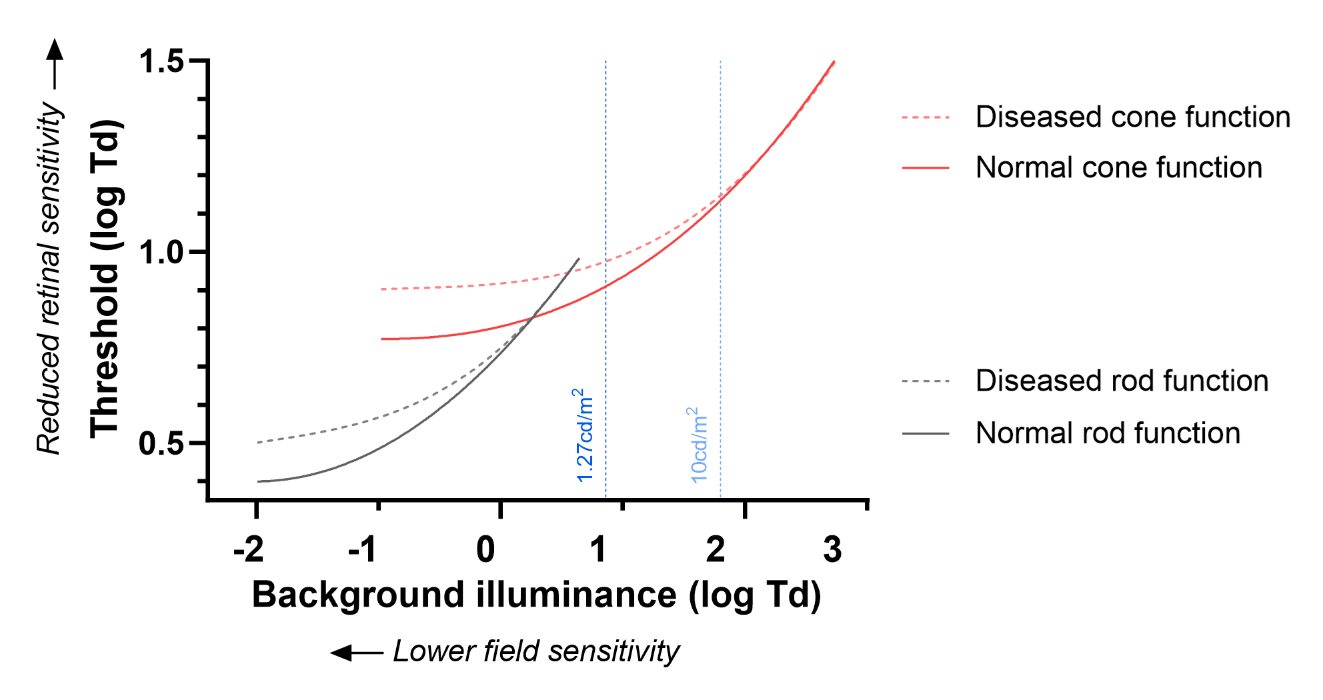


**Supplementary Figure 7. Threshold versus Intensity curve model for photoreceptor disease.**

Derived from Simunovic et al’s(198) automated perimetry data and predicted cone/rod (50%) function loss. An undilated 3mm diameter pupil was assumed for calculations. Model for photoreceptor disease represented reduced quantal catch and number of functional photoreceptors.(199)

Note the ‘upward and rightward’ shift of the TVI curve following cone/rod loss (*dotted lines*), as retinal sensitivity and background field sensitivity are both affected due to ‘spectral filter effects’ of photoreceptor disease. The sensitivity difference from the shift is minimal at higher light levels (e.g., photopic 10cd/m^2^ in SAP), compared to lower light levels (e.g., mesopic 1.27cd/m^2^ as seen in most microperimeters). Thus, retinal sensitivity impairment in photoreceptor diseases is proposed to be light-adaptation dependent.

Abbreviation: Td, Trolands = cd/m^2^ × mm^2^

# Supplementary References

1. Rohatgi A. Webplotdigitizer - https://automeris.io/webplotdigitizer [Internet]. Pacifica, California, USA; 2022. Available from: https://automeris.io/WebPlotDigitizer

2. Higgins J, Li T, Deeks J. Chapter 6: choosing effect measures and computing estimates of effect [Internet]. 2022 [cited 2022 May 8]. Available from: https://training.cochrane.org/handbook/current/chapter-06

3. Kwak SG, Kim JH. Central limit theorem: the cornerstone of modern statistics. Korean J Anesthesiol. 2017 Apr;70(2):144–56.

4. Lin L, Aloe AM. Evaluation of various estimators for standardized mean difference in meta-analysis. Stat Med. 2021 Jan 30;40(2):403–26.

5. Brydges CR. Effect size guidelines, sample size calculations, and statistical power in gerontology. Innov Aging. 2019 Sep 4;3(4):igz036.

6. Sullivan GM, Feinn R. Using effect size—or why the p value is not enough. J Grad Med Educ. 2012 Sep;4(3):279–82.

7. Cohen J. Statistical power analysis for the behavioural sciences [Internet]. 2nd ed. New York: Lawrence Erlbaum Associates; 1988. Available from: https://www.taylorfrancis.com/books/mono/10.4324/9780203771587/statistical-power-analysis-behavioral-sciences-jacob-cohen

8. Borenstein M, Hedges LV, Higgins JPT, Rothstein HR. Introduction to meta-analysis [Internet]. John Wiley and Sons; 2009 [cited 2021 Nov 10]. Available from: http://www.scopus.com/inward/record.url?scp=84889351499&partnerID=8YFLogxK

9. Borenstein M, Higgins JPT, Hedges LV, Rothstein HR. Basics of meta-analysis: i2 is not an absolute measure of heterogeneity. Res Synth Methods. 2017 Mar;8(1):5–18.

10. Higgins JPT, Thompson SG, Deeks JJ, Altman DG. Measuring inconsistency in meta-analyses. BMJ. 2003 Sep 6;327(7414):557–60.

11. Higgins J, Thomas J, Chandler J, Cumpston M, Li T, Page M. Cochrane. 2019 [cited 2021 Feb 22]. Chapter 10: analysing data and undertaking meta-analyses. Available from: https://training.cochrane.org/handbook/current/chapter-10

12. Schober P, Boer C, Schwarte LA. Correlation coefficients: appropriate use and interpretation. Anesth Analg. 2018 May;126(5):1763–8.

13. Acton JH, Gibson JM, Cubbidge RP. Quantification of visual field loss in age-related macular degeneration. PLOS ONE. 2012;7(6):e39944.

14. Acton JH, Greenstein VC. Fundus-driven perimetry (microperimetry) compared to conventional static automated perimetry: similarities, differences, and clinical applications. Can J Ophthalmol. 2013 Oct;48(5):358–63.

15. Acton JH, Smith RT, Hood DC, Greenstein VC. Relationship between retinal layer thickness and the visual field in early age-related macular degeneration. Invest Ophthalmol Vis Sci. 2012 Nov 9;53(12):7618–24.

16. Anderson AJ, Johnson CA, Werner JS. Measuring visual function in age-related macular degeneration with frequency-doubling (matrix) perimetry. Optom Vis Sci. 2011 Jul;88(7):806–15.

17. Atchison DA, Lovie-Kitchin JE, Swann PG. Investigation of central visual fields in patients with age-related macular changes. Optom Vis Sci. 1990 Mar;67(3):179–83.

18. Barboni MTS, Szepessy Z, Ventura DF, Németh J. Individual test point fluctuations of macular sensitivity in healthy eyes and eyes with age-related macular degeneration measured with microperimetry. Transl Vis Sci Technol. 2018 Apr;7(2):25.

19. Birch DG, Toler SM, Swanson WH, Fish GE, Laties AM. A double-blind placebo-controlled evaluation of the acute effects of sildenafil citrate (viagra) on visual function in subjects with early-stage age-related macular degeneration. Am J Ophthalmol. 2002 May;133(5):665–72.

20. Cassels NK, Wild JM, Margrain TH, Chong V, Acton JH. The use of microperimetry in assessing visual function in age-related macular degeneration. Surv Ophthalmol. 2018 Feb;63(1):40–55.

21. Chen JC, Fitzke FW, Pauleikhoff D, Bird AC. Functional loss in age-related Bruch’s membrane change with choroidal perfusion defect. Invest Ophthalmol Vis Sci. 1992 Feb;33(2):334–40.

22. Cheng AS, Vingrys AJ. Visual losses in early age-related maculopathy. Optom Vis Sci Off Publ Am Acad Optom. 1993 Feb;70(2):89–96.

23. Choi AYJ, Nivison-Smith L, Phu J, Zangerl B, Khuu SK, Jones BW, et al. Contrast sensitivity isocontours of the central visual field. Sci Rep. 2019 Aug 12;9(1):11603.

24. Denniss J, Baggaley HC, Brown GM, Rubin GS, Astle AT. Properties of visual field defects around the monocular preferred retinal locus in age-related macular degeneration. Invest Ophthalmol Vis Sci. 2017 May 1;58(5):2652–8.

25. Denniss J, Baggaley HC, Astle AT. Predicting visual acuity from visual field sensitivity in age-related macular degeneration. Invest Ophthalmol Vis Sci. 2018 Sep 4;59(11):4590–7.

26. Eisner A, Klein ML, Zilis JD, Watkins MD. Visual function and the subsequent development of exudative age-related macular degeneration. Invest Ophthalmol Vis Sci. 1992 Oct;33(11):3091–102.

27. Feher J, Kovacs B, Kovacs I, Schveoller M, Papale A, Balacco Gabrieli C. Improvement of visual functions and fundus alterations in early age-related macular degeneration treated with a combination of acetyl-L-carnitine, n-3 fatty acids, and coenzyme Q10. Ophthalmol J Int Ophtalmol Int J Ophthalmol Z Augenheilkd. 2005 Jun;219(3):154–66.

28. Feigl B, Brown B, Lovie-Kitchin J, Swann P. Cone-mediated multifocal electroretinogram in early age-related maculopathy and its relationships with subjective macular function tests. Curr Eye Res. 2004 Jan;29(4–5):327–36.

29. Feigl B, Brown B, Lovie-Kitchin J, Swann P. Monitoring retinal function in early age-related maculopathy: visual performance after 1 year. Eye. 2005 Nov;19(11):1169–77.

30. Forshaw T, Parpounas A, Sorensen T. Correlation of macular sensitivity measures and visual acuity to vision-related quality of life in patients with age-related macular degeneration. BMC Ophthalmol. 2021 Mar 23;21(1).

31. Forte R, Cennamo G, de Crecchio G, Cennamo G. Microperimetry of subretinal drusenoid deposits. Ophthalmic Res. 2014;51(1):32–6.

32. Fraser RG, Tan R, Ayton LN, Caruso E, Guymer RH, Luu CD. Assessment of retinotopic rod photoreceptor function using a dark-adapted chromatic perimeter in intermediate age-related macular degeneration. Invest Ophthalmol Vis Sci. 2016 Oct 1;57(13):5436–42.

33. Frennesson C, Nilsson UL, Nilsson SE. Colour contrast sensitivity in patients with soft drusen, an early stage of ARM. Doc Ophthalmol Adv Ophthalmol. 1995;90(4):377–86.

34. Gunawan JR, Thiele SH, Isselmann B, Caruso E, Guymer RH, Luu CD. Effect of subthreshold nanosecond laser on retinal structure and function in intermediate age-related macular degeneration. Clin Experiment Ophthalmol. 2022 Jan;50(1):31–9.

35. Guymer RH, Gross-Jendroska M, Owens SL, Bird AC, Fitzke FW. Laser treatment in subjects with high-risk clinical features of age-related macular degeneration. posterior pole appearance and retinal function. Arch Ophthalmol. 1997 May;115(5):595–603.

36. Hartmann KI, Bartsch DUG, Cheng L, Kim JS, Gomez ML, Klein H, et al. Scanning laser ophthalmoscope imaging stabilized microperimetry in dry age-related macular degeneration. Retina Phila Pa. 2011 Aug;31(7):1323–31.

37. Hassan SE, Lovie-Kitchin JE, Woods RL. Vision and mobility performance of subjects with age-related macular degeneration. Optom Vis Sci. 2002 Nov;79(11):697–707.

38. Hazel CA, Petre KL, Armstrong RA, Benson MT, Frost NA. Visual function and subjective quality of life compared in subjects with acquired macular disease. Invest Ophthalmol Vis Sci. 2000 May;41(6):1309–15.

39. Herse P. An application of threshold-versus-intensity functions in automated static perimetry. Vision Res. 2005 Feb 1;45(4):461–8.

40. Kaiser HJ, Flammer J, Bucher PJ, De Natale R, Stümpfig D, Hendrickson P. High-resolution perimetry of the central visual field. Ophthalmologica. 1994;208(1):10–4.

41. Karadeniz Ugurlu S, Kocakaya Altundal AE, Altin Ekin M. Comparison of vision-related quality of life in primary open-angle glaucoma and dry-type age-related macular degeneration. Eye. 2017 Mar;31(3):395–405.

42. Kim DY, Yang HS, Kook YJ, Lee JY. Association between microperimetric parameters and optical coherent tomographic findings in various macular diseases. Korean J Ophthalmol KJO. 2015 Apr;29(2):92–101.

43. Kitano M, Fujita A, Asaoka R, Inoue T, Amari T, Komatsu K, et al. Assessment of macular function in patients with non-vascularized pigment epithelial detachment. Sci Rep. 2021 Aug 16;11(1):16577.

44. Laishram M., Srikanth K., Adityapuram R.R., Nagarajan S., Govindasamy E. Microperimetry - a new tool for assessing retinal sensitivity in macular diseases. J Clin Diagn Res. 2017;11(7):NC08-NC11.

45. Landa G, Su E, Garcia PMT, Seiple WH, Rosen RB. Inner segment-outer segment junctional layer integrity and corresponding retinal sensitivity in dry and wet forms of age-related macular degeneration. Retina Phila Pa. 2011 Feb;31(2):364–70.

46. Lee HJ, Kim MS, Jo YJ, Kim JY. Ganglion cell–inner plexiform layer thickness in retinal diseases: repeatability study of spectral-domain optical coherence tomography. Am J Ophthalmol. 2015 Aug;160(2):283-289.e1.

47. Limoli P.G., Vingolo E.M., Morales M.U., Nebbioso M., Limoli C. Preliminary study on electrophysiological changes after cellular autograft in age-related macular degeneration. Med U S. 2014;93(29):e355.

48. Liu H, Bittencourt MG, Wang J, Sepah YJ, Ibrahim-Ahmed M, Rentiya Z, et al. Retinal sensitivity is a valuable complementary measurement to visual acuity--a microperimetry study in patients with maculopathies. Graefes Arch Clin Exp Ophthalmol Albrecht Von Graefes Arch Klin Exp Ophthalmol. 2015 Dec;253(12):2137–42.

49. Liu H, Xu Y, Wong D, Yow AP, Laude A, Lim TH. Detecting impaired vision caused by AMD from gaze data. Annu Int Conf IEEE Eng Med Biol Soc. 2017 Jul;2017:3142–5.

50. Markowitz SN, Devenyi RG, Munk MR, Croissant CL, Tedford SE, Rückert R, et al. A Double-Masked, Randomized, Sham-Controlled, Single-Center Study with Photobiomodulation for the Treatment of Dry Age-Related Macular Degeneration. Retina Phila Pa. 2020 Aug;40(8):1471–82.

51. Mayer MJ, Spiegler SJ, Ward B, Glucs A, Kim CB. Foveal flicker sensitivity discriminates arm-risk from healthy eyes. Invest Ophthalmol Vis Sci. 1992 Oct 1;33(11):3143–9.

52. Mazzoli LS, Urata CN, Kasahara N. Face memory deficits in subjects with eye diseases: a comparative analysis between glaucoma and age-related macular degeneration patients from a developing country. Graefes Arch Clin Exp Ophthalmol Albrecht Von Graefes Arch Klin Exp Ophthalmol. 2019 Sep;257(9):1941–6.

53. McGuinness MB, Fraser RG, Tan R, Luu CD, Guymer RH. Relationship Between Rod-Mediated Sensitivity, Low-Luminance Visual Acuity, and Night Vision Questionnaire in Age-Related Macular Degeneration. Transl Vis Sci Technol. 2020 May;9(6):30.

54. Midena E, Segato T, Blarzino MC, Angeli CD. Macular drusen and the sensitivity of the central visual field. Doc Ophthalmol. 1994 Jun 1;88(2):179–85.

55. Midena E, Degli Angeli C, Blarzino MC, Valenti M, Segato T. Macular function impairment in eyes with early age-related macular degeneration. Invest Ophthalmol Vis Sci. 1997 Feb;38(2):469–77.

56. Midena E, Pilotto E. Microperimetry in age: related macular degeneration. Eye Lond Engl. 2017 Jul;31(7):985–94.

57. Narayanan D., Wallstrom G., Rodriguez J., Welch D., Chapin M., Arrigg P., et al. Early ophthalmic changes in macula does not correlate with visual function. Clin Ophthalmol. 2020;14((Narayanan, Rodriguez, Welch, Chapin, Patil, Abelson) Ora, Inc, Andover, MA, United States):2571–6.

58. Nebbioso M, Barbato A, Pescosolido N. Scotopic Microperimetry in the Early Diagnosis of Age-Related Macular Degeneration: Preliminary Study. BioMed Res Int [Internet]. 2014 [cited 2019 Jun 15]; Available from: https://www.hindawi.com/journals/bmri/2014/671529/

59. Neelam K, Nolan J, Chakravarthy U, Beatty S. Psychophysical Function in Age-related Maculopathy. Surv Ophthalmol. 2009 Mar 1;54(2):167–210.

60. Neely D, Zarubina AV, Clark ME, Huisingh CE, Jackson GR, Zhang Y, et al. Association between visual function and subretinal drusenoid deposits in normal and early age-related macular degeneration eyes. Retina. 2017 Jul;37(7):1329–36.

61. Nguyen CT, Fraser RG, Tan R, Caruso E, Lek JJ, Guymer RH, et al. Longitudinal Changes in Retinotopic Rod Function in Intermediate Age-Related Macular Degeneration. Invest Ophthalmol Vis Sci. 2018 Mar 20;59(4):AMD19–24.

62. Owsley C, Huisingh C, Clark ME, Jackson GR, McGwin G. Comparison of visual function in older eyes in the earliest stages of age-related macular degeneration to those in normal macular health. Curr Eye Res. 2016;41(2):266–72.

63. Owsley C, Clark ME, Huisingh CE, Curcio CA, McGwin G. Visual function in older eyes in normal macular health: association with incident early age-related macular degeneration 3 years later. Invest Ophthalmol Vis Sci. 2016 Apr;57(4):1782–9.

64. Pfau M, Jolly JK, Wu Z, Denniss J, Lad EM, Guymer RH, et al. Fundus-controlled perimetry (microperimetry): Application as outcome measure in clinical trials. Prog Retin Eye Res. 2021 May 1;82:100907.

65. Phipps JA, Guymer RH, Vingrys AJ. Loss of cone function in age-related maculopathy. Invest Ophthalmol Vis Sci. 2003 May 1;44(5):2277–83.

66. Piccardi M, Ziccardi L, Stifano G, Montrone L, Iarossi G, Minnella A, et al. Regional Cone-Mediated Dysfunction in Age-Related Maculopathy Evaluated by Focal Electroretinograms: Relationship with Retinal Morphology and Perimetric Sensitivity. Ophthalmic Res. 2009;41(4):194–202.

67. Querques L, Querques G, Forte R, Souied EH. Microperimetric correlations of autofluorescence and optical coherence tomography imaging in dry age-related macular degeneration. Am J Ophthalmol. 2012 Jun;153(6):1110–5.

68. Querques G, Massamba N, Srour M, Boulanger E, Georges A, Souied EH. Impact of reticular pseudodrusen on macular function. Retina Phila Pa. 2014 Feb;34(2):321–9.

69. Rai BB, Essex RW, Sabeti F, Maddess T, Rohan EMF, van Kleef JP, et al. An Objective Perimetry Study of Central Versus Peripheral Sensitivities and Delays in Age-Related Macular Degeneration. Transl Vis Sci Technol. 2021 Dec 21;10(14):24.

70. Rovner BW, Casten RJ, Hegel MT, Tasman WS. Minimal depression and vision function in age-related macular degeneration. Ophthalmology. 2006 Oct;113(10):1743–7.

71. Sabeti F., Lane J., Rohan E.M.F., Essex R.W., McKone E., Maddess T. Relationships between retinal structure and function and vision-related quality of life measures in advanced age-related macular degeneration. Graefes Arch Clin Exp Ophthalmol. 2021;259(12):3687–96.

72. Sandberg MA, Weiner A, Miller S, Gaudio AR. High-risk characteristics of fellow eyes of patients with unilateral neovascular age-related macular degeneration11The authors have no proprietary interest in the products described in this article. Ophthalmology. 1998 Mar 1;105(3):441–7.

73. Scorolli L, Corazza D, Morara M, Vismara S, Lugaresi ML, Meduri RA. Argon laser vs. subthreshold infrared (810-nm) diode laser macular grid photocoagulation in nonexudative age-related macular degeneration. Can J Ophthalmol J Can Ophtalmol. 2003 Oct;38(6):489–95.

74. Shinojima A, Sawa M, Mori R, Sekiryu T, Oshima Y, Kato A, et al. Five-year follow-up of fundus autofluorescence and retinal sensitivity in the fellow eye in exudative age-related macular degeneration in Japan. PloS One. 2020;15(3):e0229694.

75. Steinberg JS, Fitzke FW, Fimmers R, Fleckenstein M, Holz FG, Schmitz-Valckenberg S. Scotopic and Photopic Microperimetry in Patients With Reticular Drusen and Age-Related Macular Degeneration. JAMA Ophthalmol. 2015 Jun;133(6):690–7.

76. Steinberg JS, Saßmannshausen M, Pfau M, Fleckenstein M, Finger RP, Holz FG, et al. Evaluation of Two Systems for Fundus-Controlled Scotopic and Mesopic Perimetry in Eye with Age-Related Macular Degeneration. Transl Vis Sci Technol. 2017 Jul;6(4):7.

77. Sunness JS, Johnson MA, Massof RW, Marcus S. Retinal sensitivity over drusen and nondrusen areas. A study using fundus perimetry. Arch Ophthalmol Chic Ill 1960. 1988 Aug;106(8):1081–4.

78. Tan RS, Guymer RH, Aung KZ, Caruso E, Luu CD. Longitudinal assessment of rod function in intermediate age-related macular degeneration with and without reticular pseudodrusen. Invest Ophthalmol Vis Sci. 2019 Apr 17;60(5):1511–8.

79. Tolentino MJ, Miller S, Gaudio AR, Sandberg MA. Visual field deficits in early age-related macular degeneration. Vision Res. 1994 Feb;34(3):409–13.

80. Tran THC, Rambaud C, Despretz P, Boucart M. Scene perception in age-related macular degeneration. Invest Ophthalmol Vis Sci. 2010 Dec;51(12):6868–74.

81. Tran THC, Despretz P, Boucart M. Scene perception in age-related macular degeneration: the effect of contrast. Optom Vis Sci. 2012 Apr;89(4):419–25.

82. Tran BK, Herbort CPJ. Discrepancy between visual acuity and microperimetry in AMD patients: visual acuity appears as an inadequate parameter to test macular function. Klin Monatsbl Augenheilkd. 2015 Apr;232(4):529–32.

83. Vessey KA, Ho T, Jobling AI, Mills SA, Tran MX, Brandli A, et al. Nanosecond laser treatment for age-related macular degeneration does not induce focal vision loss or new vessel growth in the retina. Invest Ophthalmol Vis Sci. 2018 Feb 1;59(2):731–45.

84. Wang MY, Rousseau J, Boisjoly H, Schmaltz H, Kergoat MJ, Moghadaszadeh S, et al. Activity Limitation due to a Fear of Falling in Older Adults with Eye Disease. Invest Ophthalmol Vis Sci. 2012 Dec 1;53(13):7967–72.

85. Weigert G, Kaya S, Pemp B, Sacu S, Lasta M, Werkmeister RM, et al. Effects of lutein supplementation on macular pigment optical density and visual acuity in patients with age-related macular degeneration. Invest Ophthalmol Vis Sci. 2011 Oct 17;52(11):8174–8.

86. White UE, Black AA, Delbaere K, Wood JM. Determinants of concern about falling in adults with age-related macular degeneration. Ophthalmic Physiol Opt.

87. White UE, Black AA, Delbaere K, Wood JM. Longitudinal Impact of Vision Impairment on Concern About Falling in People With Age-Related Macular Degeneration. Transl Vis Sci Technol. 2022 Jan 3;11(1):34.

88. Wong EN, Chew AL, Morgan WH, Patel PJ, Chen FK. The Use of Microperimetry to Detect Functional Progression in Non-Neovascular Age-Related Macular Degeneration: A Systematic Review. Asia-Pac J Ophthalmol Phila Pa. 2017 Feb;6(1):70–9.

89. Wood JM, Lacherez P, Black AA, Cole MH, Boon MY, Kerr GK. Risk of falls, injurious falls, and other injuries resulting from visual impairment among older adults with age-related macular degeneration. Invest Ophthalmol Vis Sci. 2011 Jul 7;52(8):5088–92.

90. Wood JM, Black AA, Mallon K, Kwan AS, Owsley C. Effects of Age-Related Macular Degeneration on Driving Performance. Invest Ophthalmol Vis Sci. 2018 01;59(1):273–9.

91. Yanagisawa M., Kato S., Ochiai M. Comparison of esterman disability scores obtained using Goldmann perimetry and the Humphrey field analyzer in japanese low-vision patients. PLOS ONE. 2018;13(9):e0203258.

92. Yang Y, Dunbar H. Clinical Perspectives and Trends: Microperimetry as a Trial Endpoint in Retinal Disease. Ophthalmol J Int Ophtalmol Int J Ophthalmol Z Augenheilkd. 2021;244(5):418–50.

93. Yow AP, Wong D, Liu H, Zhu H, Ong IJW, Laude A, et al. Automatic visual impairment detection system for age-related eye diseases through gaze analysis. Annu Int Conf IEEE Eng Med Biol Soc. 2017 Jul;2017:2450–3.

94. Centre for Evidence Based Medicine. Study designs — centre for evidence-based medicine (cebm), university of oxford [Internet]. 2022 [cited 2022 May 17]. Available from: https://www.cebm.ox.ac.uk/resources/ebm-tools/study-designs

95. Corvi F., Pellegrini M., Belotti M., Bianchi C., Staurenghi G. Scotopic and fast mesopic microperimetry in eyes with drusen and reticular pseudodrusen. Retina. 2019;39(12):2378–83.

96. Forte R, Cennamo G, de Crecchio G, Cennamo G. Microperimetry of subretinal drusenoid deposits. Ophthalmic Res. 2014;51(1):32–6.

97. Huang YM, Dou HL, Huang FF, Xu XR, Zou ZY, Lu XR, et al. Changes following supplementation with lutein and zeaxanthin in retinal function in eyes with early age-related macular degeneration: a randomised, double-blind, placebo-controlled trial. Br J Ophthalmol. 2015 Mar;99(3):371–5.

98. McGuinness M.B., Finger R.P., Wu Z., Luu C.D., Chen F.K., Arnold J.J., et al. Properties of the impact of vision impairment and night vision questionnaires among people with intermediate age-related macular degeneration. Transl Vis Sci Technol. 2019;8(5):3.

99. McGuinness M.B., Finger R.P., Wu Z., Luu C.D., Chen F.K., Arnold J.J., et al. Association between patient-reported outcomes and time to late age-related macular degeneration in the laser intervention in early stages of age-related macular degeneration study. Ophthalmol Retina. 2020;4(9):881–8.

100. Sassmannshausen M, Pfau M, Thiele S, Fimmers R, Steinberg JS, Fleckenstein M, et al. Longitudinal analysis of structural and functional changes in presence of reticular pseudodrusen associated with age-related macular degeneration. Invest Ophthalmol Vis Sci. 2020 Aug 3;61(10):19.

101. Steinberg J.S., Sassmannshausen M., Fleckenstein M., Fimmers R., Oishi A., Holz F.G., et al. Correlation of partial outer retinal thickness with scotopic and mesopic fundus-controlled perimetry in patients with reticular drusen. Am J Ophthalmol. 2016;168((Steinberg, Sasmannshausen, Fleckenstein, Oishi, Holz, Schmitz-Valckenberg) Department of Ophthalmology, University of Bonn, Ernst-Abbe-Str 2, Bonn 53127, Germany):52–61.

102. Tepelus T.C., Hariri A.H., Al-Sheikh M., Sadda S.R. Correlation between mesopic retinal sensitivity and optical coherence tomographic metrics of the outer retina in patients with non-atrophic dry age-related macular degeneration. Ophthalmic Surg Lasers Imaging. 2017;48(4):312–8.

103. Dow CT, Harley CB. Evaluation of an oral telomerase activator for early age-related macular degeneration - a pilot study. Clin Ophthalmol. 2016;10:243–9.

104. Guymer RH, Brassington KH, Dimitrov P, Makeyeva G, Plunkett M, Xia W, et al. Nanosecond-laser application in intermediate AMD: 12-month results of fundus appearance and macular function. Clin Experiment Ophthalmol. 2014 Jul;42(5):466–79.

105. Prea SM, Kong GYX, Guymer RH, Sivarajah P, Baglin EK, Vingrys AJ. The short-term compliance and concordance to in clinic testing for tablet-based home monitoring in age-related macular degeneration. Am J Ophthalmol. 2022 Mar;235:280–90.

106. Querques G, Sacconi R, Gelormini F, Borrelli E, Prascina F, Zucchiatti I, et al. Subthreshold laser treatment for reticular pseudodrusen secondary to age-related macular degeneration. Sci Rep. 2021 Jan 26;11(1):2193.

107. Taylor DJ, Smith ND, Jones PR, Binns AM, Crabb DP. Measuring dynamic levels of self-perceived anxiety and concern during simulated mobility tasks in people with non-neovascular age-related macular degeneration. Br J Ophthalmol. 2020 Apr;104(4):529–34.

108. Acton JH, Bartlett NS, Greenstein VC. Comparing the Nidek MP-1 and Humphrey Field Analyzer in Normal Subjects. Optom Vis Sci. 2011 Nov;88(11):1288–97.

109. Bird AC, Bressler NM, Bressler SB, Chisholm IH, Coscas G, Davis MD, et al. An international classification and grading system for age-related maculopathy and age-related macular degeneration. Surv Ophthalmol. 1995 Mar 1;39(5):367–74.

110. Adams M., Ho C.Y.D., Baglin E., Sharangan P., Wu Z., Lawson D.J., et al. Home monitoring of retinal sensitivity on a tablet device in intermediate age-related macular degeneration. Transl Vis Sci Technol. 2018;7(5):32.

111. Ferris FL, Wilkinson CP, Bird A, Chakravarthy U, Chew E, Csaky K, et al. Clinical classification of age-related macular degeneration. Ophthalmology. 2013 Apr;120(4):844–51.

112. Broadhead GK, Hong T, McCluskey P, Grigg JR, Schlub TE, Chang AA. Choroidal Thickness and Microperimetry Sensitivity in Age-Related Macular Degeneration. Ophthalmic Res. 2017;58(1):27–34.

113. Age-Related Eye Disease Study Research Group. The age-related eye disease study system for classifying age-related macular degeneration from stereoscopic color fundus photographs: the age-related eye disease study report number 6. Am J Ophthalmol. 2001 Nov;132(5):668–81.

114. Broadhead GK, Hong T, Grigg JR, McCluskey P, Schlub TE, Spooner K, et al. Does functional assessment predict everyday visual functioning? Visual function testing and quality of life in mild/moderate age-related macular degeneration. Int Ophthalmol. 2020 Jul 14;

115. Chandramohan A, Stinnett SS, Petrowski JT, Schuman SG, Toth CA, Cousins SW, et al. Visual function measures in early and intermediate age-related macular degeneration. Retina. 2016 May;36(5):1021–31.

116. Chen C, Wu L, Wu D, Huang S, Wen F, Luo G, et al. The local cone and rod system function in early age-related macular degeneration. Doc Ophthalmol Adv Ophthalmol. 2004 Jul;109(1):1–8.

117. Clemens CR, Alten F, Heiduschka P, Eter N. Morphology score as a marker of retinal function in drusenoid pigment epithelial detachment. Retina. 2015;35(7):1351–9.

118. Cocce KJ, Stinnett SS, Luhmann UFO, Vajzovic L, Horne A, Schuman SG, et al. Visual Function Metrics in Early and Intermediate Dry Age-related Macular Degeneration for Use as Clinical Trial Endpoints. Am J Ophthalmol. 2018 May;189:127–38.

119. Corvi F, Pellegrini M, Belotti M, Bianchi C, Staurenghi G. Scotopic and fast mesopic microperimetry in eyes with drusen and reticular pseudodrusen. Retina. 2019;39(12):2378–83.

120. Dinc UA, Yenerel M, Gorgun E, Oncel M. Assessment of macular function by microperimetry in intermediate age-related macular degeneration. Eur J Ophthalmol. 2008 Aug;18(4):595–600.

121. Echols BS, Clark ME, Swain TA, Chen L, Kar D, Zhang Y, et al. Hyperreflective foci and specks are associated with delayed rod-mediated dark adaptation in nonneovascular age-related macular degeneration. Ophthalmol Retina. 2020 Nov;4(11):1059–68.

122. Davis MD, Gangnon RE, Lee LY, Hubbard LD, Klein BEK, Klein R, et al. The age-related eye disease study severity scale for age-related macular degeneration: AREDS report no. 17. Arch Ophthalmol. 2005 Nov;123(11):1484–98.

123. Fragiotta S, Carnevale C, Cutini A, Vingolo EM. Correlation between retinal function and microstructural foveal changes in intermediate age-related macular degeneration. Int J Retina Vitr. 2017;3:8.

124. Fragiotta S, Costanzo E, Viggiano P, De Geronimo D, Scuderi G, Varano M, et al. Functional Correlates of Outer Retina Remodeling in Intermediate Age-Related Macular Degeneration Using Microperimetry. Invest Ophthalmol Vis Sci. 2022 Mar 2;63(3):16.

125. Gin TJ, Luu CD, Guymer RH. Central Retinal Function as Measured by the Multifocal Electroretinogram and Flicker Perimetry in Early Age-Related Macular Degeneration. Invest Ophthalmol Vis Sci. 2011 Nov 1;52(12):9267–74.

126. Goh KL, Abbott CJ, Hadoux X, Jannaud M, Hodgson LAB, van Wijngaarden P, et al. Hyporeflective cores within drusen: association with progression of age-related macular degeneration and impact on visual sensitivity. Ophthalmol Retina. 2022 Apr;6(4):284–90.

127. Lek JJ, Brassington KH, Luu CD, Chen FK, Arnold JJ, Heriot WJ, et al. Subthreshold nanosecond laser intervention in intermediate age-related macular degeneration: study design and baseline characteristics of the laser in early stages of age-related macular degeneration study (report number 1). Ophthalmol Retina. 2017 Jun;1(3):227–39.

128. Guymer RH, Wu Z, Hodgson LAB, Caruso E, Brassington KH, Tindill N, et al. Subthreshold nanosecond laser intervention in age-related macular degeneration: the lead randomized controlled clinical trial. Ophthalmology. 2019 Jun;126(6):829–38.

129. Grewal MK, Chandra S, Bird A, Jeffery G, Sivaprasad S. Scotopic thresholds on dark-adapted chromatic perimetry in healthy aging and age-related macular degeneration. Sci Rep. 2021 May 14;11(1):10349.

130. Guymer RH, Tan RS, Luu CD. Comparison of Visual Function Tests in Intermediate Age-Related Macular Degeneration. Transl Vis Sci Technol. 2021 Oct 4;10(12):14.

131. Haimovici R, Owens SL, Fitzke FW, Bird AC. Dark adaptation in age-related macular degeneration: relationship to the fellow eye. Graefes Arch Clin Exp Ophthalmol. 2002 Feb;240(2):90–5.

132. Higgins B, Taylor D, Bi W, Binns A, Crabb D. Novel computer-based assessments of everyday visual function in people with age-related macular degeneration. PLOS ONE. 2020 Dec 7;15(12).

133. Hsu ST, Thompson AC, Stinnett SS, Luhmann UFO, Vajzovic L, Horne A, et al. Longitudinal Study of Visual Function in Dry Age-Related Macular Degeneration at 12 Months. Ophthalmol Retina. 2019 Aug;3(8):637–48.

134. Huang YM, Dou HL, Huang FF, Xu XR, Zou ZY, Lu XR, et al. Changes following supplementation with lutein and zeaxanthin in retinal function in eyes with early age-related macular degeneration: a randomised, double-blind, placebo-controlled trial. Br J Ophthalmol. 2015 Mar;99(3):371–5.

135. Iwama D, Tsujikawa A, Ojima Y, Nakanishi H, Yamashiro K, Tamura H, et al. Relationship between retinal sensitivity and morphologic changes in eyes with confluent soft drusen. Clin Experiment Ophthalmol. 2010 Jul;38(5):483–8.

136. Kar D, Clark ME, Swain TA, McGwin G Jr, Crosson JN, Owsley C, et al. Local abundance of macular xanthophyll pigment is associated with rod- and cone-mediated vision in aging and age-related macular degeneration. Invest Ophthalmol Vis Sci. 2020 Jul 30;61(8):46.

137. Leal C, De Bats F, Morales M, Decullier E, Denis P, Amoaku W, et al. Anatomical-functional concordance of microperimetry and the simplified age-related macular degeneration study classification: A pilot study. Eur J Ophthalmol. 2022 Jan;32(1):402–9.

138. Luu CD, Dimitrov PN, Robman L, Varsamidis M, Makeyeva G, Aung KZ, et al. Role of Flicker Perimetry in Predicting Onset of Late-Stage Age-Related Macular Degeneration. Arch Ophthalmol. 2012 Jun 1;130(6):690–9.

139. Luu CD, Dimitrov PN, Wu Z, Ayton LN, Makeyeva G, Aung KZ, et al. Static and Flicker Perimetry in Age-Related Macular Degeneration. Invest Ophthalmol Vis Sci. 2013 May 1;54(5):3560–8.

140. Maynard ML, Zele AJ, Feigl B. Mesopic Pelli-Robson contrast sensitivity and MP-1 microperimetry in healthy ageing and age-related macular degeneration. Acta Ophthalmol (Copenh). 2016 Dec;94(8):e772–8.

141. McGuinness MB, Finger RP, Wu Z, Luu CD, Chen FK, Arnold JJ, et al. Properties of the Impact of Vision Impairment and Night Vision Questionnaires Among People With Intermediate Age-Related Macular Degeneration. Transl Vis Sci Technol. 2019 Sep 3;8(5):3–3.

142. McGuinness MB, Finger RP, Wu Z, Luu CD, Guymer RH, Chen FK, et al. Association between Patient-Reported Outcomes and Time to Late Age-Related Macular Degeneration in the Laser Intervention in Early Stages of Age-Related Macular Degeneration Study. Ophthalmol Retina. 2020;4(9):881–8.

143. Messenio D, Babbi A, Guglielmi A, Airaldi M. Focal electroretinogram and microperimetry testing of photoreceptor-retinal pigment epithelium function in intermediate age-related macular degeneration. Acta Ophthalmol (Copenh). 2022 May;100(3):277–84.

144. Midena E, Vujosevic S, Convento E, Manfre’ A, Cavarzeran F, Pilotto E. Microperimetry and fundus autofluorescence in patients with early age-related macular degeneration. Br J Ophthalmol. 2007 Nov;91(11):1499–503.

145. Montesano G, Ometto G, Higgins BE, Iester C, Balaskas K, Tufail A, et al. Structure-Function Analysis in Macular Drusen With Mesopic and Scotopic Microperimetry. Transl Vis Sci Technol. 2020 Dec;9(13):43.

146. Nassisi M, Tepelus T, Corradetti G, Sadda SR. Relationship Between Choriocapillaris Flow and Scotopic Microperimetry in Early and Intermediate Age-related Macular Degeneration. Am J Ophthalmol. 2021 Feb;222:302–9.

147. Nittala MG, Velaga SB, Hariri A, Pfau M, Birch DG, Haines J, et al. Retinal sensitivity using microperimetry in age-related macular degeneration in an Amish population. Ophthalmic Surg Lasers Imaging. 2019 Sep 1;50(9):e236–41.

148. Ogino K, Tsujikawa A, Yamashiro K, Ooto S, Oishi A, Nakata I, et al. Multimodal evaluation of macular function in age-related macular degeneration. Jpn J Ophthalmol. 2014 Mar;58(2):155–65.

149. Ooto S, Suzuki M, Vongkulsiri S, Sato T, Spaide RF. Multimodal visual function testing in eyes with nonexudative age-related macular degeneration. Retina Phila Pa. 2015 Sep;35(9):1726–34.

150. Owsley C, Jackson GR, Cideciyan AV, Huang Y, Fine SL, Ho AC, et al. Psychophysical evidence for rod vulnerability in age-related macular degeneration. Invest Ophthalmol Vis Sci. 2000 Jan;41(1):267–73.

151. Klein R, Davis MD, Magli YL, Segal P, Klein BE, Hubbard L. The Wisconsin age-related maculopathy grading system. Ophthalmology. 1991 Jul;98(7):1128–34.

152. Parisi V, Perillo L, Tedeschi M, Scassa C, Gallinaro G, Capaldo N, et al. Macular function in eyes with early age-related macular degeneration with or without contralateral late age-related macular degeneration. Retina Phila Pa. 2007 Sep;27(7):879–90.

153. Pfau M, Lindner M, Gliem M, Steinberg JS, Thiele S, Finger RP, et al. Mesopic and dark-adapted two-color fundus-controlled perimetry in patients with cuticular, reticular, and soft drusen. Eye Lond Engl. 2018 Dec;32(12):1819–30.

154. Pfau M, Lindner M, Fleckenstein M, Finger RP, Rubin GS, Harmening WM, et al. Test-retest reliability of scotopic and mesopic fundus-controlled perimetry using a modified MAIA (macular integrity assessment) in normal eyes. Ophthalmologica. 2017;237(1):42–54.

155. Phipps JA, Guymer RH, Vingrys AJ. Temporal sensitivity deficits in patients with high-risk drusen. Aust N Z J Ophthalmol. 1999;27(3–4):265–7.

156. Phipps JA, Dang TM, Vingrys AJ, Guymer RH. Flicker Perimetry Losses in Age-Related Macular Degeneration. Invest Ophthalmol Vis Sci. 2004 Sep 1;45(9):3355–60.

157. Pondorfer SG, Terheyden JH, Heinemann M, Wintergerst MWM, Holz FG, Finger RP. Association of vision-related quality of life with visual function in age-related macular degeneration. Sci Rep. 2019 Oct 25;9(1):15326.

158. Pondorfer SG, Wintergerst MWM, Gorgi Zadeh S, Schultz T, Heinemann M, Holz FG, et al. Association of Visual Function Measures with Drusen Volume in Early Stages of Age-Related Macular Degeneration. Invest Ophthalmol Vis Sci. 2020 Mar 9;61(3):55.

159. Pondorfer SG, Heinemann M, Wintergerst MWM, Pfau M, Strömer AL, Holz FG, et al. Detecting vision loss in intermediate age-related macular degeneration: A comparison of visual function tests. PloS One. 2020;15(4):e0231748.

160. Roh M, Laíns I, Shin HJ, Park DH, Mach S, Vavvas DG, et al. Microperimetry in age-related macular degeneration: association with macular morphology assessed by optical coherence tomography. Br J Ophthalmol. 2019 Dec;103(12):1769–76.

161. Saßmannshausen M, Steinberg JS, Fimmers R, Pfau M, Thiele S, Fleckenstein M, et al. Structure-Function Analysis in Patients With Intermediate Age-Related Macular Degeneration. Invest Ophthalmol Vis Sci. 2018 Mar 1;59(3):1599–608.

162. Sassmannshausen M, Pfau M, Thiele S, Fimmers R, Steinberg JS, Fleckenstein M, et al. Longitudinal Analysis of Structural and Functional Changes in Presence of Reticular Pseudodrusen Associated With Age-Related Macular Degeneration. Invest Ophthalmol Vis Sci. 2020 Aug 3;61(10):19.

163. Saßmannshausen M, Zhou J, Pfau M, Thiele S, Steinberg J, Fleckenstein M, et al. Longitudinal Analysis of Retinal Thickness and Retinal Function in Eyes with Large Drusen Secondary to Intermediate Age-Related Macular Degeneration. Ophthalmol Retina. 2021 Mar 1;5(3):241–50.

164. Steinberg JS, Saßmannshausen M, Fleckenstein M, Fimmers R, Oishi A, Holz FG, et al. Correlation of Partial Outer Retinal Thickness With Scotopic and Mesopic Fundus-Controlled Perimetry in Patients With Reticular Drusen. Am J Ophthalmol. 2016 Aug;168:52–61.

165. Steinmetz RL, Haimovici R, Jubb C, Fitzke FW, Bird AC. Symptomatic abnormalities of dark adaptation in patients with age-related Bruch’s membrane change. Br J Ophthalmol. 1993 Sep;77(9):549–54.

166. Tan R, Guymer RH, Luu CD. Subretinal drusenoid deposits and the loss of rod function in intermediate age-related macular degeneration. Invest Ophthalmol Vis Sci. 2018 Aug 1;59(10):4154–61.

167. Tepelus TC, Hariri AH, Al-Sheikh M, Sadda SR. Correlation Between Mesopic Retinal Sensitivity and Optical Coherence Tomographic Metrics of the Outer Retina in Patients With Non-Atrophic Dry Age-Related Macular Degeneration. Ophthalmic Surg Lasers Imaging Retina. 2017 01;48(4):312–8.

168. Thompson AC, Luhmann UFO, Stinnett SS, Vajzovic L, Horne A, Toth CA, et al. Association of Low Luminance Questionnaire With Objective Functional Measures in Early and Intermediate Age-Related Macular Degeneration. Invest Ophthalmol Vis Sci. 2018 Jan;59(1):289–97.

169. Uddin D., Jeffrey B.G., Flynn O., Wong W., Wiley H., Keenan T., et al. Repeatability of scotopic sensitivity and dark adaptation using a Medmont dark-adapted chromatic perimeter in age-related macular degeneration. Transl Vis Sci Technol. 2020;9(7):1–10.

170. Vujosevic S, Smolek MK, Lebow KA, Notaroberto N, Pallikaris A, Casciano M. Detection of Macular Function Changes in Early (AREDS 2) and Intermediate (AREDS 3) Age-Related Macular Degeneration. Ophthalmologica. 2011;225(3):155–60.

171. Vujosevic S, Pucci P, Casciano M, Longhin E, Convento E, Bini S, et al. Long-term longitudinal modifications in mesopic microperimetry in early and intermediate age-related macular degeneration. Graefes Arch Clin Exp Ophthalmol Albrecht Von Graefes Arch Klin Exp Ophthalmol. 2017 Feb;255(2):301–9.

172. Weingessel B, Sacu S, Vécsei-Marlovits PV, Weingessel A, Richter-Mueksch S, Schmidt-Erfurth U. Interexaminer and intraexaminer reliability of the microperimeter MP-1. Eye. 2009 May;23(5):1052–8.

173. Welker SG, Pfau M, Heinemann M, Schmitz-Valckenberg S, Holz FG, Finger RP. Retest Reliability of Mesopic and Dark-Adapted Microperimetry in Patients With Intermediate Age-Related Macular Degeneration and Age-Matched Controls. Invest Ophthalmol Vis Sci. 2018 Mar 20;59(4):AMD152–9.

174. Wightman A.J., Abbott C.J., McGuinness M.B., Caruso E., Guymer R.H., Luu C.D. Presymptomatic retinal sensitivity changes in intermediate age-related macular degeneration associated with new retinal fluid. Transl Vis Sci Technol. 2019;8(6):3.

175. Wu Z, Ayton L, Guymer R, Luu C. Intrasession Test-Retest Variability of Microperimetry in Age-Related Macular Degeneration. Invest Ophthalmol Vis Sci. 2013 Nov;54(12):7378–85.

176. Wu Z, Ayton LN, Luu CD, Guymer RH. Microperimetry of nascent geographic atrophy in age-related macular degeneration. Invest Ophthalmol Vis Sci. 2014 Dec 16;56(1):115–21.

177. Wu Z, Ayton LN, Guymer RH, Luu CD. Low-luminance visual acuity and microperimetry in age-related macular degeneration. Ophthalmology. 2014 Aug;121(8):1612–9.

178. Wu Z, Ayton LN, Luu CD, Guymer RH. Relationship between retinal microstructures on optical coherence tomography and microperimetry in age-related macular degeneration. Ophthalmology. 2014 Jul;121(7):1445–52.

179. Wu Z, Ayton LN, Guymer RH, Luu CD. Comparison Between Multifocal Electroretinography and Microperimetry in Age-Related Macular Degeneration. Invest Ophthalmol Vis Sci. 2014 Oct 1;55(10):6431–9.

180. Wu Z., Guymer R.H., Jung C.J., Goh J.K., Ayton L.N., Luu C.D., et al. Measurement of retinal sensitivity on tablet devices in age-related macular degeneration. Transl Vis Sci Technol. 2015;4(3):13.

181. Wu Z, Ayton LN, Luu CD, Guymer RH. Longitudinal changes in microperimetry and low luminance visual acuity in age-related macular degeneration. JAMA Ophthalmol. 2015 Apr;133(4):442–8.

182. Wu Z, Ayton LN, Makeyeva G, Guymer RH, Luu CD. Impact of reticular pseudodrusen on microperimetry and multifocal electroretinography in intermediate age-related macular degeneration. Invest Ophthalmol Vis Sci. 2015 Mar 3;56(3):2100–6.

183. Wu Z, Cunefare D, Chiu E, Luu CD, Ayton LN, Toth CA, et al. Longitudinal Associations Between Microstructural Changes and Microperimetry in the Early Stages of Age-Related Macular Degeneration. Invest Ophthalmol Vis Sci. 2016 Jul 1;57(8):3714–22.

184. Wu Z, Guymer RH, Finger RP. Low luminance deficit and night vision symptoms in intermediate age-related macular degeneration. Br J Ophthalmol. 2016 Mar;100(3):395–8.

185. Wu Z, Luu CD, Hodgson LAB, Caruso E, Brassington KH, Tindill N, et al. Secondary and exploratory outcomes of the subthreshold nanosecond laser intervention randomized trial in age-related macular degeneration: a lead study report. Ophthalmol Retina. 2019 Dec;3(12):1026–34.

186. Wu Z, Luu CD, Hodgson LA, Caruso E, Chen FK, Chakravarthy U, et al. Examining the added value of microperimetry and low luminance deficit for predicting progression in age-related macular degeneration. Br J Ophthalmol. 2020 Apr;105(5):711–5.

187. Wu Z, Luu CD, Hodgson LAB, Caruso E, Chen FK, Chakravarthy U, et al. Using Microperimetry and Low-Luminance Visual Acuity to Detect the Onset of Late Age-Related Macular Degeneration: A Lead Study Report. Retina Phila Pa. 2021 May 1;41(5):1094–101.

188. Zhang Y, Sadda SR, Sarraf D, Swain TA, Clark ME, Sloan KR, et al. Spatial Dissociation of Subretinal Drusenoid Deposits and Impaired Scotopic and Mesopic Sensitivity in AMD. Invest Ophthalmol Vis Sci. 2022 Feb 1;63(2):32.

189. Goh KL, Abbott CJ, Hadoux X, Jannaud M, Hodgson LAB, van Wijngaarden P, et al. Hyporeflective Cores within Drusen: Association with Progression of Age-Related Macular Degeneration and Impact on Visual Sensitivity. Ophthalmol Retina. 2022 Apr;6(4):284–90.

190. Whiting P, Rutjes AWS, Reitsma JB, Bossuyt PMM, Kleijnen J. The development of quadas: a tool for the quality assessment of studies of diagnostic accuracy included in systematic reviews. BMC Med Res Methodol. 2003 Nov 10;3:25.

191. Wells G, Shea B, O’Connell D, Peterson J, Welch V, Losos M, et al. The Newcastle-Ottawa Scale (NOS) for assessing the quality of nonrandomised studies in meta-analyses [Internet]. The Ottawa Hospital Research Institute; 2013 [cited 2021 Jun 2]. Available from: http://www.ohri.ca/programs/clinical_epidemiology/oxford.asp

192. Schünemann H, Brożek J, Guyatt G, Oxman A. GRADE handbook for grading quality of evidence and strength of recommendations [Internet]. The GRADE Working Group; 2013. Available from: https://guidelinedevelopment.org/handbook

193. Guyatt G, Oxman AD, Akl EA, Kunz R, Vist G, Brozek J, et al. Grade guidelines: 1. introduction-grade evidence profiles and summary of findings tables. J Clin Epidemiol. 2011 Apr;64(4):383–94.

194. Vaz S, Falkmer T, Passmore AE, Parsons R, Andreou P. The case for using the repeatability coefficient when calculating test–retest reliability. PLOS ONE. 2013 Sep 9;8(9):e73990.

195. Beckerman H, Roebroeck ME, Lankhorst GJ, Becher JG, Bezemer PD, Verbeek AL. Smallest real difference, a link between reproducibility and responsiveness. Qual Life Res Int J Qual Life Asp Treat Care Rehabil. 2001;10(7):571–8.

196. Wong EN, Morgan WH, Chen FK. Intersession test–retest variability of 10-2 MAIA microperimetry in fixation-threatening glaucoma. Clin Ophthalmol. 2017;11:745–52.

197. Wu Z, Ayton LN, Guymer RH, Luu CD. Intrasession test-retest variability of microperimetry in age-related macular degeneration. Invest Ophthalmol Vis Sci. 2013 Nov 11;54(12):7378–85.

198. Simunovic MP, Hess K, Avery N, Mammo Z. Threshold versus intensity functions in two-colour automated perimetry. Ophthalmic Physiol Opt. 2021;41(1):157–64.

199. Hood DC, Greenstein V. Models of the normal and abnormal rod system. Vision Res. 1990;30(1):51–68.
